# Dynamic interplay between non-coding enhancer transcription and gene activity in development

**DOI:** 10.1038/s41467-023-36485-1

**Published:** 2023-02-20

**Authors:** Kota Hamamoto, Yusuke Umemura, Shiho Makino, Takashi Fukaya

**Affiliations:** 1 Laboratory of Transcription Dynamics, Research Center for Biological Visualization, Institute for Quantitative Biosciences, The University of Tokyo, Bunkyo-ku Tokyo, Japan; 2 Department of Life Sciences, Graduate School of Arts and Sciences, The University of Tokyo, Bunkyo-ku Tokyo, Japan

**Keywords:** Transcriptional regulatory elements, Non-coding RNAs

## Abstract

Non-coding transcription at the intergenic regulatory regions is a prevalent feature of metazoan genomes, but its biological function remains uncertain. Here, we devise a live-imaging system that permits simultaneous visualization of gene activity along with intergenic non-coding transcription at single-cell resolution in *Drosophila*. Quantitative image analysis reveals that elongation of RNA polymerase II across the internal core region of enhancers leads to suppression of transcriptional bursting from linked genes. Super-resolution imaging and genome-editing analysis further demonstrate that enhancer transcription antagonizes molecular crowding of transcription factors, thereby interrupting the formation of a transcription hub at the gene locus. We also show that a certain class of developmental enhancers are structurally optimized to co-activate gene transcription together with non-coding transcription effectively. We suggest that enhancer function is flexibly tunable through the modulation of hub formation via surrounding non-coding transcription during development.

## Abstract (web summary)

Non-coding transcription at the intergenic regulatory regions is a prevalent feature of metazoan genomes, but its function remains uncertain. Here the authors show that enhancer function is flexibly tunable through the modulation of hub formation via surrounding non-coding transcription.

## Introduction

Enhancers are a class of regulatory DNAs that control spatial and temporal specificity of gene expression in development^1^. Quantitative imaging and single-cell transcriptome analyses revealed that enhancers mainly act to drive successive bursts of de novo RNA synthesis from their target genes^2–4^. Recent whole-genome studies reported that non-coding transcription at the intergenic regulatory regions is a pervasive feature of the metazoan genome^5,6^. Among these, non-coding enhancer transcription is thought to be a widespread mechanism conserved across species including mammals^5–7^, flies^8,9^, and worms^10^, and often used as a hallmark for identifying active enhancers in the genome^11^. A number of studies reported that the level of enhancer transcription correlates with the activity of nearby genes^5,6,12,13^, implicating that there is a functional interplay between these two reactions to facilitate gene expression. It has also been reported that transcription start sites (TSSs) present within enhancers can function as a promoter when directly fused with a *lacZ* reporter gene in *Drosophila*^14^, yet its biological function still remains unclear. Whereas a variety of models have been proposed to explain the molecular function of enhancer transcription so far^15^, most of them stem from bulk and fixed analysis of the heterogeneous population of cultured cells, and studies that directly link non-coding enhancer transcription and gene activity at the single-cell level are scarce. The fact that live visualization of non-coding enhancer transcription in multicellular organisms has been challenging hindered the elucidation of how it impacts temporal dynamics of gene expression during animal development. In addition, it has also been implicated that non-coding transcription at the intergenic regulatory regions can lead to the downregulation of nearby genes^16–21^, which is seemingly incompatible with the proposed positive regulatory functions of enhancer transcription. More recently, the whole-genome study reported that in vivo activities of developmental enhancers negatively correlate with the level of non-coding transcription in *Drosophila*^22^. To reconcile these contradictory observations, there is a critical need for developing a new experimental framework that enables one to precisely modulate the mode of intergenic non-coding transcription and quantitatively visualize its functional impacts on gene activity in living multicellular organism.

Here, we successfully devise an MS2/PP7 live-imaging system that permits simultaneous visualization of gene activity along with intergenic non-coding transcription in developing *Drosophila* embryos. Quantitative image analysis reveals that induction of transcriptional bursting from linked target genes is dramatically suppressed in the presence of enhancer transcription *in cis*. Using a variety of genome-engineering and genetic approaches, we provide evidence that transcriptional attenuation takes place specifically when elongating RNA polymerase II (Pol II) transverses the internal core region of enhancers. Super-resolution imaging and genome-editing analysis of key transcription factors Dorsal and Zelda further demonstrate that enhancer transcription counteracts molecular crowding of transcriptional activators to limit the formation of transcription hub at the gene locus. We also show that a certain class of developmental enhancers are structurally optimized to co-activate gene transcription together with non-coding transcription at the same time. We propose that regulatory activities of developmental enhancers are flexibly tunable through the modulation of hub formation via surrounding non-coding transcription during development.

## Results

### Live-imaging of intergenic non-coding transcription in living embryos

**Fig. 1:**
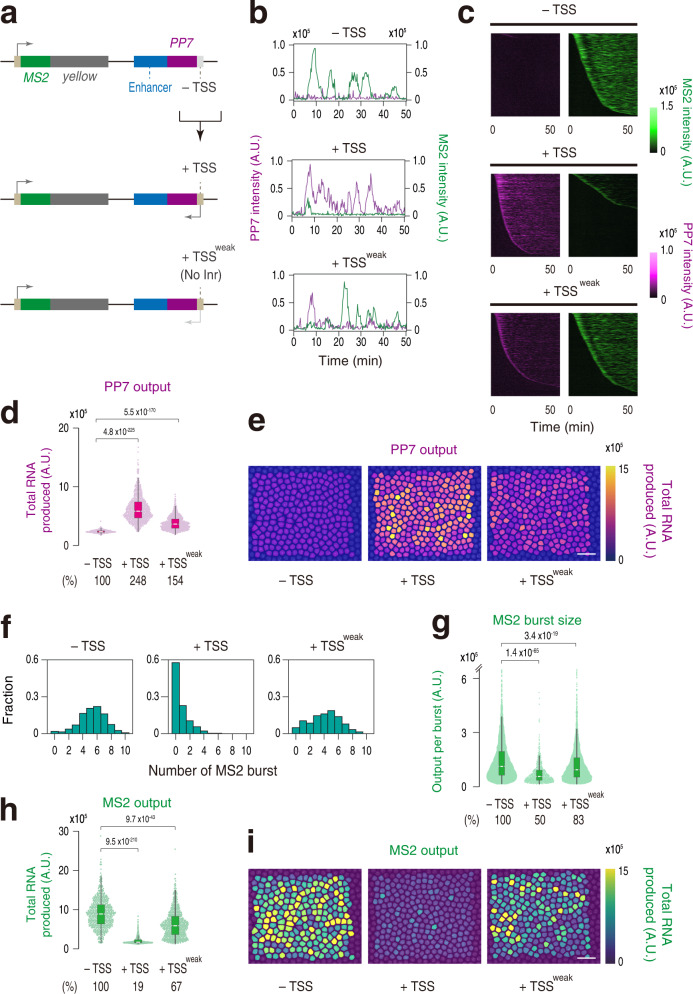
Non-coding enhancer transcription suppresses burst induction. **a** The *yellow* reporter gene containing the *Drosophila* synthetic core promoter (DSCP) and 24x MS2 repeats were placed under the control of the *sna* shadow enhancer fused with 24x PP7 repeats (top). Minimal core promoter motifs were placed adjacent to the enhancer to drive intergenic non-coding transcription (middle). Inr motif at the intergenic TSS was specifically mutated (bottom). **b** Representative trajectories of transcription activities of the reporter locus containing – TSS (top), + TSS (middle), or + TSS^weak^ (Inr mutant; bottom) at the enhancer region. **c** MS2 and PP7 trajectories for all the analyzed nuclei. Each row represents the MS2 or PP7 trajectory for a single nucleus. A total of 676, 719, and 700 ventral-most nuclei, respectively, were analyzed from three independent embryos for the reporter locus containing – TSS (top), + TSS (middle), or + TSS^weak^ (bottom) at the enhancer region. Nuclei were ordered by the onset of MS2 or PP7 transcription in nc14, separately. The same number of nuclei were analyzed hereafter. **d** Boxplot showing the distribution of the total output of PP7 transcription. The box indicates the lower (25%) and upper (75%) quantile and the white line indicates the median. Whiskers extend to the most extreme, non-outlier data points. **e** Each nucleus was colored with respect to the total output of PP7 transcription in the representative embryos. The maximum projected image of His2Av-eBFP2 is shown in gray. The image is oriented anterior to the left and the ventral view facing up. Scale bar indicates 20 μm. **f** Histograms showing the distribution of MS2 burst frequency. **g** Boxplot showing the distribution of MS2 burst size. The box indicates the lower (25%) and upper (75%) quantile and the white line indicates the median. Whiskers extend to the most extreme, non-outlier data points. A total of 3607, 536, and 2845 MS2 bursts, respectively, were analyzed for the reporter locus containing – TSS, +TSS, or + TSS^weak^ at the enhancer region. The double hash mark on the y-axis indicates that >99% of the data points are presented. **h** Boxplot showing the distribution of the total output of MS2 transcription. The box indicates the lower (25%) and upper (75%) quantile and the white line indicates the median. Whiskers extend to the most extreme, non-outlier data points. **i** Each nucleus was colored with respect to the total output of MS2 transcription in the representative embryos. The maximum projected image of His2Av-eBFP2 is shown in gray. The image is oriented anterior to the left and the ventral view facing up. Scale bar indicates 20 μm. A.U.; arbitrary unit. Percentages shown at the bottom of the boxplots represent the relative values of the median. *P* values were calculated by a two-sided Wilcoxon rank-sum test with Bonferroni correction. Source data are provided as a Source Data file.

In order to simultaneously monitor intergenic non-coding transcription along with gene activity at the single-cell resolution, we employed a recently developed MS2/PP7 two-color live-imaging method in *Drosophila*^3,23–25^. First, a sequence cassette containing 24x MS2 repeats was engineered into the 5´ untranslated region (UTR) of the *yellow* reporter gene (Fig. 1a; top). A well-characterized 1.5-kb *snail* (*sna*) shadow enhancer^26^ was placed ~7.5 kb downstream of the promoter region, which is similar to the enhancer-promoter distance at the endogenous *sna* locus. Transcription of the *MS2-yellow* is dependent on activation cue from linked enhancer since its expression is entirely abolished upon deletion of enhancer sequence from the locus^27^. For simultaneous visualization of intergenic transcription at the enhancer region, a sequence cassette containing 24x PP7 repeats was fused with the enhancer (Fig. 1a; top). CAGE-seq data shows that the basal level of non-coding transcription at the *sna* shadow enhancer is below the detection limit (Supplementary Fig. 1a). Consistent with this, enhancer-derived transcripts were not detected at the region where *sna* gene is actively transcribed by fluorescence in situ hybridization (FISH) (Supplementary Fig. 1c, d). We then examined if non-coding enhancer transcription can be triggered from this synthetic locus. A short 155-bp DNA fragment containing minimal core promoter motifs were placed adjacent to the enhancer to mimic genomic configuration around the TSSs of unidirectionally transcribed enhancers (Fig. 1a; middle)^14,22^. This engineered synthetic locus was then integrated into the same genomic landing site via phiC31-mediated transgenesis^28^. Notably, this minimal modification successfully triggered the production of non-coding transcripts from the intergenic enhancer region at the *sna* expression domain (Supplementary Fig. 1e, f). It has been previously shown that bidirectional elongation complexes seen at mammalian TSSs are less prevalent in *Drosophila*^8^. Consistent with this, the inversion of engineered TSS eliminated transcription from the enhancer region (Supplementary Fig. 1g, h), demonstrating that non-coding intergenic transcription seen in this study is actually unidirectional. Thus, the use of this system enables us to precisely modulate the mode of intergenic transcription, and quantitatively visualize its functional impact on gene activity in developing embryos.

### Non-coding enhancer transcription attenuates the expression of linked gene

We next sought to determine how the emergence of non-coding enhancer transcription impacts expression profiles of linked MS2 reporter genes in living embryos. Nascent RNA production from the MS2 reporter gene and PP7-tagged enhancer was simultaneously visualized with maternally provided MCP-GFP and mCherry-PCP fusion proteins from the entry into nuclear cycle 14 (nc14) (Supplementary Movie 1). Consistent with FISH analysis (Supplementary Fig. 1c–f), entire population of the ventral-most nuclei produced a clear PP7 signal when TSS was engineered at the intergenic region (Fig. 1b–e; – TSS vs. +TSS). We confirmed that the strength of non-coding transcription seen in our synthetic system is comparable with that of naturally transcribing endogenous *Ubx* BRE enhancer (Supplementary Fig. 2). We then analyzed expression profiles of the MS2 reporter gene in the same embryos. Unexpectedly, we observed a sharp reduction in the level of MS2 activation when transcription was triggered at the enhancer region (Fig. 1b, c and Supplementary Fig. 3a, b). Heatmap analysis revealed that there is an overall delay in activating MS2 reporter transcription in the presence of enhancer transcription (Fig. 1c). Indeed, ~60% of ventral-most nuclei never experienced MS2 transcription during the analysis while non-coding enhancer transcription occurred much more ubiquitously and rapidly (Supplementary Fig. 3c; middle). Consistent with this, there was a dramatic decrease in the frequency of MS2 burst (Fig. 1f). In addition, the size of individual MS2 burst (i.e., number of nascent transcripts produced per burst) became ~2-fold smaller upon induction of enhancer transcription (Fig. 1g). As a consequence, total output of MS2 transcription was reduced by >80% (Fig. 1h, i). This trend can be also seen when mean MS2 and PP7 intensity at each time point were measured from all the individual trajectories (Supplementary Fig. 4a). We further confirmed changes in *MS2-yellow* mRNA abundance by RT-qPCR method (Supplementary Fig. 5a, b). Essentially same results were also seen when the *sna* shadow enhancer was replaced with another well-characterized developmental enhancer, *rhomboid* neuroectoderm element (*rho* NEE)^29^ (Supplementary Figs. 1b,6). We next performed MS2/PP7 live-imaging analysis by extending the enhancer-promoter distance to ~10 kb to examine if the same suppressive effect can be seen in different genomic configurations. It is important to note that a previous whole-genome study estimated that the median enhancer-promoter distance in the *Drosophila* genome is ~10 kb^30^. Quantitative analysis of resulting visualization revealed that non-coding enhancer transcription can exert its suppressive function irrespectively of the enhancer-promoter distance (Supplementary Fig. 7). These results together give rise to the possibility that non-coding enhancer transcription somehow attenuates induction of transcriptional bursting from a linked gene in developing embryos.

It has been previously shown that Initiator (Inr) motif is enriched around the TSSs of transcribed enhancers^14,22,31,32^. Intergenic TSS used in this study also contains Inr motif. To examine if the extent of enhancer transcription correlates with its inhibitory function, we next produced a synthetic locus where Inr motif at the intergenic TSS was specifically mutated (Fig. 1a; bottom). As expected, the level of enhancer transcription was reduced upon Inr mutation (Fig. 1b–e; + TSS^weak^). Conversely, the burst profile and transcriptional output of the MS2 reporter gene were partially restored in this genomic configuration (Fig. 1f–i and Supplementary Figs. 4a,5a, b). Overall, these results are consistent with the idea that non-coding intergenic transcription can flexibly modulate the functionality of enhancers in an activity-dependent manner.

### Mechanism of transcriptional attenuation by non-coding enhancer transcription

**Fig. 2:**
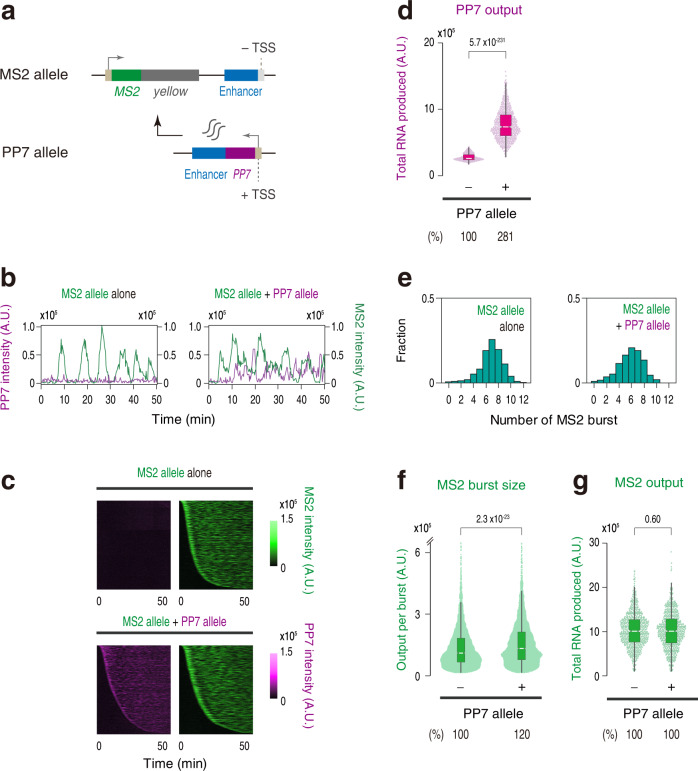
Non-coding transcripts do not affect target gene expression in trans. **a** The *MS2-yellow* reporter gene was placed under the control of a non-transcribed *sna* shadow enhancer (MS2 allele). Production of enhancer-derived non-coding transcripts was driven from the *sna* shadow enhancer located on the other homologous chromosome (PP7 allele). **b** Representative trajectories of transcription activities of the MS2 allele with (right) or without (left) the PP7 allele. **c** MS2 and PP7 trajectories for all the analyzed nuclei. Each row represents the MS2 or PP7 trajectory for a single nucleus. A total of 719 and 704 ventral-most nuclei, respectively, were analyzed from three independent embryos lacking (top) or containing (bottom) the PP7 allele. Nuclei were ordered by the onset of MS2 or PP7 transcription in nc14, separately. The same number of nuclei were analyzed hereafter. **d** Boxplot showing the distribution of the total output of PP7 transcription. The box indicates the lower (25%) and upper (75%) quantile and the white line indicates the median. Whiskers extend to the most extreme, non-outlier data points. **e** Histograms showing the distribution of MS2 burst frequency. **f** Boxplot showing the distribution of MS2 burst size. The box indicates the lower (25%) and upper (75%) quantile and the white line indicates the median. Whiskers extend to the most extreme, non-outlier data points. A total of 4917 and 4109 MS2 bursts, respectively, were analyzed for embryos lacking or containing the PP7 allele. The double hash mark on the y-axis indicates that >99% of the data points are presented. **g** Boxplot showing the distribution of the total output of MS2 transcription. The box indicates the lower (25%) and upper (75%) quantile and the white line indicates the median. Whiskers extend to the most extreme, non-outlier data points. A.U.; arbitrary unit. Percentages shown at the bottom of boxplots represent the relative values of the median. *P* values were calculated by a two-sided Wilcoxon rank-sum test. Source data are provided as a Source Data file.

Our data suggest that non-coding intergenic transcription reduces the efficiency of burst induction. To obtain mechanistic insights into this process, we first examined the possibility that non-coding transcripts themselves play a role in attenuating burst induction from the MS2 reporter gene. Indeed, there are several examples where enhancer-derived non-coding transcripts alter gene activity in trans^33,34^. According to this *trans*-acting model, it is expected that enhancer-derived non-coding transcripts produced from the PP7 allele located on the other homologous chromosome can also impact the expression profile of the MS2 allele lacking intergenic TSS in trans (Fig. 2a). Live-imaging analysis of resulting embryos revealed that the MS2 reporter gene maintains a high level of transcription irrespectively of the induction of non-coding enhancer transcription from the other allele (Fig. 2b–d and Supplementary Movie 2). Consistently, the burst profile and total output of the MS2 reporter gene remained to be mostly unchanged (Fig. 2e–g and Supplementary Fig. 4b). Thus, it is likely that enhancer-derived non-coding transcripts do not possess an ability to modulate gene activity in trans.

**Fig. 3:**
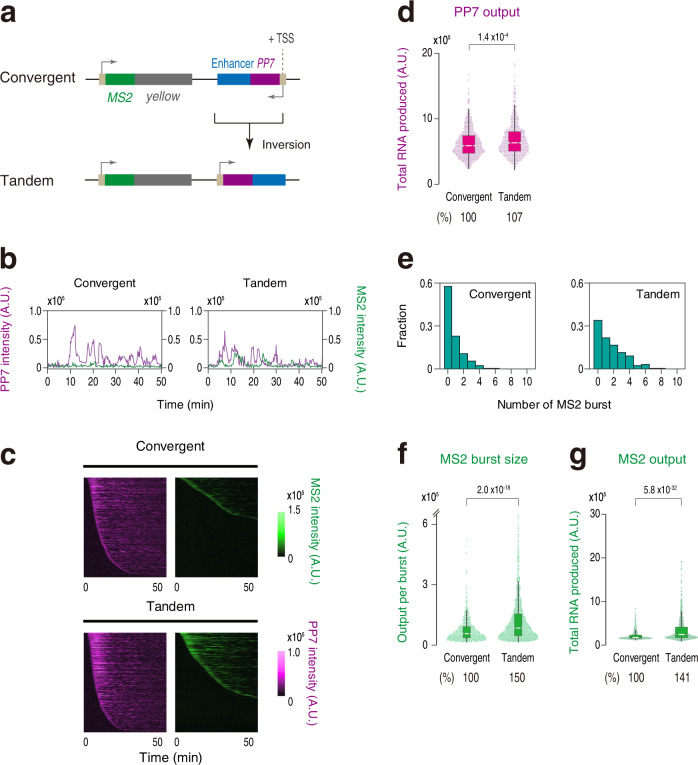
Non-coding enhancer transcription in a tandem orientation also suppresses burst induction. **a** Transcribing *sna* shadow enhancer was placed in a tandem orientation relative to the *MS2-yellow* reporter gene. **b** Representative trajectories of transcription activities of the reporter locus driving non-coding enhancer transcription in a convergent (left) or a tandem orientation (right). **c** MS2 and PP7 trajectories for all the analyzed nuclei. Each row represents the MS2 or PP7 trajectory for a single nucleus. A total of 719 and 638 ventral-most nuclei, respectively, were analyzed from three independent embryos for the reporter locus driving non-coding enhancer transcription in a convergent (top) or a tandem orientation (bottom). Nuclei were ordered by the onset of MS2 or PP7 transcription in nc14, separately. The same number of nuclei were analyzed hereafter. A panel of Convergent is the same as the panel of +TSS shown in Fig. 1c. **d** Boxplot showing the distribution of the total output of PP7 transcription. The box indicates the lower (25%) and upper (75%) quantile and the white line indicates the median. Whiskers extend to the most extreme, non-outlier data points. The plot of Convergent is the same as the plot of +TSS shown in Fig. 1d. **e** Histograms showing the distribution of MS2 burst frequency. The plot of Convergent is the same as the plot of +TSS shown in Fig. 1f. **f** Boxplot showing the distribution of MS2 burst size. The box indicates the lower (25%) and upper (75%) quantile and the white line indicates the median. Whiskers extend to the most extreme, non-outlier data points. A total of 536 and 1055 MS2 bursts, respectively, were analyzed for the reporter locus driving non-coding enhancer transcription in a convergent or tandem orientation. The double hash mark on the y-axis indicates that >99% of the data points are presented. The plot of Convergent is the same as the plot of +TSS shown in Fig. 1g. **g** Boxplot showing the distribution of the total output of MS2 transcription. The box indicates the lower (25%) and upper (75%) quantile and the white line indicates the median. Whiskers extend to the most extreme, non-outlier data points. The plot of Convergent is the same as the plot of +TSS shown in Fig. 1h. A.U.; arbitrary unit. Percentages shown at the bottom of the boxplots represent the relative values of the median. *P* values were calculated by a two-sided Wilcoxon rank-sum test. Source data are provided as a Source Data file.

We then explored the possibility that convergent transcription from the enhancer region counteracts target gene activation by allowing enhancer-transcribing Pol II to penetrate into the neighboring MS2 gene body. In our system, both the MS2 and PP7 transcription units were designed to contain a termination site at the 3´ end. In yeast, it has been reported that head-to-head collision of Pol II interrupts the expression of convergently transcribed genes^35^. To test if a similar mechanism also takes place in *Drosophila*, we produced a reporter strain that contains transcribing enhancer in a tandem orientation (Fig. 3a and Supplementary Movie 3). Only a subtle increase in PP7 activity was observed when enhancer transcription was driven in a tandem orientation (Fig. 3b–d). If PP7 transcription exerts its suppressive function regardless of the orientation relative to the linked gene *in cis*, the level of MS2 transcription is expected to be largely unchanged. Contrary to this idea, we found that MS2 reporter transcription is partially restored in a tandem orientation (Fig. 3e–g and Supplementary Fig. 4c). These results suggest that convergent enhancer transcription facing toward the neighboring transcription unit can augment suppression of transcriptional bursting from a linked gene. Importantly, however, the total output of MS2 transcription in a tandem reporter locus remained to be >70% lower compared to the original reporter locus lacking intergenic TSS (Supplementary Fig. 8). These results suggest that transcriptional readthrough or Pol II collision is not a major source of transcriptional attenuation seen in this study. In this regard, our mechanism is clearly different from transcriptional attenuation of host gene expression by intragenic enhancers in the mammalian system where Pol II collision is suggested to play a key role^36^.

**Fig. 4:**
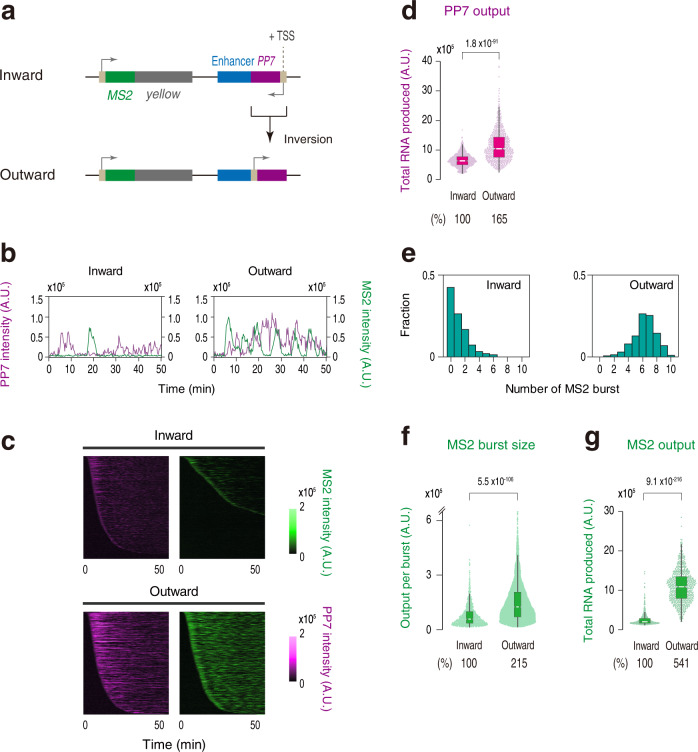
Enhancer self-transcription is required for target gene attenuation. **a** PP7 transcription unit at the enhancer region was inverted to drive non-coding transcription in an outward orientation relative to the *sna* shadow enhancer. **b** Representative trajectories of transcription activities of the reporter locus driving PP7 transcription in an inward (left) or an outward orientation (right). **c** MS2 and PP7 trajectories for all the analyzed nuclei. Each row represents the MS2 or PP7 trajectory for a single nucleus. A total of 739 and 666 ventral-most nuclei, respectively, were analyzed from three independent embryos for the reporter locus driving PP7 transcription in an inward (top) or an outward orientation (bottom). Nuclei were ordered by the onset of MS2 or PP7 transcription in nc14, separately. The same number of nuclei were analyzed hereafter. **d** Boxplot showing the distribution of the total output of PP7 transcription. The box indicates the lower (25%) and upper (75%) quantile and the white line indicates the median. Whiskers extend to the most extreme, non-outlier data points. **e** Histograms showing the distribution of MS2 burst frequency. **f** Boxplot showing the distribution of MS2 burst size. The box indicates the lower (25%) and upper (75%) quantile and the white line indicates the median. Whiskers extend to the most extreme, non-outlier data points. A total of 845 and 4220 MS2 bursts, respectively, were analyzed for the reporter locus driving PP7 transcription in an inward or an outward orientation. The double hash mark on the y-axis indicates that >99% of the data points are presented. **g** Boxplot showing the distribution of the total output of MS2 transcription. The box indicates the lower (25%) and upper (75%) quantile and the white line indicates the median. Whiskers extend to the most extreme, non-outlier data points. A.U.; arbitrary unit. Percentages shown at the bottom of the boxplots represent the relative values of the median. *P* values were calculated by a two-sided Wilcoxon rank-sum test. Source data are provided as a Source Data file.

Previous studies suggested that two linked transcription units can compete with each other for an interaction with a shared enhancer when they are placed under the control of a single enhancer^37–39^. More recently, it has also been suggested that high levels of nascent RNAs themselves act as a negative feedback mechanism to downregulate gene transcription^21^. To test if transcriptional attenuation is caused by promoter competition or RNA-mediated negative feedback mechanism, we produced a synthetic locus where PP7 transcription is driven in an outward orientation relative to the enhancer (Fig. 4a and Supplementary Movie 4). In this reporter locus, we observed even stronger PP7 transcription compared to the original reporter configuration (Fig. 4b–d). According to the promoter competition or RNA-mediated feedback hypothesis, it is expected that increased PP7 activity will lead to a further diminishment of target gene activity. However, contrary to this expectation, induction of transcriptional bursting from the MS2 reporter gene was largely restored upon inversion of PP7 transcription (Fig. 4e–g and Supplementary Figs. 4d, 5c, d, 6), suggesting that neither promoter competition nor RNA-mediated negative feedback mechanism can account for the attenuation of target gene expression seen in this study. Instead, our results strongly suggest that Pol II elongation across the internal core region of enhancer is responsible for limiting their capability of inducing transcriptional bursting from the linked gene. To further test this idea, unidirectional TSS driving non-coding transcription in an outward orientation was converted to bidirectional TSS by adding minimal core promoter elements in an inward orientation (Supplementary Fig. 9a). We found that MS2 transcription starts to be strongly re-repressed in the presence of bidirectional TSS at this synthetic locus (Supplementary Fig. 9b–g), supporting our conclusion that the directionality of intergenic non-coding transcription relative to nearby enhancers is a key determinant of its regulatory output in developing embryos.

### Modulation of hub formation via non-coding enhancer transcription

**Fig. 5:**
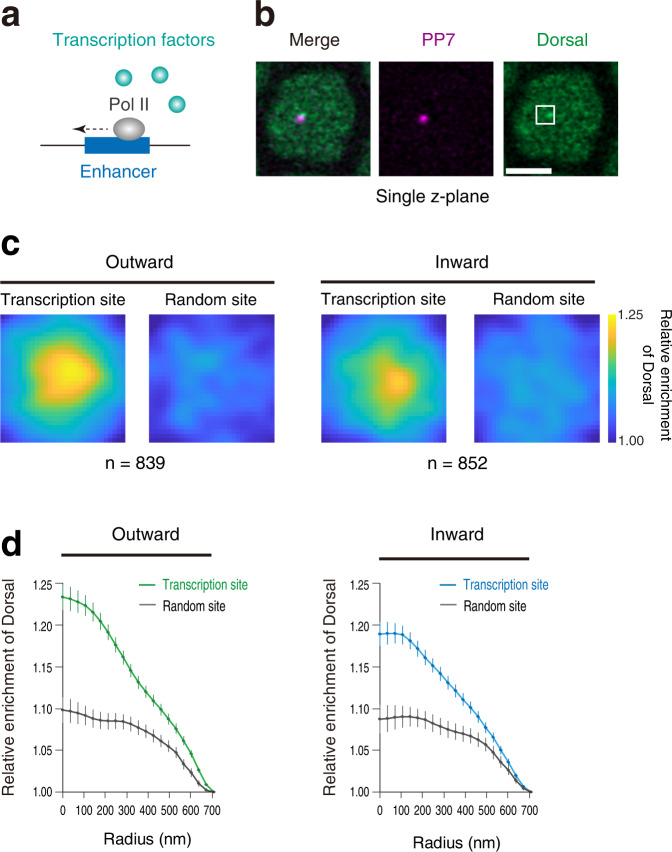
Enhancer self-transcription decreases the local concentration of the Dl activator. **a** Working hypothesis; elongating Pol II mediates eviction of transcription factors from the enhancer region. **b** Representative Airyscan images of Dorsal-GFP and PP7 transcription dots in the single z-plane containing the brightest PP7 signal. The white square indicates the analyzed region (29 × 29 pixels) centering the brightest PP7 signal. Scale bar indicates 3 μm. **c** Heatmaps showing the averaged distribution of Dorsal-GFP centering the PP7 transcription site or random site. A total of 839 and 852 PP7-transcribing nuclei, respectively, were obtained from 50 independent embryos for the reporter locus driving PP7 transcription in an outward (left) or an inward orientation (right). **d** Radial profiles of the averaged Dorsal-GFP distribution are shown in (**c**). Error bar represents the standard error of the mean. The difference between the areas of distribution curves of the transcription site was tested by a two-sided Wilcoxon rank-sum test (*p* = 0.02). Source data are provided as a Source Data file.

Then, how does Pol II elongation across the internal core enhancer region lead to attenuation of transcriptional bursting at the molecular level? Notably, recent cryo-EM studies suggested that elongating Pol II can progressively peel off template DNA from histone surfaces^40–42^. In analogy to this, we hypothesized that elongating Pol II facilitates the eviction of transcription factors from the enhancer region (Fig. 5a). As seen in the ChIP-seq profile (Supplementary Fig. 1a), the *sna* shadow enhancer used in this study is enriched with a sequence-specific transcription factor Dorsal (Dl), a homolog of mammalian NF-κB. To directly visualize how enhancer self-transcription impacts the nuclear localization of Dl, we employed CRISPR/Cas9-mediated genome-editing to fuse GFP to the C-terminus of the protein. It was confirmed that the resulting Dl-GFP strain is homozygous viable and fertile. We then monitored endogenous Dl together with the PP7 signal using the Zeiss Airyscan2 super-resolution imaging system (Fig. 5b). We first analyzed a synthetic locus where non-inhibitory PP7 transcription occurs in an outward orientation relative to the enhancer (Fig. 4a; bottom). Following previous imaging studies^43,44^, we obtained averaged profile of the Dl signal surrounding active PP7 signal over 800 nuclei from 50 independent embryos at nc14 (Supplementary Movies 5, 6). We observed a sharp enrichment of Dl signal at the site of transcription compared to randomly selected locations within nuclei (Fig. 5c, d; left). This is consistent with a recent imaging study showing that Dl forms a cluster or “hub” at the site of transcription in early embryos^45^. Our super-resolution analysis at the endogenous *sna* locus also showed that Dl is locally enriched at the site of transcription (Supplementary Fig. 10a, b), suggesting that the Dl hub we observed is not an artifact of our synthetic reporter system but rather reflects key functional properties of endogenous enhancers. We then analyzed the formation of the Dl hub at the reporter locus where inhibitory PP7 transcription is driven at the enhancer region (Fig. 4a; top). There was a clear reduction in the local concentration of Dl in this experimental setup (Fig. 5c, d; right). A similar reduction of Dl enrichment was also seen when the MS2 transcription site was used as a viewpoint of the analysis (Supplementary Fig. 11). These results together suggest that enhancer self-transcription acts to interrupt the stable association of transcription factors at the regulatory region. To further test this idea, we next examined the nuclear distribution of a pioneering transcription factor Zelda (Zld) since it was recently reported that Dl cooperates with Zld to form a hub^45^. We first verified that endogenous Zld also forms a hub at the endogenous *sna* locus (Supplementary Fig. 10c). This is an important observation as a recent study reported that the Zld hub is not clearly seen at the *hunchback-MS2* reporter locus^43^. We speculate that the degree of Zld hub formation can vary depending on the type of cooperating transcription factors and/or co-factors at each enhancer region. Super-resolution analysis revealed that our synthetic reporter system also recapitulates the formation of Zld hub seen at the endogenous *sna* locus (Supplementary Fig. 12a, b; left). As seen for Dl, the formation of Zld hub became less clear in the presence of inhibitory PP7 transcription (Supplementary Fig. 12a, b; right), supporting the idea that enhancer transcription exerts its regulatory function through the modulation of hub formation. Importantly, we noticed that the decrease in the local concentration of Dl and Zld was not as drastic as the reduction in gene activity we observed (Fig. 4). Taking into account recent studies showing that subtle changes in enhancer-promoter interactions can create large changes in transcriptional output^46,47^, changes in the size of transcription hub may also have a non-linear impact on gene activity. It is also conceivable that Pol II elongation further impairs the functional integrity of the activator hub formed at the enhancer region by interrupting subsequent recruitment of other key co-factors (see Discussion). Overall, our results are consistent with the idea that intergenic non-coding transcription modulates enhancer activity by impacting the formation and the function of the transcription hub in developing embryos.

### Naturally transcribing *Ubx* enhancer can effectively activate gene transcription

**Fig. 6:**
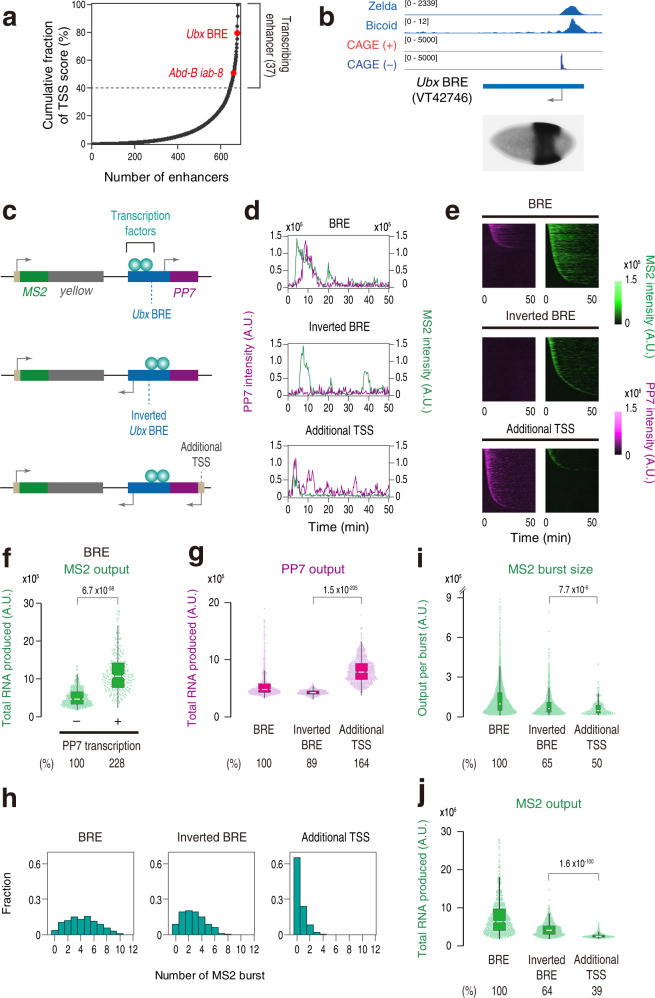
Natural Ubx BRE co-activates non-coding transcription and gene transcription effectively. **a** Cumulative fraction of TSS score for all the analyzed developmental enhancers. A total of 678 functionally validated enhancers was analyzed^30^. The dashed line represents the threshold for determining transcribed enhancers at the early developmental stage. **b** Organization of the endogenous *Ubx* BRE. Zelda ChIP-seq data from nc13 WT embryos (GSM763061)^66^, Bicoid-GFP ChIP-seq from nc14 WT embryos (GSE86966)^68^, processed 2- to 4-h WT CAGE-seq data (E-MTAB-4787)^48^ after excluding reads mapped to the coding regions were visualized with Integrative Genomics Viewer. A picture of an early embryo showing the activity of *Ubx* BRE (VT42746; ~2.2 kb in length) was taken from Fly Enhancers^30^. **c** A ~1.2-kb DNA fragment containing *Ubx* BRE was fused with 24x PP7 repeats, and placed downstream of the MS2 reporter gene (top). The orientation of the BRE-PP7 cassette was inverted (middle). Minimal core promoter motifs were placed adjacent to the enhancer to drive additional non-coding enhancer transcription (bottom). **d** Representative trajectories of transcription activities of the reporter locus containing BRE (top), inverted BRE (middle), or inverted BRE with additional TSS (bottom). **e** MS2 and PP7 trajectories for all the analyzed nuclei. Each row represents the MS2 or PP7 trajectory for a single nucleus. A total of 582, 654, and 636 nuclei, respectively, were analyzed from three independent embryos for the reporter locus containing BRE (top), inverted BRE (middle), or inverted BRE with additional TSS (bottom). Nuclei were ordered by the onset of MS2 or PP7 transcription in nc14, separately. The same number of nuclei were analyzed hereafter. **f** Boxplot showing the distribution of the total output of MS2 transcription. A total of 359 PP7 inactive and 223 PP7 active nuclei, respectively, were analyzed. The box indicates the lower (25%) and upper (75%) quantile and the white line indicates the median. Whiskers extend to the most extreme, non-outlier data points. **g** Boxplot showing the distribution of the total output of PP7 transcription. The box indicates the lower (25%) and upper (75%) quantile and the white line indicates the median. Whiskers extend to the most extreme, non-outlier data points. **h** Histograms showing the distribution of MS2 burst frequency. **i** Boxplot showing the distribution of MS2 burst size. The box indicates the lower (25%) and upper (75%) quantile and the white line indicates the median. Whiskers extend to the most extreme, non-outlier data points. A total of 2473, 1792, and 310 MS2 bursts, respectively, were analyzed for the reporter locus containing BRE, inverted BRE, or inverted BRE with additional TSS. The double hash mark on the y-axis indicates that >99% of the data points are presented. **j** Boxplot showing the distribution of the total output of MS2 transcription. The box indicates the lower (25%) and upper (75%) quantile and the white line indicates the median. Whiskers extend to the most extreme, non-outlier data points. A.U.; arbitrary unit. Percentages shown at the bottom of the boxplots represent the relative values of the median. *P* values were calculated by the two-sided Wilcoxon rank-sum test. Source data are provided as a Source Data file.

Our experiments so far focused on the analysis of synthetically engineered enhancers. We next sought to visualize activities of naturally transcribing enhancers during early embryogenesis. For this purpose, we first analyzed publicly available 2–4 h CAGE-seq dataset^48^ to quantify read counts for >650 functional enhancers that are reliably assigned to their target genes in fly embryos^30^, resulting in the identification of highly transcribing enhancers at this developmental stage (Fig. 6a). We found that enhancers regulating the expression of key developmental genes such as Hox genes *Ultrabithorax* (*Ubx*) and *Abdominal-B* (*Abd-B*) represent one of the most actively transcribing enhancers (Fig. 6a and Supplementary Table 1). Among these, we were particularly interested in the *Ubx* enhancer since this region corresponds to the classical *bx* region enhancer (BRE) that is known to be required for *Ubx* expression at the middle part of embryos (Fig. 6b; bottom)^49^ and for the development of metathoracic segment in adult flies^50^. A sharp and selective CAGE-seq peak was seen for one specific DNA strand at this region (Fig. 6b; top), indicating that BRE undergoes unidirectional non-coding transcription in early embryos. Intriguingly, we noticed that unidirectional transcription initiated from BRE is facing toward the opposite orientation relative to the transcription factor binding sites within this enhancer (Fig. 6b; top), giving rise to a possibility that BRE is naturally optimized to drive non-coding transcription in a manner that does not impede functions of bound transcription factors. To experimentally test this idea, we produced a reporter locus containing the MS2 reporter gene placed under the control of the PP7-tagged BRE (Fig. 6c; top). The single-molecule FISH analysis confirmed that our BRE reporter locus produces essentially the same expression pattern as previously reported (Supplementary Fig. 13a; compare with Fig. 6b). Consistent with the CAGE-seq profile (Fig. 6b), we reproducibly observed a clear PP7 signal originating from BRE (Fig. 6d, e and Supplementary Movies 7, 8). Importantly, BRE was found to drive a high level of transcription from the MS2 reporter gene (Fig. 6d, e), supporting the idea that BRE accommodates non-coding enhancer transcription in a manner that does not impede the activation of the linked gene. We next divided nuclei into two groups according to the presence or absence of PP7 transcription, and compared MS2 profiles between them. Intriguingly, BRE was found to more effectively activate linked MS2 reporter gene in the presence of PP7 transcription (Fig. 6f and Supplementary Fig. 13b), implicating that the natural configuration of BRE permits non-coding enhancer transcription to exert positive regulatory function. We next engineered this synthetic locus to invert the orientation of the BRE-PP7 cassette (Fig. 6c; middle). As expected, the PP7 signal was not seen in this setup (Fig. 6d, e; inverted BRE). In addition, we observed a partial reduction in the level of MS2 activity (Fig. 6d, e; inverted BRE), which is consistent with our preceding result showing that non-coding transcription in a convergent orientation leads to minor suppression of gene activity (Fig. 3). We then engineered BRE to contain additional intergenic TSS to foster Pol II elongation across the transcription factor binding sites within the enhancer (Fig. 6c; bottom). Intriguingly, this led to a dramatic reduction in the efficiency of burst induction (Fig. 6d, e, g, h; additional TSS) and the level of total RNA production from the MS2 reporter gene (Fig. 6j). Super-resolution imaging analysis further revealed that there is a reduction in the assembly of Zld hub at the site of transcription (Supplementary Fig. 14). Statistical difference of Zld hub formation was found to be <0.05 (Wilcoxon rank-sum test; *p* = 0.01) at the *Ubx* BRE (Supplementary Fig. 14b). As a comparison, the difference of Zld hub formation was less clear at the engineered *sna* shadow enhancer (Wilcoxon rank-sum test; *p* = 0.42) (Supplementary Fig. 12b), implicating that the degree of transcription factor eviction by non-coding transcription can vary depending on the configuration of each enhancer. Overall, our results are consistent with the idea that the natural configuration of BRE enables cells to effectively activate gene transcription together with non-coding transcription at the same time. Intriguingly, a similar configuration was seen for other transcribing enhancers identified in this study (Supplementary Fig. 15). Thus, it is tempting to speculate that configurations of non-coding TSSs have been evolutionarily defined, in part, by their orientation relative to the nearby transcription factor binding sites and accompanying regulatory effects at each genomic locus (see Discussion).

Among the list of transcribing enhancers we identified (Supplementary Table 1), we noticed that *hairy* enhancer contains sharp CAGE-seq peak facing toward the transcription factor binding sites within the enhancer (Supplementary Fig. 16a). To experimentally test if non-coding transcription from *hairy* enhancer affects gene expression in a suppressive manner, we produced an MS2 reporter locus containing *hairy* enhancer fused with a PP7 cassette (Supplementary Fig. 16b). Consistent with CAGE-seq data (Supplementary Table 1), we observed moderate PP7 signal together with MS2 gene transcription at the middle part of embryos (Supplementary Fig. 16c). We then compared instantaneous MS2 activities in the presence or absence of PP7 signal for all the PP7 active nuclei. It turned out that the MS2 signal becomes significantly weaker when non-coding PP7 transcription is taking place at the same time (Supplementary Fig. 16d). In contrast, PP7 transcription originating from natural *Ubx* BRE accompanied by an increase of MS2 signal intensity (Supplementary Fig. 16e). Overall, these results are consistent with the idea that intergenic non-coding transcription contributes to the modulation of gene activities either negatively or positively in an orientation-dependent manner. Our computational analysis estimates that nearly 25% of active enhancers at the early embryonic stage contain nearby TSSs facing toward the transcription factor binding sites (Supplementary Fig. 16f). This is consistent with previous whole-genome analysis showing that there is a weak yet significant negative correlation between developmental enhancer activities and the level of non-coding transcription in *Drosophila*^22^.

### Acquisition of novel TSS dramatically changes the activity of the key developmental gene

**Fig. 7:**
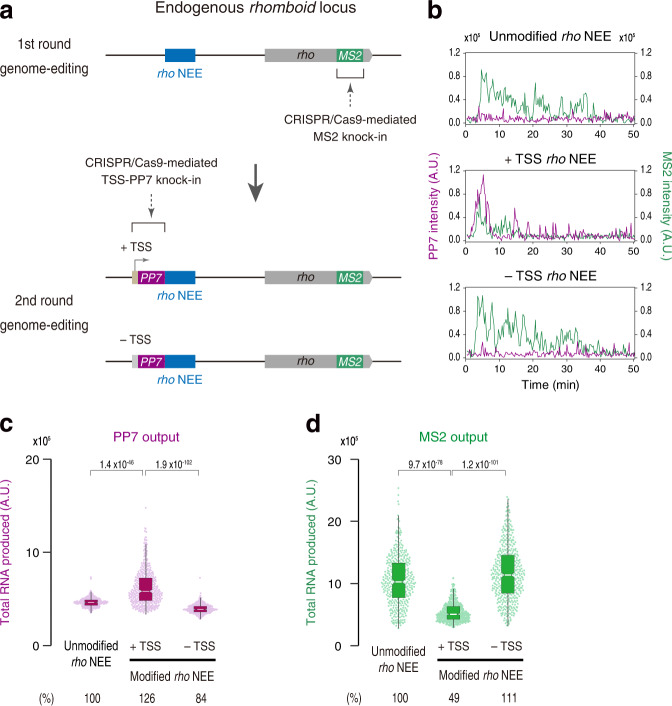
Acquisition of novel TSS dramatically reduces endogenous rho expression. **a** 24x MS2 repeats were inserted into the 3´ UTR of endogenous *rho* gene (top). Minimal core promoter motifs and 24x PP7 repeats were placed adjacent to the endogenous *rho* NEE to drive intergenic non-coding transcription (middle). As a control, 24x PP7 repeats alone were fused with *rho* NEE (bottom). **b** Representative trajectories of transcription activities of the endogenous *rho* locus containing unmodified *rho* NEE (top), +TSS *rho* NEE (middle), or −TSS *rho* NEE (bottom). **c** Boxplot showing the distribution of the total output of PP7 transcription. The box indicates the lower (25%) and upper (75%) quantile and the white line indicates the median. Whiskers extend to the most extreme, non-outlier data points. A total of 390, 419, and 448 nuclei, respectively, were analyzed from three independent embryos for the *rho-MS2* locus containing unmodified *rho* NEE, +TSS *rho* NEE, or −TSS *rho* NEE. The same number of nuclei were analyzed hereafter. **d** Boxplot showing the distribution of the total output of MS2 transcription. The box indicates the lower (25%) and upper (75%) quantile and the white line indicates the median. Whiskers extend to the most extreme, non-outlier data points. A.U.; arbitrary unit. Percentages shown at the bottom of the boxplots represent the relative values of the median. *P* values were calculated by a two-sided Wilcoxon rank-sum test with Bonferroni correction. Source data are provided as a Source Data file.

Lastly, we examined if the regulatory mechanism described in this study can be applied to modulate the activity of key developmental genes at the endogenous locus. To this end, we decided to take a strategy of multi-step CRISPR/Cas9-mediated genome-editing (Fig. 7a). As a model, we focused on the endogenous *rho* locus because its expression at the dorsal neurogenic ectoderm is solely dependent on the upstream *rho* NEE^29,51^, making interpretation of the data simple. As an initial step, the first round of CRISPR/Cas9-mediated genome-editing was carried out to insert an MS2 sequence cassette into the 3´ UTR of the transcription unit to visualize endogenous *rho* transcription in living embryos (Fig. 7a; top). It was confirmed that the resulting *rho-MS2* fly is homozygous viable and fertile. Subsequently, second round genome-editing was performed using *rho-MS2* strain to drive intergenic non-coding transcription across the endogenous *rho* NEE by placing a minimal TSS and a PP7 sequence cassette (Fig. 7a; bottom). Consistent with our preceding results, the live-imaging analysis revealed that endogenous *rho* expression becomes significantly weaker when the intergenic region acquires a novel TSS that drives non-coding transcription across the enhancer region (Fig. 7b–d). ChIP-qPCR analysis also suggests that the acquisition of novel TSS reduces Dl binding by increasing Pol II association at the endogenous *rho* NEE (Supplementary Fig. 17). Thus, we propose that the acquisition of novel intergenic TSS has contributed to facilitate diversification of enhancer activities during the process of animal evolution (see Discussion).

## Discussion

In this study, we have successfully established a live-imaging system that permits simultaneous visualization of non-coding enhancer transcription together with gene activity at the single-cell resolution in *Drosophila*. By combining a series of genome-engineering and genetic approaches, we have provided several lines of evidence that intergenic non-coding transcription can flexibly modulate enhancer function in an orientation- and activity-dependent manner (Figs. 1–4). Super-resolution imaging and genome-editing analysis further demonstrated that enhancer self-transcription impacts molecular crowding of transcription factors and hub formation at the gene locus (Fig. 5 and Supplementary Figs. 12, 14). We speculate that elongating Pol II acts as a steric hindrance for the stable association of transcription factors with their cognate enhancers, which may further interrupt subsequent recruitment of co-activators and other key transcriptional apparatus to the gene locus. Consistent with our results, it has been previously reported that non-coding transcription at the intergenic regulatory region suppresses the expression of the *SER3* gene by inducing the dissociation of transcription factors from their binding sites in yeast^19,20^. Transcription of the yeast *ADH1* gene is also shown to be suppressed through a similar mechanism during zinc starvation^16^. Similarly, in *Drosophila*, there are several examples where readthrough non-coding transcription toward the promoter region of nearby genes directly represses their expression^17,18,52^. In these cases, readthrough non-coding transcription is thought to physically interfere with the assembly of the pre-initiation complex and subsequent recruitment of Pol II to the promoter region. This so-called “transcription interference” mechanism is suggested to occur also in mammalian systems^53^. Another well-known example of transcriptional attenuation mediated by non-coding transcription is the regulation of *GAL10* and *GAL4* genes in yeast system^54^. Under the uninducible condition, non-coding RNA transcribed from the antisense *GAL10* region is required for preventing transcriptional leakage of *GAL10* and *GAL4* genes presumably through a transcriptional interference mechanism. In this regard, the mechanism described in this study is unique in that intergenic non-coding transcription at the distal enhancer region counteracts the assembly of transcription hubs, thereby remotely influencing the efficiency of burst induction from the linked gene in an orientation- and activity-dependent manner. Our data together with these preceding studies implicate that attenuation of gene activity by non-coding enhancer transcription is an ancient mechanism conserved across species. We speculate that the directionality and/or strength of intergenic non-coding transcription has been, in part, evolutionally defined as a consequence of the natural selection of sequence variations at non-coding genomic regions based on their functional impacts on nearby gene expression and associating physiological consequences in the natural environment. Acquisition of novel intergenic TSSs may also have contributed to the diversification of enhancer function during the process of animal evolution as we were able to produce a broad spectrum of regulatory activities from the same enhancer just by engineering the mode of intergenic non-coding transcription. This idea was actually supported by the analysis of endogenous *rho* locus as its gene activity was dramatically changed just by placing a minimal TSS at the intergenic region (Fig. 7).

As exemplified in the analysis of *Ubx* BRE (Fig. 6), some enhancers appear to preferentially contain non-inhibitory TSSs facing toward the opposite orientation relative to the transcription factor binding sites. Under its natural enhancer configuration, non-coding transcription from BRE was found to positively correlate with bursting activities of the linked gene (Fig. 6f and Supplementary Fig. 16e), giving rise to the possibility that there are a class of enhancers that utilize non-coding transcription to facilitate gene expression. Our super-resolution imaging analysis indicates that enhancer transcription exerts its negative regulatory function by interrupting the formation and the function of the transcription hub at the gene locus when Pol II elongation is engineered to occur toward the transcription factor binding sites in our synthetic locus (Fig. 5 and Supplementary Figs. 12, 14). Taking this into consideration, it is also conceivable that enhancer transcription exerts its positive regulatory function by facilitating the release of enhancer-bound repressors and/or inhibitory nucleosomes in certain circumstances. Importantly, our data showed that the positive effects of BRE non-coding transcription can be easily lost by placing additional intergenic TSS facing toward the transcription factor binding sites (Fig. 6), suggesting that the regulatory outcome of enhancer transcription is highly dependent on its surrounding genomic context. Indeed, unlike natural *Ubx* BRE, non-coding transcription originating from natural *hairy* enhancer was shown to exert negative regulatory function in early embryos (Supplementary Fig. 16d). Thus, we speculate that mode of enhancer transcription has been evolutionally defined by the balance between its negative and positive regulatory effects at each genomic locus. It should be noted that *Drosophila* enhancers are classified into two functionally distinct groups; developmental and housekeeping^55^, and they largely differ in their binding partners at the molecular level. In this regard, Henriques et al. pooled these two types of enhancers nondiscriminatory, and reported that there is a weak positive correlation (*ρ* = 0.24) between the level of non-coding transcription and the enhancer strength^32^. Importantly, however, a subsequent study by Rennie et al. separately analyzed housekeeping enhancers and developmental enhancers, and found that activities of developmental enhancers negatively correlate with the level of enhancer transcription (*ρ* = −0.20), while housekeeping enhancers exhibit a positive correlation^22^. In agreement with this, our analysis estimated that developmental enhancers in early embryos are more prone to contain inward TSS (Supplementary Fig. 16f). It might be possible that the positive regulatory function of non-coding transcription plays a more important role in the control of housekeeping gene expression in *Drosophila*.

Another key observation in this study is the suppression of transcriptional bursting from the linked gene in the presence of bidirectional TSS nearby from the enhancer region (Supplementary Fig. 9) as enhancer transcription typically takes place bidirectionally in mammalian genomes^5^. In this regard, the recent computational analysis suggested that a substantial fraction of mammalian enhancers are depleted of transcription factor binding sites at the origin of non-coding enhancer transcription^56^, implicating that their natural genomic configurations are organized to minimize potential inhibitory effects of non-coding transcription. Clearly, future studies are needed to fully elucidate the biological roles of enhancer transcription by visualizing its regulatory function in the context of the endogenous genome in various species. We believe that our study will serve as a critical starting point toward a full understanding of multifaced functions of non-coding enhancer transcription in the control of gene expression during animal development.

## Methods

### Experimental model

In all experiments, we studied *Drosophila melanogaster* embryos at nuclear cycle 14. The following fly lines were used in this study: *nanos* > *NLS-mCherry-PCP-NES, His2Av-eBFP2/CyO; nanos* > *MCP-GFP*^57^*, nanos* > *NLS-mCherry-PCP-NES, His2Av-eBFP2/CyO*^57^*, nanos* > *NLS-mCherry-MCP, nanos* > *NLS-mTagBFP2-PCP* (this study)*, DSCP-MS2-yellow-sna shadow enhancer-PP7-No TSS* (this study), *DSCP-MS2-yellow-sna shadow enhancer-PP7-TSS* (this study), *DSCP-MS2-yellow-sna shadow enhancer-PP7-Inverted TSS* (this study), *DSCP-MS2-yellow-sna shadow enhancer-PP7-TSS*^*weak*^ (this study), *DSCP-MS2-yellow-sna shadow enhancer*^58^, *TSS-PP7-sna shadow enhancer* (this study), *DSCP-MS2-yellow*-*TSS-PP7-sna shadow enhancer* (this study), *DSCP-MS2-yellow-sna shadow enhancer*-*TSS-PP7* (this study), *DSCP-MS2-yellow-Ubx BRE-PP7* (this study), *DSCP-MS2-yellow-inverted Ubx BRE-PP7* (this study), *DSCP-MS2-yellow-inverted Ubx BRE-PP7-TSS* (this study), *DSCP-MS2-yellow-rho NEE-PP7-No TSS* (this study), *DSCP-MS2-yellow-rho NEE-PP7-TSS* (this study), *DSCP-MS2-yellow-rho NEE*-*TSS-PP7* (this study), *DSCP-MS2-yellow-hairy enhancer-PP7* (this study), *dorsal-GFP* (this study), *zelda-GFP* (this study), *sna-MS2* (this study), *rho-MS2* (this study), *DSCP-MS2-yellow-2.5* *kb spacer-sna shadow enhancer-PP7-No TSS* (this study), *DSCP-MS2-yellow-2.5* *kb spacer-sna shadow enhancer-PP7-TSS* (this study), *DSCP-MS2-yellow-sna shadow enhancer-TSS*^*inverted*^*-TSS-PP7* (this study), *No TSS-PP7-rho NEE-rho-MS2* (this study)*, TSS-PP7-rho NEE-rho-MS2* (this study).

### Plasmids

Plasmid construction is detailed in the supplementary information.

### Site-specific transgenesis by phiC31 system

All the reporter plasmids and NLS-mCherry-MCP expressing plasmid were integrated into a unique landing site on the third chromosome using VK00033 strain^59^. phiC31 was maternally provided using *vas-phiC31* strain^60^. Microinjection was performed as previously described^61^. In brief, 0–1 h embryos were collected and dechorionated with bleach. Aligned embryos were dried with silica gel for ~8 min and covered with FL-100-1000CS silicone oil (Shin-Etsu Silicone). Subsequently, microinjection was performed using FemtoJet (Eppendorf) and DM IL LED inverted microscope (Leica) equipped with M-152 Micromanipulator (Narishige). The injection mixture typically contains ~900 ng/μl plasmid DNA, 5 mM KCl, 0.1 mM phosphate buffer, pH 6.8. mini-white marker was used for screening.

### CRISPR/Cas9-mediated genome-editing

To endogenously label Dorsal and Zelda with GFP, corresponding pCFD3 gRNA expression plasmid and pBS-GFP-3xFLAG-3xP3-dsRed donor plasmid were co-injected using either *nanos-Cas9* or *nanos-Cas9*/*CyO* strain^62^. The injection mixture typically contains ~500 ng/μl pCFD3 gRNA expression plasmid and ~800 ng/μl pBS-GFP-3xFLAG-3xP3-dsRed donor plasmid. 3xP3-dsRed was used for screening. Resulting *dorsal-GFP* flies were crossed with *y[1] w[67c23] P{y[+mDint2]=Crey}1b; sna[Sco]/CyO* (BDSC #766) to remove 3xP3-dsRed marker from the locus. For MS2-tagging of endogenous *snail* and *rhomboid* genes, corresponding pCFD3 gRNA expression plasmid and pBS-MS2-3xP3-dsRed donor plasmid were co-injected using either *nanos-Cas9* or *nanos-Cas9*/*CyO* strain^57^. The injection mixture typically contains ~500 ng/μl pCFD3 gRNA expression plasmid and ~500 ng/μl pBS-MS2-3xP3-dsRed donor plasmid. 3xP3-dsRed marker was used for screening. For endogenous *rho* NEE editing, two pCFD3 gRNA expression plasmids, pBS-Hsp70-Cas9 plasmid (addgene #46294) and pBS-3xP3-GFP-TSS/No TSS-PP7-*rho* NEE donor plasmid were co-injected to homozygous *rho-MS2* embryos. The injection mixture contains ~200 ng/μl pCFD3 gRNA expression plasmids, ~300 ng/μl pBS Cas9 expression plasmid, and ~400 ng/μl donor plasmid. 3xP3-GFP was used for screening. Microinjection was performed as described above.

### Preparation of probes for in situ hybridization

Antisense RNA probes labeled with digoxigenin (DIG RNA Labeling Mix 10 × conc, Roche) or biotin (Biotin RNA Labeling Mix 10 × conc, Roche) were transcribed using in vitro Transcription T7 Kit (Takara). Template DNA for *sna* probe was PCR amplified from genomic DNA using primers (5′-CGTAATACGACTCACTATAGGGCAGTTGGCTTAACAGTACTG-3′) and (5′-ACCTGTCACAGCCACCTCAGC-3′). Template DNA for *sna* shadow enhancer probe was PCR amplified from pbphi-TSS-PP7-*sna* shadow enhancer plasmid using primers (5′-GCATTGAGGTGTTTTGTTGGTCAAC-3′) and (5′-CGTAATACGACTCACTATAGGGTAAATTCCGATTTTTCTTGT-3′).

### Fluorescence in situ hybridization

Embryos were dechorionated and fixed in fixation buffer (1 ml of 5x PBS, 4 ml of 37% formaldehyde, and 5 ml of Heptane) for ~25 min at room temperature. The aqueous solution layer was removed and ~10 ml 100% methanol was added, and then shaken for ~1 min to devitellinize embryos. Devitellinized embryos were washed with methanol, and then stored in methanol at −30 °C before use. Antisense RNA probes labeled with digoxigenin (for *sna* shadow enhancer) and biotin (for *sna* gene) were used. Hybridization was performed at 55 °C overnight in hybridization buffer (50% formamide, 5x SSC, 50 μg/ml Heparin, 100 μg/ml salmon sperm DNA, 0.1% Tween-20). Subsequently, embryos were washed with hybridization buffer at 55 °C and incubated with Western Blocking Reagent (Roche) at room temperature for ~2 h. Then, embryos were incubated with 1:1000 dilution of sheep anti-digoxigenin (Roche, Cat# 11093274910) and mouse anti-biotin (Invitrogen, Cat# 03-3700) primary antibody at 4 °C overnight, followed by incubation with 1:1000 dilution of Alexa Fluor 555 donkey anti-sheep (Invitrogen, Cat# A-21436) and Alexa Fluor 488 donkey anti-mouse (Invitrogen, Cat# A-21202) fluorescent secondary antibody at room temperature for ~2 h. Embryos were mounted in ProLong Gold Antifade Mountant (Thermo Fisher Scientific). Imaging was performed on a Zeiss LSM 900 confocal microscope. Plan-Apochromat 20x/0.8 N.A. objective was used. Images were acquired with the following settings: 1024 × 1024 pixels, 16-bit depth, and 15 z-slices separated by 0.5 μm. The maximum projection was obtained for all z-sections, and the resulting image was rotated with the bilinear interpolation method. The brightness of images was linearly adjusted using Fiji.

### Single-molecule inexpensive FISH

Single-molecule inexpensive FISH (smiFISH) was performed as previously described^63^. Embryos were dechorionated and fixed in fixation buffer (4 ml of PBS, 1 ml of 37% formaldehyde, and 5 ml of Heptane) for ~45 min at room temperature. The aqueous solution layer was removed and ~10 ml 100% methanol was added, and then shaken for ~1 min to devitellinize embryos. Devitellinized embryos were washed with methanol, and then stored in methanol at −30 °C before use. smiFISH probes targeting the *yellow* reporter gene were designed using the Biosearch Technologies Stellaris RNA FISH probe designer tool (https://www.biosearchtech.com/support/education/stellaris-rna-fish). The following sequence was added to the 5′ end of each 20 nt probe: 5′-CCTCCTAAGTTTCGAGCTGGACTCAGTG-3′. This is the reverse complement of the X FLAP sequence used in ref. ^64^. The X FLAP sequence (5′-CACTGAGTCCAGCTCGAAACTTAGGAGG-3′) that contains Cy3 both at the 5′ and 3′ ends was synthesized by Eurofins Genomics. Probes and the X FLAP were annealed, and then stored at −30 °C before use. Hybridization was performed at 37 °C overnight in hybridization buffer (10% w/v dextran sodium sulfate 5000, 2x SSC, 10% deionized formamide, in nuclease-free H_2_O). Subsequently, embryos were washed with wash buffer (2x SSC, 10% deionized formamide in nuclease-free H_2_O) at 37 °C. After washing and DAPI staining, embryos were mounted in ProLong Gold Antifade Mountant (Thermo Fisher Scientific). Imaging was performed on a Zeiss LSM 900 confocal microscope. Plan-Apochromat 20x/0.8 N.A. objective was used. Images were acquired with the following settings: 1024 × 1024 pixels, 16-bit depth, 25 z-slices separated by 0.6 μm. The maximum projection was obtained for all z-sections, and the resulting image was rotated with the bilinear interpolation method. The brightness of images was linearly adjusted using Fiji. Probe sequences are provided in Supplementary Table 2.

### RNA extraction and RT-qPCR

Fly cages were cleared 1 h prior to embryo collection. Then collected 0–1 h embryos were allowed to develop for 2 h at room temperature. Subsequently, nc14 embryos were manually collected under the microscope, and they were homogenized in TRI Reagent (Molecular Research Center) and stored at −80 °C. Total RNA was then extracted according to the manufacturer’s protocol and cDNA was synthesized using PrimeScript Reverse Transcriptase (Takara) and oligo-dT primer. qPCR reaction was carried out using KAPA SYBR Fast qPCR Kit (KAPA Biosystems) and LightCycler 480 System II (Roche). The relative copy number of samples were calculated by LightCycler 480 software (release 1.5.1.62). The following primers were used: *yellow*, 5′-TGGCACTCATCAATGCCGTA-3′ and 5′-CAGACAGCAAGA AAACGGGC-3′; *rp49*, 5′-TACAGGCCCAAGATCGTGAA-3′ and 5′-TCCAAGAAGCGCAAGGAGA-3′. The expression level of *yellow* was calculated using the standard curve method. *rp49* was used as an internal control.

### ChIP-qPCR

After pre-laying, 2–3 h embryos were dechorionated and shaken in a mixture of 2 ml of (PBS, 0.5% Triton X-100, 6 ml of heptane, and 310 μl of 37% formaldehyde) for 5 min at room temperature. Then, 5 ml of (PBS, 0.5% Triton X-100, and 125 mM glycine) was added to quench the cross-linking 10 min after the addition of formaldehyde and shaken for 1 min at room temperature. After washing three times with (PBS, 0.5% Triton X-100), fixed embryos were flash-frozen in liquid nitrogen and stored at −80 °C. Embryos thawed on the ice were homogenized in buffer A1 (15 mM HEPES pH 7.5, 15 mM NaCl, 60 mM KCl, 4 mM MgCl_2_, 0.5% Triton X-100, 0.5 mM DTT, 1x EDTA-free Protease Inhibitor (Nacalai)). The homogenate was centrifuged at 400×*g* for 2 min at 4 °C. The supernatants were collected and centrifuged at 15,000×*g* for 5 min at 4 °C. The pellet was resuspended in buffer A2 (15 mM HEPES pH 7.5, 140 mM NaCl, 1 mM EDTA, 0.5 mM EGTA, 1% Triton X-100, 0.1% sodium deoxycholate, 0.1% SDS, 0.5% *N*-lauroylsarcosine, 1x EDTA-free Protease Inhibitor (Nacalai)) and sonicated using the Bioruptor Pico (Diagenode; 30 sec ON/30 s OFF, ten cycles). Then, sonicated samples were centrifuged at 15,000×*g* for 10 min at 4 °C, and supernatants were collected. For pre-blocking, protein G magnetic beads (Invitrogen) were washed three times with (PBS, 0.5% BSA). For pre-clearance, the chromatin extract was incubated with protein G magnetic beads for 2 h at 4 °C and supernatant was collected using a magnetic stand. For immunoprecipitation, protein G magnetic beads were preincubated with 1.1 μg of antibodies (anti-Dorsal, DSHB, Cat# 7A4; anti-RNA Pol II, Millipore, CTD4H8, Cat# 05-623) in (PBS, 0.5% BSA) over 8 h at 4 °C. The chromatin extract was incubated with a pre-mixed magnetic beads-antibody mixture overnight at 4 °C. The beads were washed four times with RIPA buffer (50 mM HEPES pH 7.5, 500 mM LiCl, 1 mM EDTA, 1% NP-40, 0.7% sodium deoxycholate) and then once with ice-cold TE50 buffer (50 mM Tris-HCl pH 8.0, 10 mM EDTA). Elution buffer (TE50 buffer, 1% SDS) was used to resuspend the beads, and immunoprecipitated samples were eluted by incubating at 65 °C for 40 min. Then, ChIP samples and input samples were reverse crosslinked by incubating at 65 °C overnight. TE buffer (10 mM Tris-HCl pH 8.0, 1 mM EDTA) containing RNaseA (final concentration of 10 μg/ml, Sigma) was added and incubated at 37 °C for 1 h, and then treated with Protease K (100-fold diluted, Takara) at 55 °C for 2 h. Samples were purified by phenol/chloroform extraction and ethanol precipitation. qPCR was performed as described above. The following primers were used for the analysis: 5′-GGAAAAGCCCACGTCCTACC-3′ and 5′-GCCACCGTACCAAAGCAATC-3′. Enrichment of Dorsal and RNA polymerase II was expressed as the percentage of input chromatin.

### Preparation of embryos for live-imaging

For MS2/PP7 two-color live-imaging, virgin females of *nanos* > *NLS-mCherry-PCP-NES, His2Av-eBFP2/CyO*; *nanos* > *MCP-GFP* were mated with homozygous males carrying the MS2/PP7 reporter allele. In Fig. 2, virgin females of *nanos* > *NLS-mCherry-PCP-NES, His2Av-eBFP2/CyO*; *nanos* > *MCP-GFP* were first crossed with homozygous males carrying the PP7 allele. Virgin females of resulting trans-heterozygote were then mated with homozygous males carrying the MS2 allele. For Airyscan imaging of the PP7 transcription site, virgin females of *nanos* > *NLS-mCherry-PCP-NES, His2Av-eBFP2/CyO* were first crossed with homozygous males of *dorsal-GFP* or *zelda-GFP*. Virgin females of resulting trans-heterozygote were then mated with homozygous males carrying the MS2/PP7 reporter allele. For Airyscan imaging of the MS2 transcription site, virgin females of *nanos* > *NLS-mCherry-MCP, nanos* > *NLS-mTagBFP2-PCP* were first crossed with homozygous males of *dorsal-GFP* or *zelda-GFP*. Virgin females of resulting trans-heterozygote were then mated with homozygous males carrying the MS2/PP7 reporter allele. The resulting embryos were dechorionated and mounted between a polyethylene membrane (Ube Film) and a coverslip (18 mm × 18 mm), and embedded in FL-100-450CS (Shin-Etsu Silicone).

### MS2/PP7 two-color live-imaging

Embryos were imaged using a Zeiss LSM 900 confocal microscope. Plan-Apochromat 40x/1.4 N.A. oil immersion objective was used. Images were acquired with the following settings: 512 × 512 pixels, 16-bit depth, 18 z-slices separated by 0.6 μm, ~16.8 s/frame time-resolution. The fluorescence of GFP, mCherry, and eBFP2 was excited using 488-, 561-, and 405-nm lasers, respectively. Excitation power was measured and calibrated using X-Cite XR2100/XP750 Optical Power Measurement System (EXCELITAS Technologies) to keep the same experimental setting for each set of experiments. Image acquisition was started before the end of nc13 and ended after the onset of gastrulation at nc14. During imaging, data acquisition was occasionally stopped for a few seconds to correct the z-position. Obtained data were concatenated and cropped into 430 × 512 pixels (*sna* shadow enhancer), 430 × 430 pixels (*Ubx* BRE), 300 × 512 pixels (*rho* NEE), or 300 × 350 pixels (*hairy* enhancer) to remove nuclei outside of the expression domain. One hundred eighty timeframes starting from the entry into nc14 as defined by the progression of prior anaphase were used for subsequent image analysis. The temperature was kept between 22.0 to 23.0 °C during imaging.

### Airyscan super-resolution imaging

Embryos were imaged using an Airyscan2 detector equipped with a Zeiss LSM 900 confocal microscope. Plan-Apochromat 63x/1.4 N.A. oil immersion objective was used. Images were acquired with the following settings: 944 × 944 pixels (33.8 × 33.8 μm), 16-bit depth, 41 z-slices separated by 0.2 μm. For the analysis of endogenous *sna-MS2* (Supplementary Fig. 10), 780 × 780 pixels (33.3 × 33.3 μm) images were acquired. The fluorescence of GFP and mCherry was excited using 488- and 561-nm lasers, respectively. Excitation power was measured and calibrated using X-Cite XR2100/XP750 Optical Power Measurement System (EXCELITAS Technologies) to keep the same experimental setting throughout the experiments. Obtained images were processed using the “Airyscan processing” function of Zeiss ZEN software (version 3.1) in 3D with a value of 4.6 for all the channels. The temperature was kept between 22.0 to 23.0 °C during imaging.

### Quantification of TSS score of developmental enhancers

Publicly available 5´ CAGE-seq data of 2–4 h WT *Drosophila melanogaster* embryos (E-MTAB-4787)^48^ were used to quantify the level of non-coding transcription at the enhancer regions. Sequenced reads were mapped to the BDGP *Drosophila melanogaster* genome release 3 (dm3) using HISAT2 (version 2.2.0)^65^. Samtools (version 1.10) and bedtools (version 2.29.2) were used to convert files to bed format. To exclude TSS signals derived from the coding regions, reads aligned within a window of 50 bp from any exonic sequences were removed using “bedtools intersect” function. The remaining reads were integrated into a single file. Subsequently, to quantify the TSS score on enhancers, “bedtools coverage -counts” function was used to calculate the number of reads on developmental enhancers that were reliably assigned to their target genes in the previous study^30^. For quantitative comparison of the TSS score, the number of reads was normalized to the size of each enhancer.

### Analysis of TSS orientation of endogenous non-coding enhancer transcription

Initially, publicly available Zelda ChIP-seq data of nc13 and nc14 (GSM763061)^66^ were integrated to reliably determine Zelda binding sites in early embryos. Obtained Zelda binding sites were then assigned to overlapping developmental enhancers^30^ using “bedtools intersect -wa -wb” function. In total, 1062 enhancers that contain Zelda binding sites were obtained and used for further analysis. Subsequently, plus and minus TSS peaks identified by RAMPAGE analysis in *Drosophila* (GSE36212)^67^ were assigned to each Zelda binding site within a window of ±500 bp using “bedtools window” function. By integrating these datasets, enhancers with Zelda binding sites adjacent to one or more inward TSS were classified into the “inward TSS” group. Similarly, enhancers with Zelda binding sites adjacent to outward TSS were classified into the “outward TSS” group.

### Image analysis

All the image processing methods and analysis were implemented in Fiji (version 1.53q, https://fiji.sc), MATLAB (R2021b, MathWorks), and R (version 4.1.2).

### Segmentation of nuclei

Segmentation of nuclei was performed using Fiji. For each time point, the maximum projection was obtained for all z-sections per image. His2Av-eBFP2 channel was used to segment nuclei. His2Av images were first processed with a median filter to remove salty noise and a bandpass filter to remove noises below 5 pixels and above 25 pixels. Processed images were converted into binary images using Otsu´s method. Touching nuclei were then segmented with a watershed algorithm. Finally, individual nuclei were separated with a Voronoi method. The resulting binary images were manually corrected to locate MS2/PP7 transcriptional dots inside the Voronoi regions.

### Tracking of nuclei

Nuclei tracking was done using a custom MATLAB script, which finds the object with minimal movement between two consecutive timeframes. First, centroids of all the segmented nuclei were determined as the location of each nucleus for all the timeframes. For each timeframe, Euclidean distances between centroids in the current and the previous timeframe were determined. The nucleus with the minimum Euclidean distance was considered as the same lineage. When a nucleus touched the edge of the image or moved larger than the length of the nucleus, the corresponding lineage was excluded from the analysis.

### Recording of MS2 and PP7 signal

Raw images were maximum projected for each timeframe and used to record MS2 and PP7 fluorescence intensities. Using segmented regions, fluorescence intensities within each nucleus were extracted. Signals of MS2 and PP7 transcription dots were determined by taking the integral of a 5 × 5 pixels region centering the brightest pixel within a nucleus after subtracting median fluorescence intensity within a nucleus as a background. Subsequently, minimum intensities were determined for individual trajectories and subtracted to make the baseline zero.

### Detection of transcriptional bursting

First, each trajectory was smoothed by lowess function within a window of ten timeframes in R. When a nucleus had above-threshold transcription activity, the burst was considered to be started. Burst was considered to be ended when the intensity dropped below the threshold. When two connective bursts are touching each other, they were divided at the timeframe where signal intensity successively decreased from the prior two timeframes and increased in the subsequent two timeframes. When the burst duration is less than five timeframes, it was considered a false-positive. It was confirmed that this filtering method does not affect the quantification of the burst property^27^. From each nucleus, the total number of bursts was determined as the burst frequency. The cumulative fraction of active nuclei (Supplementary Fig. 3c) was determined by calculating the faction of nuclei that experienced burst production after the entry into nc14. In the analysis of *rho* NEE reporters (Supplementary Fig. 6), nuclei located in the middle of the expression domain were used.

### Quantification of burst size and the total output

Total output was determined for each nucleus by integrating signal intensities at all the time points using raw trajectory. False-colored images were generated using a segmentation mask at the 50th timeframe of the data (~14 min after the entry into nc14). They were colored with the pixel intensity proportional to the total output in corresponding nuclei. Burst size was determined by integrating signal intensities during individual bursts using raw trajectory.

### Quantification of instantaneous PP7 activity

To obtain the averaged intensity of active PP7 transcription (Supplementary Fig. 2b), intensities of the PP7 signal in individual nuclei were obtained from all the PP7 active nuclei at each time point.

### Quantification of mean MS2 and PP7 activities

Signal intensities of MS2 and PP7 were averaged from all the analyzed nuclei at each time point (Supplementary Fig. 4).

### Heatmap analysis of local enrichment of transcription factors

Segmentation of nuclei was performed as described above using Dl-GFP or Zld-GFP channel. Using a bandpass filter, binarized objects with a size between 50 to 200 pixels were obtained. Resulting segmentation images were manually corrected to locate MS2/PP7 transcriptional dots within the Voronoi regions and then used to compute a 3D voxel segment region for each individual nucleus. To quantify local Dl-GFP or Zld-GFP intensity centering the MS2/PP7 transcription site, the XYZ coordinate of the brightest MS2/PP7 pixel intensity was first determined in each 3D-segmented nucleus. Dl-GFP signal at 29 × 29 pixels (~1.0 × 1.0 μm) or Zld-GFP signal at 41 × 41 pixels (~1.4 × 1.4 μm) region centering the brightest MS2/PP7 signal was measured on the same z-plane. As a control, Dl-GFP or Zld-GFP signal centering the random XY coordinate within the segmented region on the same z-plane was measured. To analyze nuclei that reliably contain active MS2/PP7 transcription sites, nuclei with top 15% (*sna* shadow enhancer), 5% (*Ubx* BRE), or 50% (endogenous *sna*-MS2) of integrated MS2/PP7 signal were pooled from all the analyzed embryos. Then, obtained images were separated into bins based on distance from the center in a pixel increment. To determine the relative enrichment of transcription factors (Fig. 5c and Supplementary Figs. 10b, c, 11c, f, 12a, 14a), Dl-GFP or Zld-GFP intensities were then divided by the mean intensity of the circumscribed or inscribed bin area, and shown as a heatmap.

### Radial analysis of Airyscan images

For quantification of the radial profile of Dl-GFP and Zld-GFP distribution (Fig. 5d and Supplementary Figs. 12b, 14b), relative enrichment was calculated from Airyscan images as described above. Then, the mean intensity of each bin was divided by the mean of the endmost bin and then the standard error of the mean was calculated for each bin.

### Reporting summary

Further information on research design is available in the Nature Portfolio Reporting Summary linked to this article.

## Supplementary information

Supplementary Information

Description of Additional Supplementary Files

Supplementary Movie 1

Supplementary Movie 2

Supplementary Movie 3

Supplementary Movie 4

Supplementary Movie 5

Supplementary Movie 6

Supplementary Movie 7

Supplementary Movie 8

Reporting Summary

## Data Availability

The data that support this study are available from the corresponding author upon reasonable request. Previously published sequence data are available under accession numbers GSE30757, GSE86966, GSE55306, GSE36212, and E-MTAB-4787. The original live-imaging data used for the analysis shown in the main and supplementary figures are available at the Zenodo database [10.5281/zenodo.7545929]. Source data are provided with this paper.

## Source data

Source Data

## Code availability

The MATLAB codes for nuclei tracking and MS2/PP7 signal recording are available at the Zenodo database [10.5281/zenodo.7545929].

## References

[1] Levine M (2010). Transcriptional enhancers in animal development and evolution. Curr. Biol..

[2] Bartman CR, Hsu SC, Hsiung CC, Raj A, Blobel GA (2016). Enhancer regulation of transcriptional bursting parameters revealed by forced chromatin looping. Mol. Cell.

[3] Fukaya T, Lim B, Levine M (2016). Enhancer control of transcriptional bursting. Cell.

[4] Larsson AJM (2019). Genomic encoding of transcriptional burst kinetics. Nature.

[5] Kim TK (2010). Widespread transcription at neuronal activity-regulated enhancers. Nature.

[6] De Santa F (2010). A large fraction of extragenic RNA pol II transcription sites overlap enhancers. PLoS Biol..

[7] Djebali S (2012). Landscape of transcription in human cells. Nature.

[8] Core LJ (2012). Defining the status of RNA polymerase at promoters. Cell Rep..

[9] Bonn S (2012). Tissue-specific analysis of chromatin state identifies temporal signatures of enhancer activity during embryonic development. Nat. Genet..

[10] Chen RA (2013). The landscape of RNA polymerase II transcription initiation in *C. elegans* reveals promoter and enhancer architectures. Genome Res..

[11] Hirabayashi S (2019). NET-CAGE characterizes the dynamics and topology of human transcribed *cis*-regulatory elements. Nat. Genet..

[12] Kaikkonen MU (2013). Remodeling of the enhancer landscape during macrophage activation is coupled to enhancer transcription. Mol. Cell.

[13] Hah N (2011). A rapid, extensive, and transient transcriptional response to estrogen signaling in breast cancer cells. Cell.

[14] Mikhaylichenko O (2018). The degree of enhancer or promoter activity is reflected by the levels and directionality of eRNA transcription. Genes Dev..

[15] Sartorelli V, Lauberth SM (2020). Enhancer RNAs are an important regulatory layer of the epigenome. Nat. Struct. Mol. Biol..

[16] Bird AJ, Gordon M, Eide DJ, Winge DR (2006). Repression of *ADH1* and *ADH3* during zinc deficiency by Zap1-induced intergenic RNA transcripts. EMBO J..

[17] Gummalla M (2012). *abd-A* regulation by the *iab-8* noncoding RNA. PLoS Genet..

[18] Petruk S (2006). Transcription of *bxd* noncoding RNAs promoted by trithorax represses *Ubx in cis* by transcriptional interference. Cell.

[19] Martens JA, Wu PY, Winston F (2005). Regulation of an intergenic transcript controls adjacent gene transcription in *Saccharomyces cerevisiae*. Genes Dev..

[20] Martens JA, Laprade L, Winston F (2004). Intergenic transcription is required to repress the *Saccharomyces cerevisiae SER3* gene. Nature.

[21] Henninger JE (2021). RNA-mediated feedback control of transcriptional condensates. Cell.

[22] Rennie S (2018). Transcription start site analysis reveals widespread divergent transcription in *D. melanogaster* and core promoter-encoded enhancer activities. Nucleic Acids Res..

[23] Fukaya T, Lim B, Levine M (2017). Rapid rates of Pol II elongation in the *Drosophila* embryo. Curr. Biol..

[24] Hocine S, Raymond P, Zenklusen D, Chao JA, Singer RH (2013). Single-molecule analysis of gene expression using two-color RNA labeling in live yeast. Nat. Methods.

[25] Lim B, Heist T, Levine M, Fukaya T (2018). Visualization of transvection in living *Drosophila* embryos. Mol. Cell.

[26] Perry MW, Boettiger AN, Bothma JP, Levine M (2010). Shadow enhancers foster robustness of *Drosophila* gastrulation. Curr. Biol..

[27] Yokoshi M, Kawasaki K, Cambon M, Fukaya T (2022). Dynamic modulation of enhancer responsiveness by core promoter elements in living *Drosophila* embryos. Nucleic Acids Res..

[28] Groth AC, Fish M, Nusse R, Calos MP (2004). Construction of transgenic *Drosophila* by using the site-specific integrase from phage phiC31. Genetics.

[29] Ip YT, Park RE, Kosman D, Bier E, Levine M (1992). The *dorsal* gradient morphogen regulates stripes of *rhomboid* expression in the presumptive neuroectoderm of the *Drosophila* embryo. Genes Dev..

[30] Kvon EZ (2014). Genome-scale functional characterization of *Drosophila* developmental enhancers in vivo. Nature.

[31] Andersson R (2014). An atlas of active enhancers across human cell types and tissues. Nature.

[32] Henriques T (2018). Widespread transcriptional pausing and elongation control at enhancers. Genes Dev..

[33] Hsieh CL (2014). Enhancer RNAs participate in androgen receptor-driven looping that selectively enhances gene activation. Proc. Natl Acad. Sci. USA.

[34] Tsai PF (2018). A Muscle-specific enhancer RNA mediates cohesin recruitment and regulates transcription in trans. Mol. Cell.

[35] Hobson DJ, Wei W, Steinmetz LM, Svejstrup JQ (2012). RNA polymerase II collision interrupts convergent transcription. Mol. Cell.

[36] Cinghu S (2017). Intragenic enhancers attenuate host gene expression. Mol. Cell.

[37] Oudelaar AM (2019). A revised model for promoter competition based on multi-way chromatin interactions at the *α-globin* locus. Nat. Commun..

[38] Ohtsuki S, Levine M (1998). GAGA mediates the enhancer blocking activity of the *eve* promoter in the *Drosophila* embryo. Genes Dev..

[39] Choi OR, Engel JD (1988). Developmental regulation of beta-globin gene switching. Cell.

[40] Farnung L, Vos SM, Cramer P (2018). Structure of transcribing RNA polymerase II-nucleosome complex. Nat. Commun..

[41] Kujirai T (2018). Structural basis of the nucleosome transition during RNA polymerase II passage. Science.

[42] Filipovski M, Soffers JHM, Vos SM, Farnung L (2022). Structural basis of nucleosome retention during transcription elongation. Science.

[43] Mir M (2018). Dynamic multifactor hubs interact transiently with sites of active transcription in *Drosophila* embryos. Elife.

[44] Spiluttini B (2010). Splicing-independent recruitment of U1 snRNP to a transcription unit in living cells. J. Cell Sci..

[45] Yamada S (2019). The *Drosophila* pioneer factor Zelda modulates the nuclear microenvironment of a dorsal target enhancer to potentiate transcriptional output. Curr. Biol..

[46] Xiao JY, Hafner A, Boettiger AN (2021). How subtle changes in 3D structure can create large changes in transcription. Elife.

[47] Zuin J (2022). Nonlinear control of transcription through enhancer-promoter interactions. Nature.

[48] Schor IE (2017). Promoter shape varies across populations and affects promoter evolution and expression noise. Nat. Genet.

[49] Qian S, Capovilla M, Pirrotta V (1991). The *bx* region enhancer, a distant *cis*-control element of the *Drosophila Ubx* gene and its regulation by *hunchback* and other segmentation genes. EMBO J..

[50] Lewis EB (1978). A gene complex controlling segmentation in *Drosophila*.. Nature..

[51] Rogers WA, Goyal Y, Yamaya K, Shvartsman SY, Levine MS (2017). Uncoupling neurogenic gene networks in the *Drosophila* embryo. Genes Dev..

[52] Castro Alvarez JJ (2021). Repression of the Hox gene abd-A by ELAV-mediated transcriptional interference. PLoS Genet..

[53] Proudfoot N (1986). Transcriptional interference and termination between duplicated *α-globin* gene constructs suggests a novel mechanism for gene regulation. Nature.

[54] Lenstra TL, Coulon A, Chow CC, Larson DR (2015). Single-molecule imaging reveals a switch between spurious and functional ncRNA transcription. Mol. Cell.

[55] Zabidi MA (2015). Enhancer-core-promoter specificity separates developmental and housekeeping gene regulation. Nature.

[56] Azofeifa JG (2018). Enhancer RNA profiling predicts transcription factor activity. Genome Res..

[57] Fukaya T (2021). Dynamic regulation of anterior-posterior patterning genes in living *Drosophila* embryos. Curr. Biol..

[58] Yokoshi M, Segawa K, Fukaya T (2020). Visualizing the role of boundary elements in enhancer-promoter communication. Mol. Cell.

[59] Venken KJ, He Y, Hoskins RA, Bellen HJ (2006). P[acman]: a BAC transgenic platform for targeted insertion of large DNA fragments in *D. melanogaster*. Science.

[60] Bischof J, Maeda RK, Hediger M, Karch F, Basler K (2007). An optimized transgenesis system for *Drosophila* using germ-line-specific φC31 integrases. Proc. Natl Acad. Sci. USA.

[61] Ringrose L (2009). Transgenesis in *Drosophila melanogaster*. Methods Mol. Biol..

[62] Ren X (2013). Optimized gene editing technology for *Drosophila melanogaster* using germ line-specific Cas9. Proc. Natl Acad. Sci. USA.

[63] Calvo L, Ronshaugen M, Pettini T (2021). smiFISH and embryo segmentation for single-cell multi-gene RNA quantification in arthropods. Commun. Biol..

[64] Tsanov N (2016). smiFISH and FISH-quant - a flexible single RNA detection approach with super-resolution capability. Nucleic Acids Res..

[65] Kim D, Paggi JM, Park C, Bennett C, Salzberg SL (2019). Graph-based genome alignment and genotyping with HISAT2 and HISAT-genotype. Nat. Biotechnol..

[66] Harrison MM, Li XY, Kaplan T, Botchan MR, Eisen MB (2011). Zelda binding in the early *Drosophila melanogaster* embryo marks regions subsequently activated at the maternal-to-zygotic transition. PLoS Genet..

[67] Batut P, Dobin A, Plessy C, Carninci P, Gingeras TR (2013). High-fidelity promoter profiling reveals widespread alternative promoter usage and transposon-driven developmental gene expression. Genome Res..

[68] Hannon CE, Blythe SA, Wieschaus EF (2017). Concentration dependent chromatin states induced by the bicoid morphogen gradient. Elife.

