# Supplementary material for: Dynamic interplay between non-coding enhancer transcription and gene activity in development
Source: Nat Commun. 2023 Feb 20;14:826. doi: 10.1038/s41467-023-36485-1 (PMC9941499; doi:10.1038/s41467-023-36485-1)
Supplement: Supplementary file 1 — Supplementary Information [file 41467_2023_36485_MOESM1_ESM.pdf]

Supplementary Information

Dynamic interplay between non-coding enhancer transcription and gene activity in development

Kota Hamamoto[1,2], Yusuke Umemura[1,2], Shiho Makino[1], Takashi Fukaya[1,2*]

[1] Laboratory of Transcription Dynamics, Research Center for Biological Visualization, Institute for Quantitative Biosciences, The University of Tokyo, Bunkyo-ku, Tokyo, Japan
[2]Department of Life Sciences, Graduate School of Arts and Sciences, The University of Tokyo, Bunkyo-ku, Tokyo, Japan

* To whom correspondence should be addressed

Email: tfukaya@iqb.u-tokyo.ac.jp

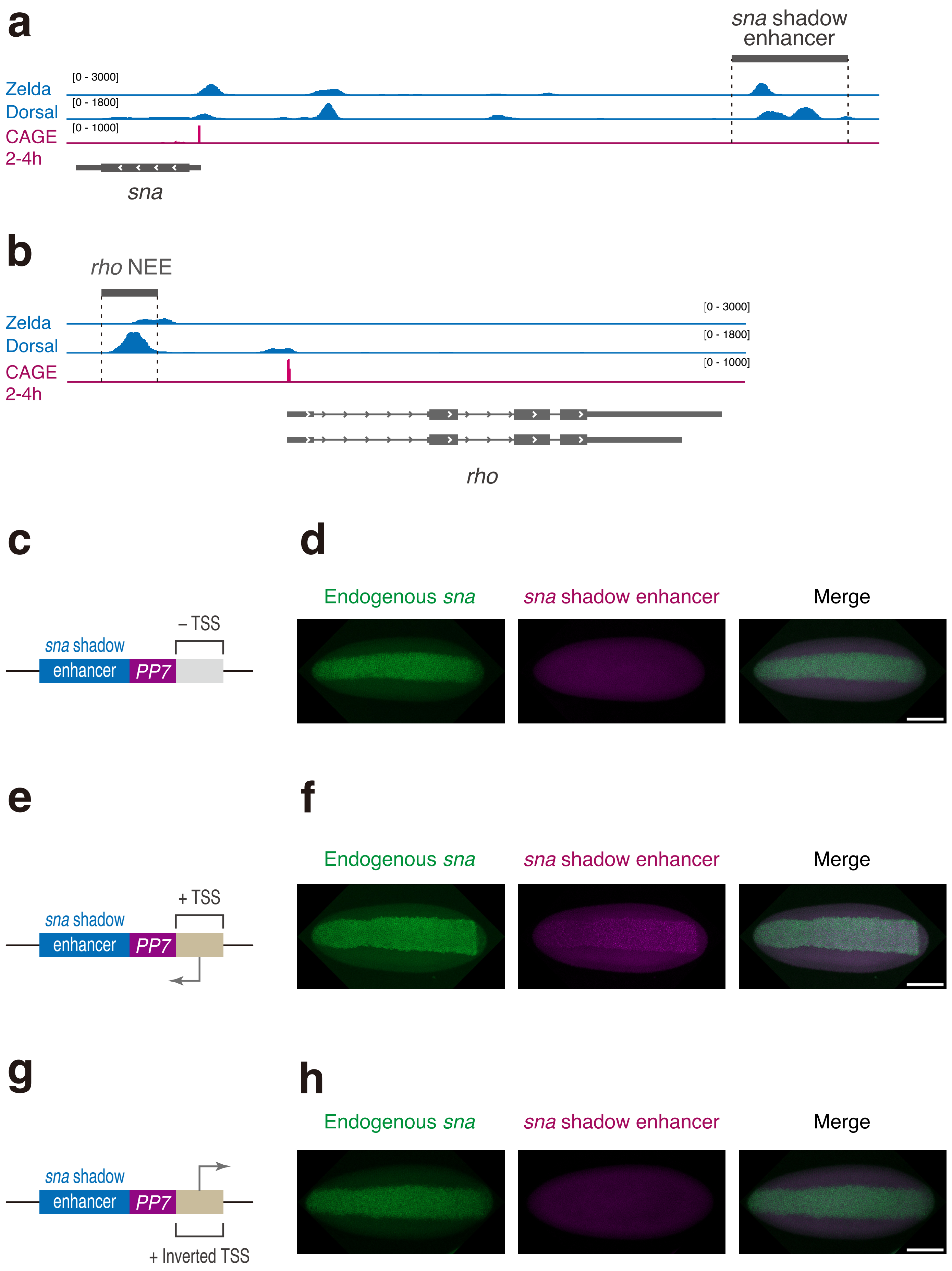
a
sna shadow enhancer
Zelda
[0 - 3000]
Dorsal
[0 - 1800]
CAGE 2-4h
[0 - 1000]
sna
b
rho NEE
Zelda
[0 - 3000]
Dorsal
[0 - 1800]
CAGE 2-4h
[0 - 1000]
rho
c
– TSS
sna shadow enhancer
PP7
d
Endogenous sna
sna shadow enhancer
Merge
e
+ TSS
sna shadow enhancer
PP7
f
Endogenous sna
sna shadow enhancer
Merge
g
sna shadow enhancer
PP7
+ Inverted TSS
h
Endogenous sna
sna shadow enhancer
Merge

Supplementary Fig. 1. Induction of unidirectional non-coding enhancer transcription at the synthetic locus.

(a) Organization of the endogenous *sna* locus. Zelda ChIP-seq data from nc13 WT embryos (GSM763061) [1], Dorsal ChIP-seq from 2- to 4-h WT embryos (GSM1341814) [2], CAGE-seq data from 2- to 4-h WT embryos (ERR1425056) [3] were visualized with Integrative Genomics Viewer.

(b) Organization of the endogenous *rho* locus. Zelda ChIP-seq data from nc13 WT embryos (GSM763061) [1], Dorsal ChIP-seq from 2- to 4-h WT embryos (GSM1341814) [2], CAGE-seq data from 2- to 4-h WT embryos (ERR1425056) [3] were visualized with Integrative Genomics Viewer.

(c) Schematic representation of the PP7-enhancer cassette without TSS.

(d) Fluorescent *in situ* hybridization using probes against endogenous *sna* gene (left) and *sna* shadow enhancer (middle). nc14 embryos containing the PP7-enhancer cassette without TSS were analyzed. The image is oriented with anterior to the left and ventral view facing up. Scale bar indicates 100 μm.

(e) Minimal core promoter motifs were placed adjacent to the enhancer to drive non-coding enhancer transcription.

(f) Fluorescent *in situ* hybridization using probes against endogenous *sna* gene (left) and *sna* shadow enhancer (middle). nc14 embryos containing the PP7-enhancer cassette fused with promoter motifs were analyzed. The image is oriented with anterior to the left and ventral view facing up. Scale bar indicates 100 μm.

(g) Inverted promoter motifs were fused with the enhancer.

(h) Fluorescent *in situ* hybridization of embryos using probes against endogenous *sna* gene (left) and *sna* shadow enhancer (middle). nc14 embryos containing the PP7-enhancer cassette fused with inverted promoter motifs were analyzed. The image is oriented with anterior to the left and ventral view facing up. Scale bar indicates 100 μm.

Supplementary Fig. 2

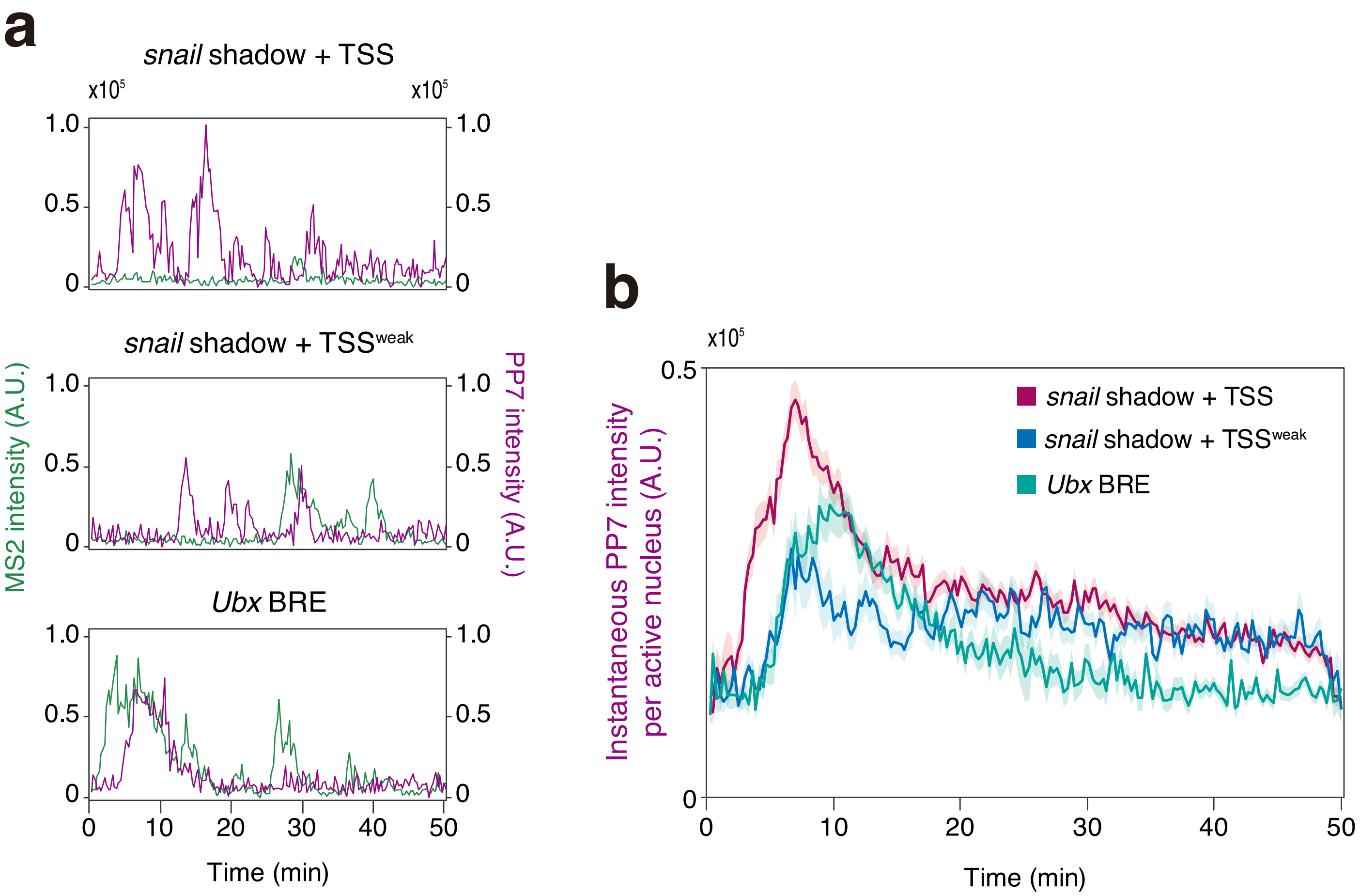

Supplementary Fig. 2. Level of non-coding enhancer transcription at the synthetic locus.

(a) Representative trajectories of transcription activities of the reporter locus containing *sna* shadow enhancer + TSS (top), + TSS^{weak} (Inr mutant; middle), or *Ubx* BRE (bottom).

(b) Instantaneous signal intensity in PP7 active nuclei. A total of 237, 236, and 226 nuclei were analyzed from a single embryo for the reporter locus containing *sna* shadow enhancer + TSS, + TSS^{weak}, or *Ubx* BRE, respectively.

A.U.; arbitrary unit. Shades represent the mean ± standard error of the mean.

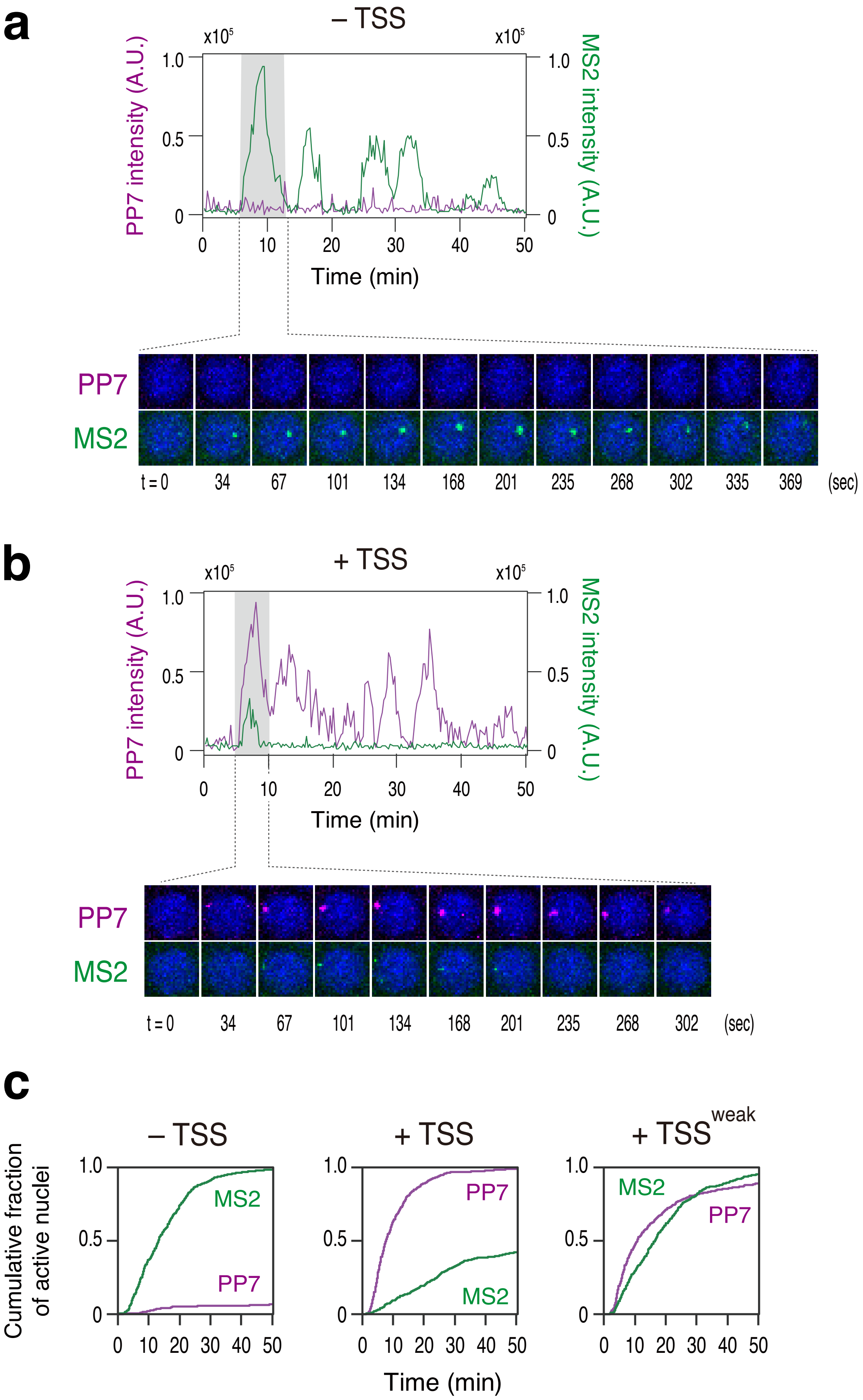
a
– TSS
x10^5
x10^5
PP7 intensity (A.U.)
MS2 intensity (A.U.)
1.0
0.5
0
0 10 20 30 40 50
Time (min)
PP7
MS2
t = 0 34 67 101 134 168 201 235 268 302 335 369 (sec)
b
+ TSS
x10^5
x10^5
PP7 intensity (A.U.)
MS2 intensity (A.U.)
1.0
0.5
0
0 10 20 30 40 50
Time (min)
PP7
MS2
t = 0 34 67 101 134 168 201 235 268 302 (sec)
c
Cumulative fraction of active nuclei
– TSS
+ TSS
+ TSS weak
MS2
PP7
1.0
0.5
0
0 10 20 30 40 50
Time (min)

Supplementary Fig. 3. Activation kinetics of enhancer and target gene transcription.

(a) Single-nucleus snapshots of MS2 and PP7 channels during transcription bursting. MS2/PP7 trajectory is the same as the one shown in Fig. 1b (– TSS).

(b) Single-nucleus snapshots of MS2 and PP7 channels during transcription bursting. MS2/PP7 trajectory is the same as the one shown in Fig. 1b (+ TSS).

(c) Cumulative fraction of actively transcribing nuclei. A total of 676, 719, and 700 ventral-most nuclei, respectively, were analyzed from three independent embryos for the reporter locus containing – TSS (left), + TSS (middle), or + TSS^{weak} (right) at the enhancer region.

A.U.; arbitrary unit.

Supplementary Fig. 4

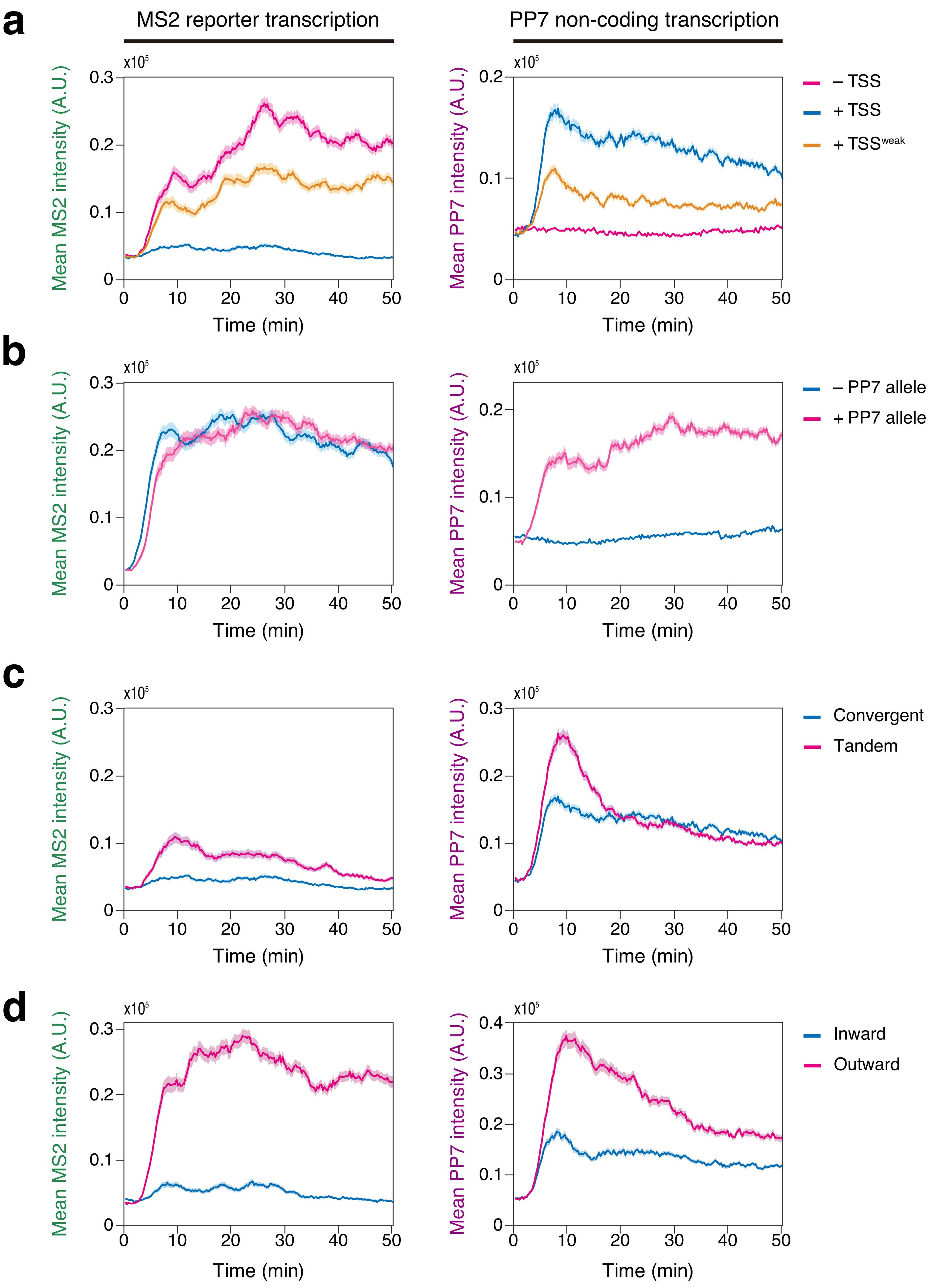

Supplementary Fig. 4. Mean MS2 and PP7 activities in a series of synthetic reporter loci.

(a) Mean MS2 (left) and PP7 (right) intensities were calculated using all the trajectories from three independent embryos for the reporter locus containing – TSS, + TSS, or + TSS^{weak} at the enhancer region (Fig. 1).

(b) Mean MS2 (left) and PP7 (right) intensities were calculated using all the trajectories from three independent embryos with or without the PP7 allele on the other homologous chromosome (Fig. 2).

(c) Mean MS2 (left) and PP7 (right) intensities were calculated using all the trajectories from three independent embryos for the reporter locus driving non-coding enhancer transcription in a convergent or a tandem orientation (Fig. 3). Plots of Convergent are the same as the plots of + TSS shown in (a).

(d) Mean MS2 (left) and PP7 (right) intensities were calculated using all the trajectories from three independent embryos for the reporter locus driving PP7 transcription in an inward or an outward orientation (Fig. 4).

A.U.; arbitrary unit. Shades represent the mean ± standard error of the mean.

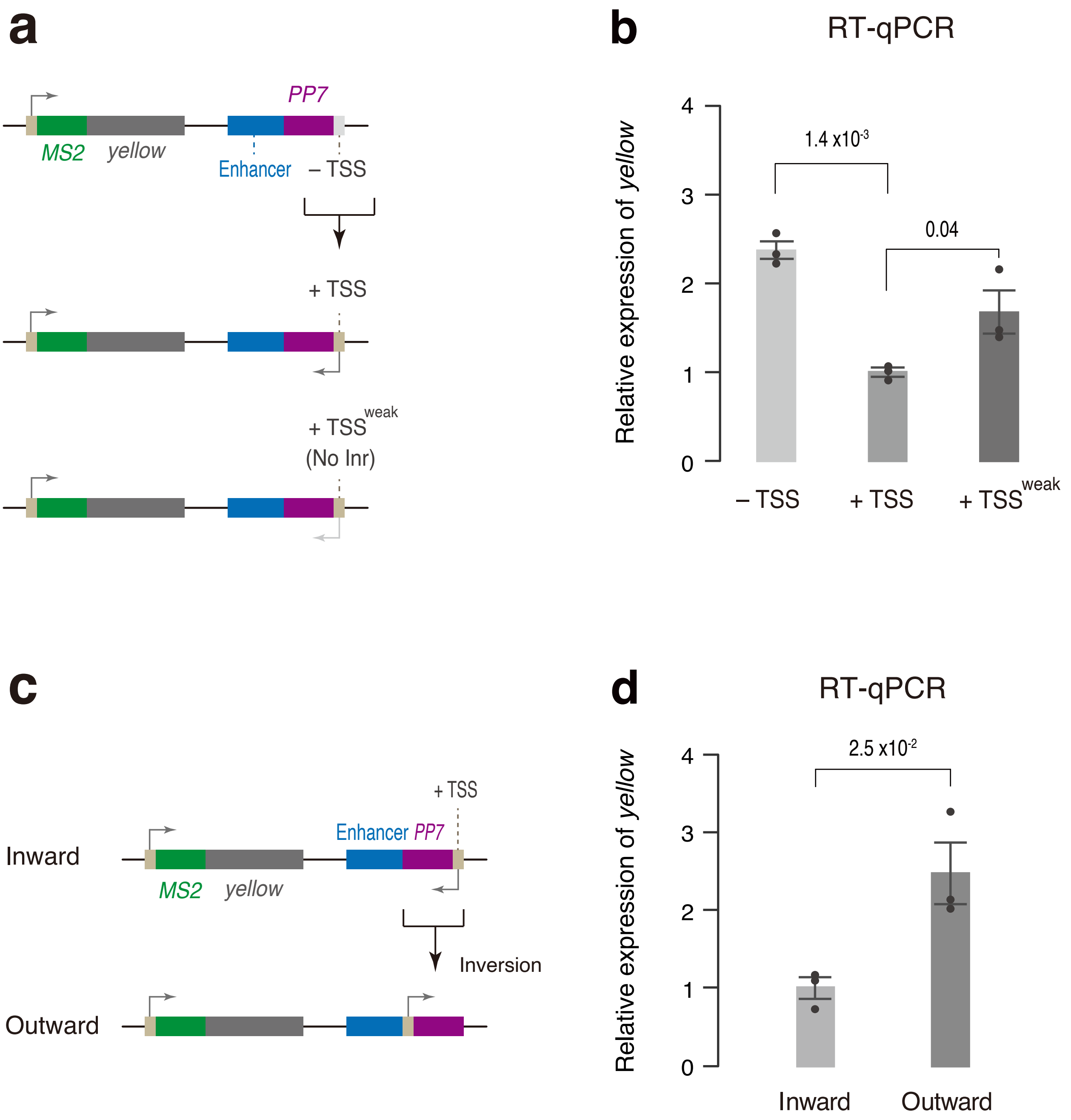
a
PP7
MS2
yellow
Enhancer
– TSS
+ TSS
+ TSS weak
(No Inr)
b
RT-qPCR
Relative expression of yellow
1.4 x10-3
0.04
– TSS
+ TSS
+ TSS weak
c
+ TSS
Enhancer PP7
Inward
MS2
yellow
Inversion
Outward
d
RT-qPCR
Relative expression of yellow
2.5 x10-2
Inward
Outward

Supplementary Fig. 5. RT-qPCR analysis of *MS2-yellow* reporter expression.

(a) Synthetic reporter loci used in the analysis shown in (b).

(b) Relative mRNA level of *yellow* reporter gene in nc14 embryos was measured by RT-qPCR. Expression levels were normalized to *rp49*. Three biological replicates were taken for each reporter construct. P-values were calculated by two-sided Dunnett's test.

(c) Synthetic reporter loci used in the analysis shown in (d).

(d) Relative mRNA level of *yellow* reporter gene in nc14 embryos was measured by RT-qPCR. Expression levels were normalized to *rp49*. Three biological replicates were taken for each reporter construct. P-value was calculated by two-sided, unpaired Student's t-test.

Error bars represent the mean ± standard error of the mean. Source data are provided as a Source Data file.

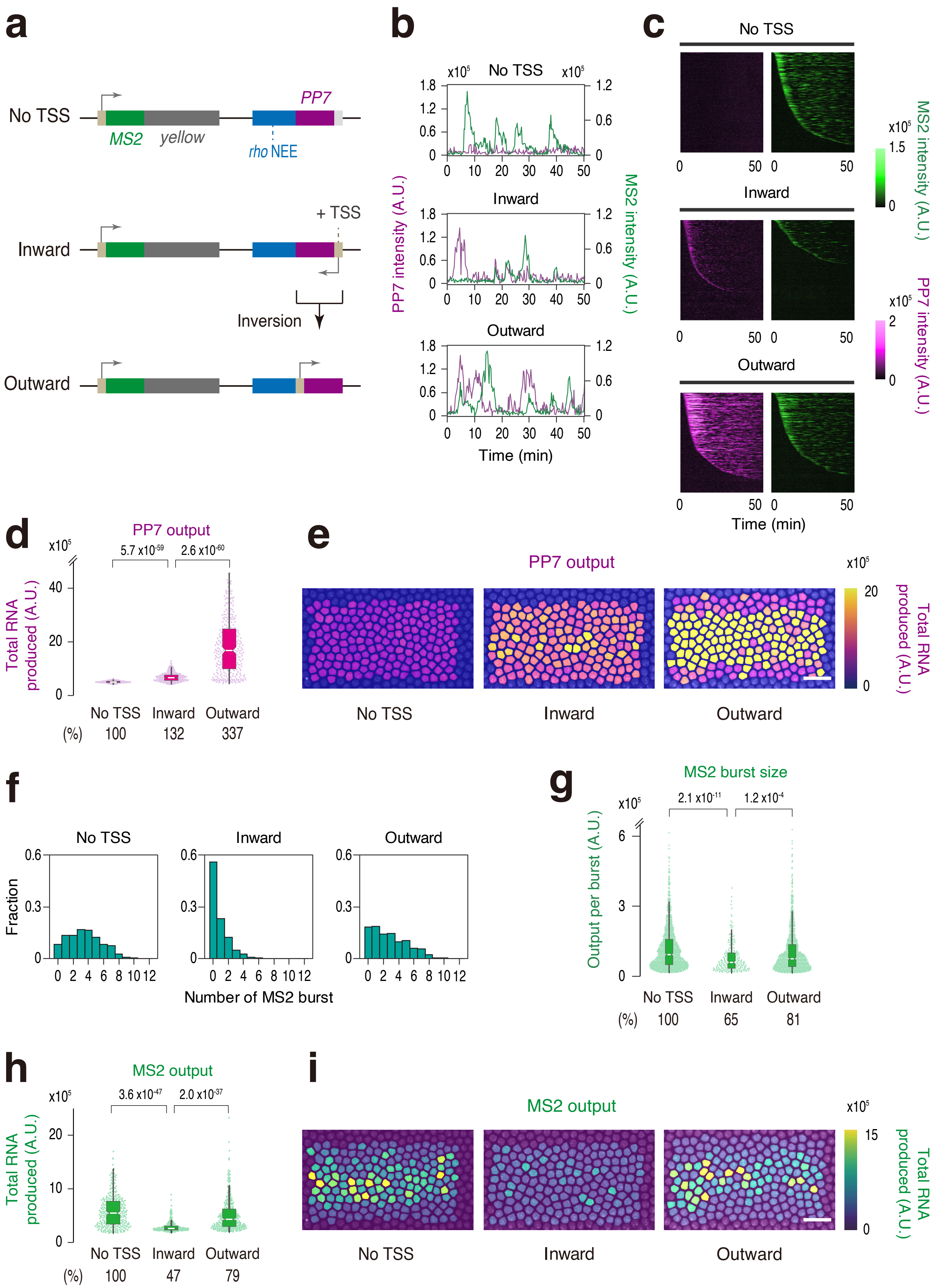
a
No TSS
MS2
yellow
rho NEE
PP7
Inward
+ TSS
Inversion
Outward
b
No TSS
PP7 intensity (A.U.)
MS2 intensity (A.U.)
Inward
Outward
Time (min)
c
No TSS
Inward
Outward
Time (min)
MS2 intensity (A.U.)
PP7 intensity (A.U.)
d
PP7 output
5.7 x10-59
2.6 x10-60
Total RNA produced (A.U.)
No TSS
Inward
Outward
(%)
100
132
337
e
PP7 output
Total RNA produced (A.U.)
No TSS
Inward
Outward
f
No TSS
Inward
Outward
Fraction
Number of MS2 burst
g
MS2 burst size
2.1 x10-11
1.2 x10-4
Output per burst (A.U.)
No TSS
Inward
Outward
(%)
100
65
81
h
MS2 output
3.6 x10-47
2.0 x10-37
Total RNA produced (A.U.)
No TSS
Inward
Outward
(%)
100
47
79
i
MS2 output
Total RNA produced (A.U.)
No TSS
Inward
Outward

Supplementary Fig. 6. Non-coding transcription of *rho* NEE attenuates target gene transcription.

(a) The *MS2-yellow* reporter gene was placed under the control of the *rho* NEE fused with 24x PP7 repeats (top). Minimal core promoter motifs were placed adjacent to the enhancer to drive non-coding transcription in an inward orientation (middle). PP7 transcription unit was inverted to drive non-coding transcription in an outward orientation (bottom).

(b) Representative trajectories of transcriptional activities of the reporter locus with No TSS (top), inward PP7 transcription (middle), or outward PP7 transcription (bottom).

(c) MS2 and PP7 trajectories for all the analyzed nuclei. Each row represents the MS2 or PP7 trajectory for a single nucleus. A total of 331, 313, and 345 nuclei, respectively, were analyzed from three independent embryos for the reporter locus with No TSS (top), inward PP7 transcription (middle), or outward PP7 transcription (bottom). Nuclei were ordered by the onset of MS2 or PP7 transcription in nc14, separately. The same number of nuclei were analyzed hereafter.

(d) Boxplot showing the distribution of total output of PP7 transcription. The box indicates the lower (25%) and upper (75%) quantile and the white line indicates the median. Whiskers extend to the most extreme, non-outlier data points. The double hash mark on the y-axis indicates that > 99% of the data points are presented.

(e) Each nucleus was colored with respect to the total output of PP7 transcription in the representative embryos. The maximum projected image of His2Av-eBFP2 is shown in gray. The image is oriented with anterior to the left. Scale bar indicates 20 μm.

(f) Histograms showing the distribution of MS2 burst frequency.

(g) Boxplot showing the distribution of MS2 burst size. The box indicates the lower (25%) and upper (75%) quantile and the white line indicates the median. Whiskers extend to the most extreme, non-outlier data points. A total of 1159, 243, and 964 MS2 bursts,

respectively, were analyzed for the reporter locus with No TSS, inward PP7 transcription, or outward PP7 transcription. The double hash mark on the y-axis indicates that >99% of the data points are presented.

(h) Boxplot showing the distribution of total output of MS2 transcription. The box indicates the lower (25%) and upper (75%) quantile and the white line indicates the median. Whiskers extend to the most extreme, non-outlier data points.

(i) Each nucleus was colored with respect to the total output of MS2 transcription in the representative embryos. The maximum projected image of His2Av-eBFP2 is shown in gray. The image is oriented with anterior to the left. Scale bar indicates 20 μm.

A.U.; arbitrary unit. Percentages shown at the bottom of boxplots represent the relative values of median. P-values were calculated by two-sided Wilcoxon rank sum test with Bonferroni correction. Source data are provided as a Source Data file.

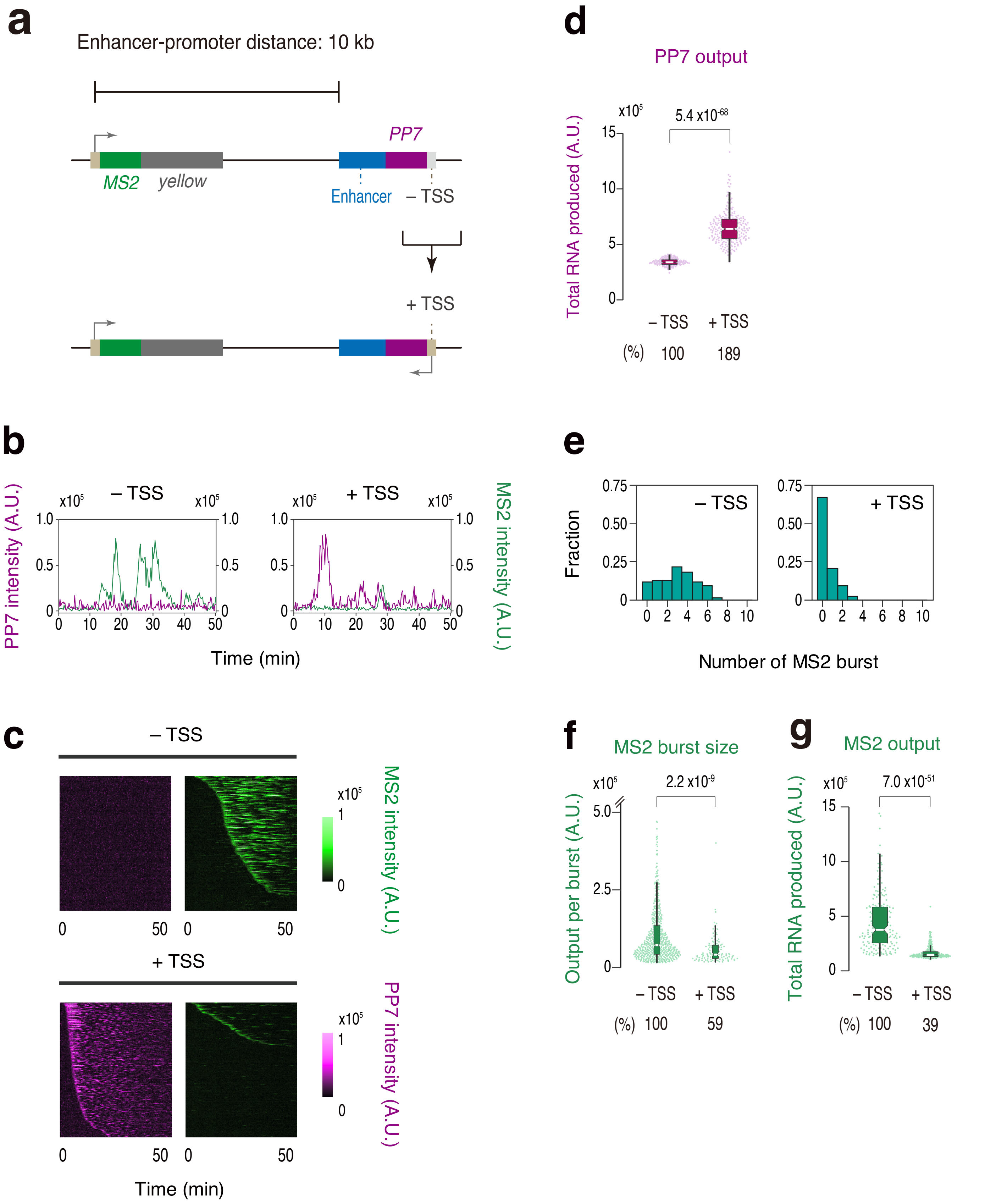
a
Enhancer-promoter distance: 10 kb
PP7
MS2
yellow
Enhancer
– TSS
+ TSS
b
PP7 intensity (A.U.)
– TSS
+ TSS
MS2 intensity (A.U.)
Time (min)
c
– TSS
+ TSS
MS2 intensity (A.U.)
PP7 intensity (A.U.)
Time (min)
d
PP7 output
Total RNA produced (A.U.)
5.4 x10-68
(%) 100 189
e
Fraction
Number of MS2 burst
f
MS2 burst size
Output per burst (A.U.)
2.2 x10-9
(%) 100 59
g
MS2 output
Total RNA produced (A.U.)
7.0 x10-51
(%) 100 39

Supplementary Fig. 7. Impacts of enhancer-promoter distance on inhibitory effects of non-coding transcription.

(a) Unrelated spacer sequence was inserted between the *MS2-yellow* reporter and *sna* shadow enhancer to extend the enhancer-promoter distance to 10 kb.

(b) Representative trajectories of transcription activities of the reporter locus containing – TSS (left) or + TSS (right) at the enhancer region.

(c) MS2 and PP7 trajectories for all the analyzed nuclei. Each row represents the MS2 or PP7 trajectory for a single nucleus. A total of 180 and 237 ventral-most nuclei, respectively, were analyzed from a single embryo for the reporter locus containing – TSS (top) or + TSS (bottom) at the enhancer region. Nuclei were ordered by the onset of MS2 or PP7 transcription in nc14, separately. The same number of nuclei were analyzed hereafter.

(d) Boxplot showing the distribution of total output of PP7 transcription. The box indicates the lower (25%) and upper (75%) quantile and the white line indicates the median. Whiskers extend to the most extreme, non-outlier data points.

(e) Histograms showing the distribution of MS2 burst frequency.

(f) Boxplot showing the distribution of MS2 burst size. The box indicates the lower (25%) and upper (75%) quantile and the white line indicates the median. Whiskers extend to the most extreme, non-outlier data points. A total of 546 and 111 MS2 bursts, respectively, were analyzed for the reporter locus containing – TSS or + TSS at the enhancer region. The double hash mark on the y-axis indicates that >99% of the data points are presented.

(g) Boxplot showing the distribution of total output of MS2 transcription. The box indicates the lower (25%) and upper (75%) quantile and the white line indicates the median. Whiskers extend to the most extreme, non-outlier data points.

A.U.; arbitrary unit. Percentages shown at the bottom of boxplots represent the relative values of median. P-values were calculated by two-sided Wilcoxon rank sum test. Source

data are provided as a Source Data file.

Supplementary Fig. 8

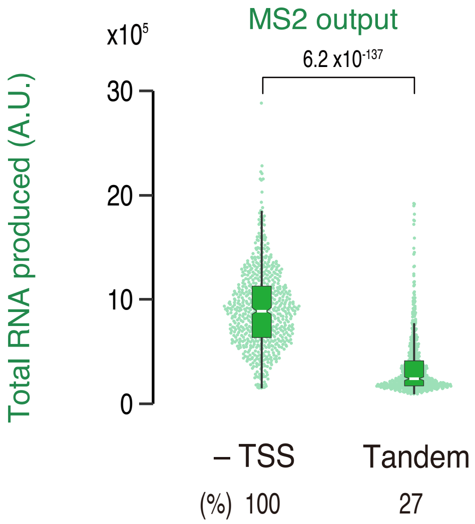

Supplementary Fig. 8. Inhibitory effects of non-coding enhancer transcription in a tandem reporter locus.

Boxplot showing the distribution of total output of MS2 transcription. A total of 676 and 638 ventral-most nuclei, respectively, were analyzed from three independent embryos for the reporter locus lacking intergenic TSS or driving non-coding enhancer transcription in a tandem orientation. The box indicates the lower (25%) and upper (75%) quantile and the white line indicates the median. Whiskers extend to the most extreme, non-outlier data points. Plot of – TSS is the same as the plot shown in Fig. 1h. Plot of Tandem is the same as the plot shown in Fig. 3g.

Percentages shown at the bottom of boxplot represent the relative values of median. P-value was calculated by two-sided Wilcoxon rank sum test. Source data are provided as a Source Data file.

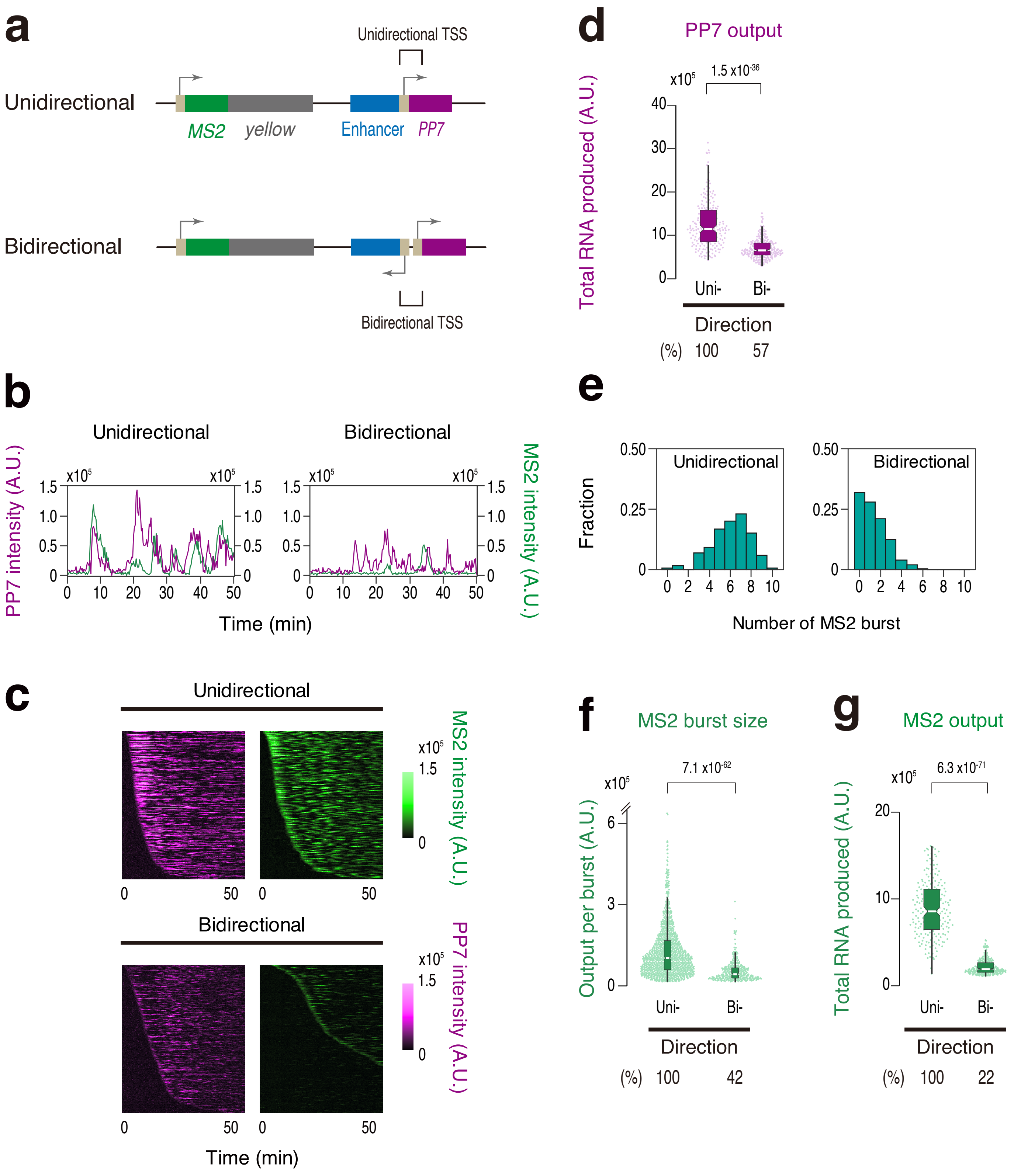
a
Unidirectional TSS
Unidirectional
MS2
yellow
Enhancer
PP7
Bidirectional
Bidirectional TSS
b
Unidirectional
Bidirectional
PP7 intensity (A.U.)
MS2 intensity (A.U.)
x10^5
Time (min)
c
Unidirectional
Bidirectional
MS2 intensity (A.U.)
PP7 intensity (A.U.)
Time (min)
d
PP7 output
1.5 x10^-36
Total RNA produced (A.U.)
Uni-
Bi-
Direction
(%) 100 57
e
Fraction
Unidirectional
Bidirectional
Number of MS2 burst
f
MS2 burst size
7.1 x10^-62
Output per burst (A.U.)
Uni-
Bi-
Direction
(%) 100 42
g
MS2 output
6.3 x10^-71
Total RNA produced (A.U.)
Uni-
Bi-
Direction
(%) 100 22

Supplementary Fig. 9. Bidirectional non-coding transcription attenuates target gene expression.

(a) Unidirectional TSS was converted into bidirectional TSS by placing additional minimal core promoter motifs.

(b) Representative trajectories of transcription activities of the reporter locus driving unidirectional (left) or bidirectional (right) non-coding transcription.

(c) MS2 and PP7 trajectories for all the analyzed nuclei. Each row represents the MS2 or PP7 trajectory for a single nucleus. A total of 198 and 254 ventral-most nuclei, respectively, were analyzed from a single embryo for the reporter locus driving unidirectional (top) or bidirectional (bottom) PP7 transcription. Nuclei were ordered by the onset of MS2 or PP7 transcription in nc14, separately. The same number of nuclei were analyzed hereafter.

(d) Boxplot showing the distribution of total output of PP7 transcription. The box indicates the lower (25%) and upper (75%) quantile and the white line indicates the median. Whiskers extend to the most extreme, non-outlier data points.

(e) Histograms showing the distribution of MS2 burst frequency.

(f) Boxplot showing the distribution of MS2 burst size. The box indicates the lower (25%) and upper (75%) quantile and the white line indicates the median. Whiskers extend to the most extreme, non-outlier data points. A total of 1202 and 346 MS2 bursts, respectively, were analyzed for the reporter locus driving unidirectional or bidirectional non-coding transcription. The double hash mark on the y-axis indicates that >99% of the data points are presented.

(g) Boxplot showing the distribution of total output of MS2 transcription. The box indicates the lower (25%) and upper (75%) quantile and the white line indicates the median. Whiskers extend to the most extreme, non-outlier data points.

A.U.; arbitrary unit. Percentages shown at the bottom of boxplots represent the relative

values of median. P-values were calculated by two-sided Wilcoxon rank sum test. Source data are provided as a Source Data file.

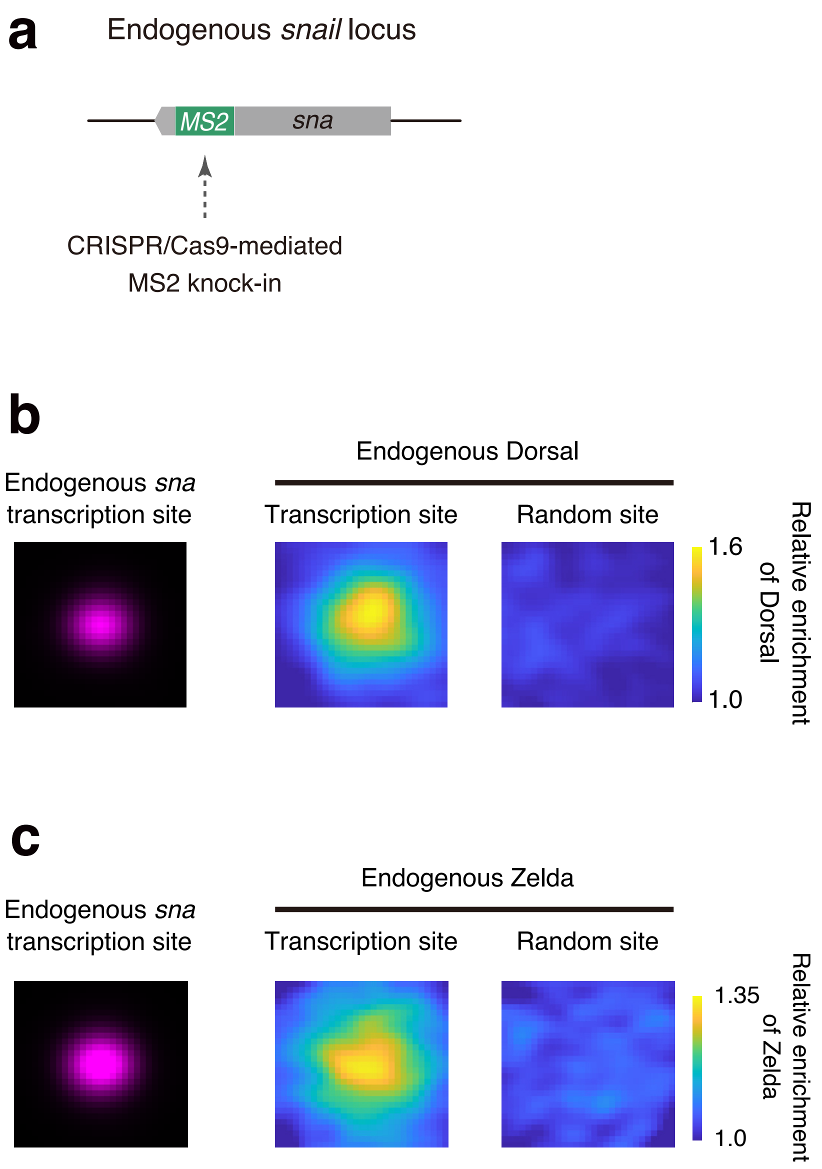
a
Endogenous *snail* locus
MS2
sna
CRISPR/Cas9-mediated
MS2 knock-in
b
Endogenous Dorsal
Endogenous *sna*
transcription site
Transcription site
Random site
1.6
1.0
Relative enrichment
of Dorsal
c
Endogenous Zelda
Endogenous *sna*
transcription site
Transcription site
Random site
1.35
1.0
Relative enrichment
of Zelda

Supplementary Fig. 10. Formation of Dl and Zld hub at the endogenous *sna* locus.

(a) 24x MS2 repeats were inserted into the 3′ UTR of endogenous *sna* gene.

(b) Heatmaps showing the averaged *sna-MS2* intensity (left) and averaged distribution of Dl-GFP centering the MS2 transcription site (center) or random site (right). A total of 1125 MS2-transcribing nuclei obtained from 20 independent embryos were analyzed.

(c) Heatmaps showing the averaged *sna-MS2* intensity (left) and averaged distribution of Zld-GFP centering the MS2 transcription site (center) or random site (right). A total of 1207 MS2-transcribing nuclei obtained from 20 independent embryos were analyzed.

Supplementary Fig. 11

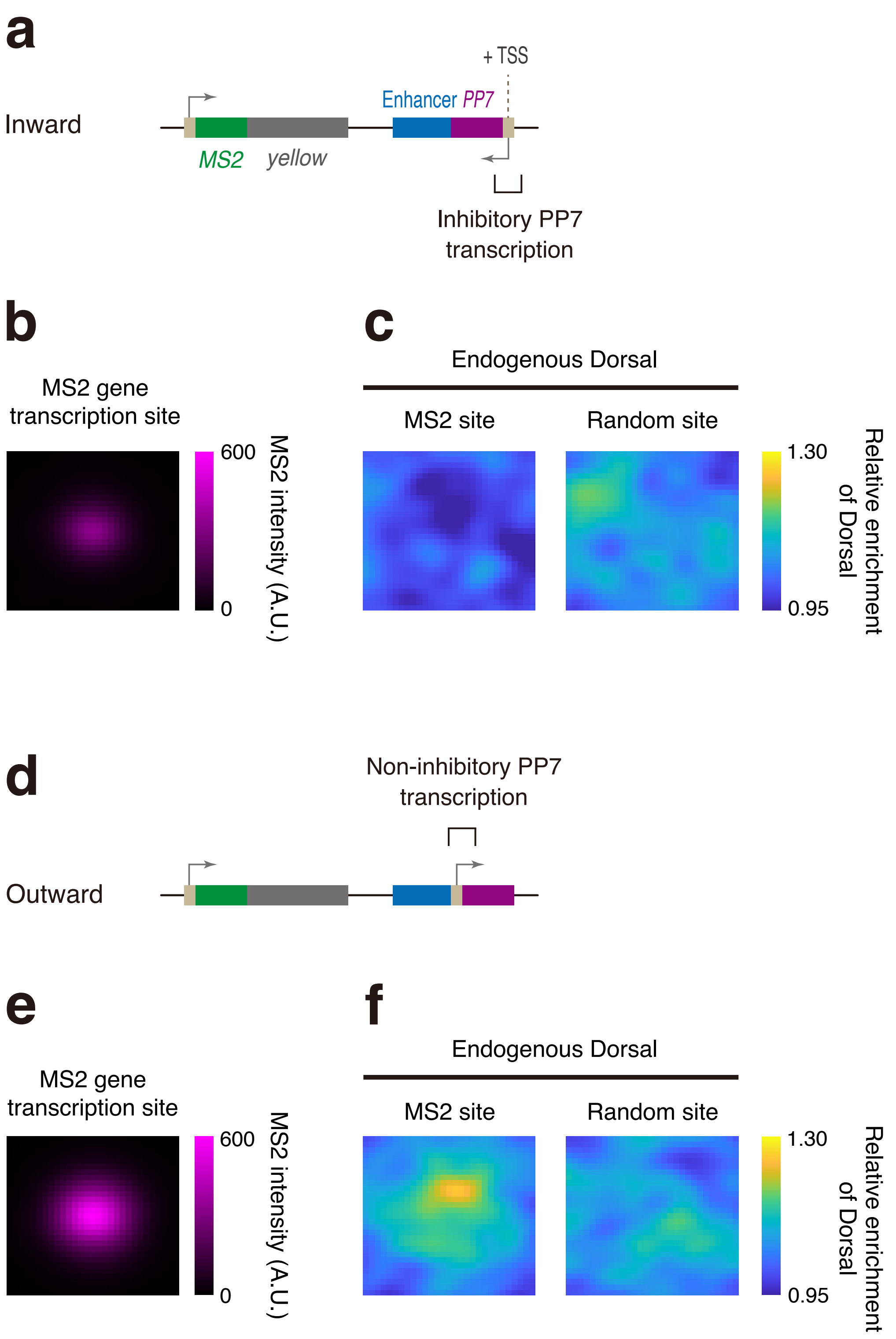

Supplementary Fig. 11. Assembly of Dl hub at the MS2 transcription site.

(a) Synthetic reporter locus used in the analysis of (b) and (c).

(b) Heatmap showing the averaged MS2 intensity.

(c) Heatmaps showing the averaged distribution of Dl-GFP centering the MS2 transcription site (left) or random site (right). A total of 175 MS2-transcribing nuclei obtained from 10 independent embryos were used for the analysis.

(d) Synthetic reporter used in the analysis of (e) and (f).

(e) Heatmap showing the averaged MS2 intensity.

(f) Heatmaps showing the averaged distribution of Dl-GFP centering the MS2 transcription site (left) or random site (right). A total of 165 MS2-transcribing nuclei obtained from 10 independent embryos were used for the analysis.

Supplementary Fig. 12

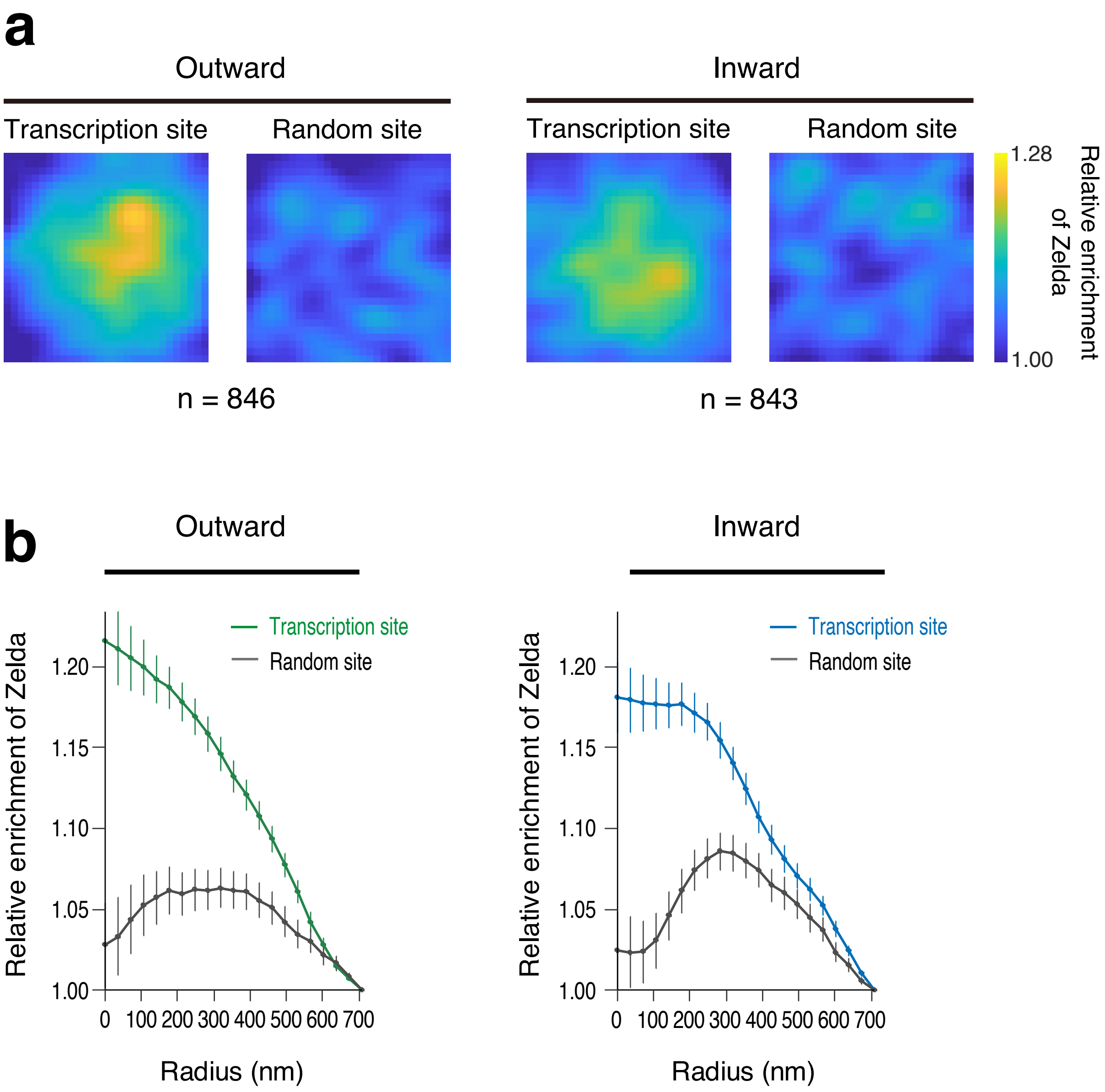

Supplementary Fig. 12. Impacts of enhancer self-transcription on the local concentration of Zld.

(a) Heatmaps showing the averaged distribution of Zld-GFP centering the PP7 transcription site or random site. A total of 846 and 843 PP7-transcribing nuclei, respectively, were obtained from 50 independent embryos for the reporter locus driving PP7 transcription in an outward (left) or an inward orientation (right).

(b) Radial profiles of the averaged Zld-GFP distribution shown in (a). Error bars represent standard error of the mean. Difference between the areas of distribution curves of transcription site was tested by two-sided Wilcoxon rank sum test ($p = 0.42$).

Source data are provided as a Source Data file.

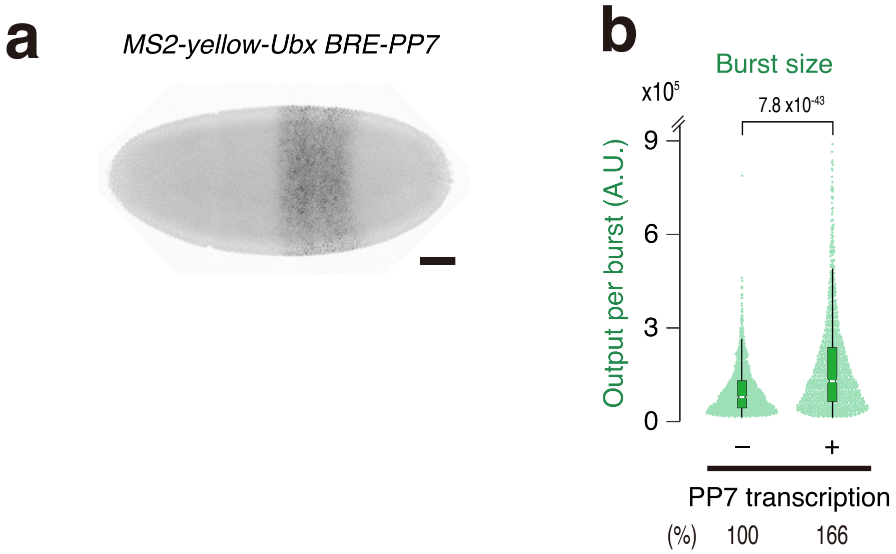
a
MS2-yellow-Ubx BRE-PP7
b
Burst size
x10^5
7.8 x10^-43
9
6
3
0
Output per burst (A.U.)
–
+
PP7 transcription
(%)
100
166

Supplementary Fig. 13. Non-coding BRE transcription correlates with the efficiency of burst induction.

(a) Single-molecule inexpensive FISH using probes against *yellow* gene. nc14 embryos containing *Ubx* BRE (Fig. 6c; top) were analyzed. The image is oriented with anterior to the left. Scale bar indicates 50 μm.

(b) Boxplot showing the distribution of MS2 burst size. The box indicates the lower (25%) and upper (75%) quantile and the white line indicates the median. Whiskers extend to the most extreme, non-outlier data points. A total of 1195 and 1278 MS2 bursts, respectively, were analyzed from nuclei grouped by the absence or presence of PP7 transcription from unmodified BRE (Fig. 6c; top). The double hash mark on the y-axis indicates that >99% of the data points are presented.

A.U.; arbitrary unit. Percentages shown at the bottom of boxplot represent the relative values of median. P-value was calculated by two-sided Wilcoxon rank sum test. Source data are provided as a Source Data file.

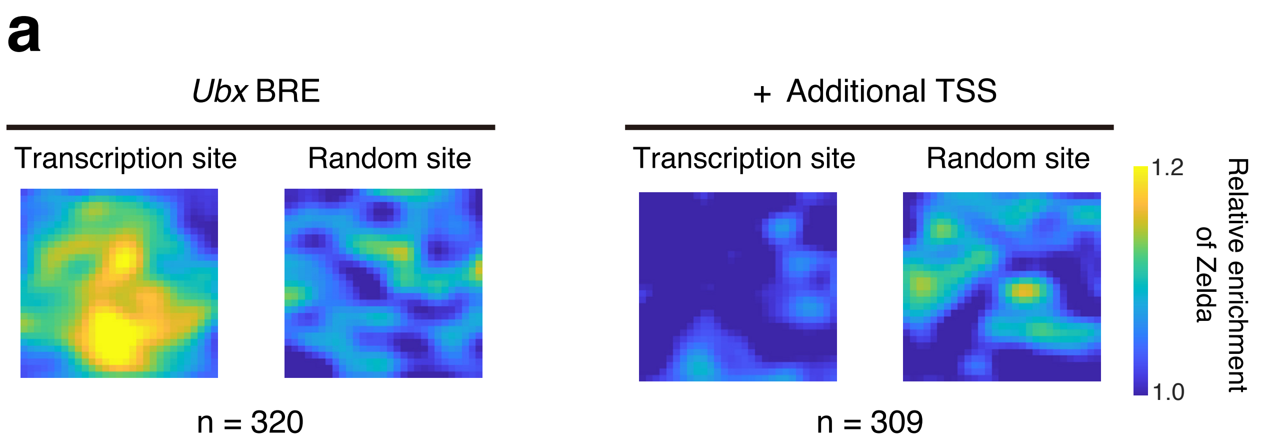
a
Ubx BRE
+ Additional TSS
Transcription site
Random site
Transcription site
Random site
1.2
1.0
Relative enrichment of Zelda
n = 320
n = 309

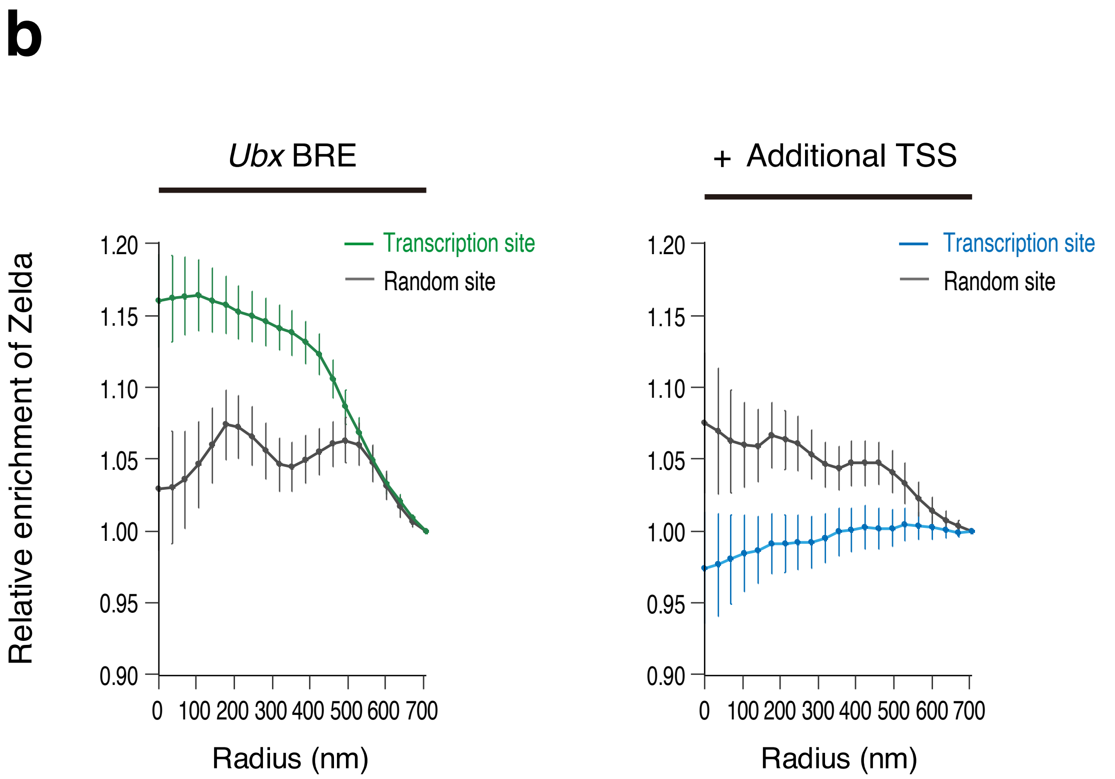
b
Ubx BRE
+ Additional TSS
Relative enrichment of Zelda
Transcription site
Random site
Transcription site
Random site
1.20
1.15
1.10
1.05
1.00
0.95
0.90
0 100 200 300 400 500 600 700
Radius (nm)
Radius (nm)

Supplementary Fig. 14. Assembly of Zld hub at the *Ubx* BRE reporter locus.

(a) Heatmaps showing the averaged distribution of Zld-GFP centering the MS2 transcription site or random site. A total of 320 and 309 MS2-transcribing nuclei, respectively, were obtained from 50 independent embryos for the reporter locus without (left, Fig. 6c; middle) or with additional TSS (right, Fig. 6c; bottom).

(b) Radial profiles of the averaged Zld-GFP distribution shown in (a). Error bars represent standard error of the mean. Difference between the areas of distribution curves of transcription site was tested by two-sided Wilcoxon rank sum test ($p = 0.01$).

Source data are provided as a Source Data file.

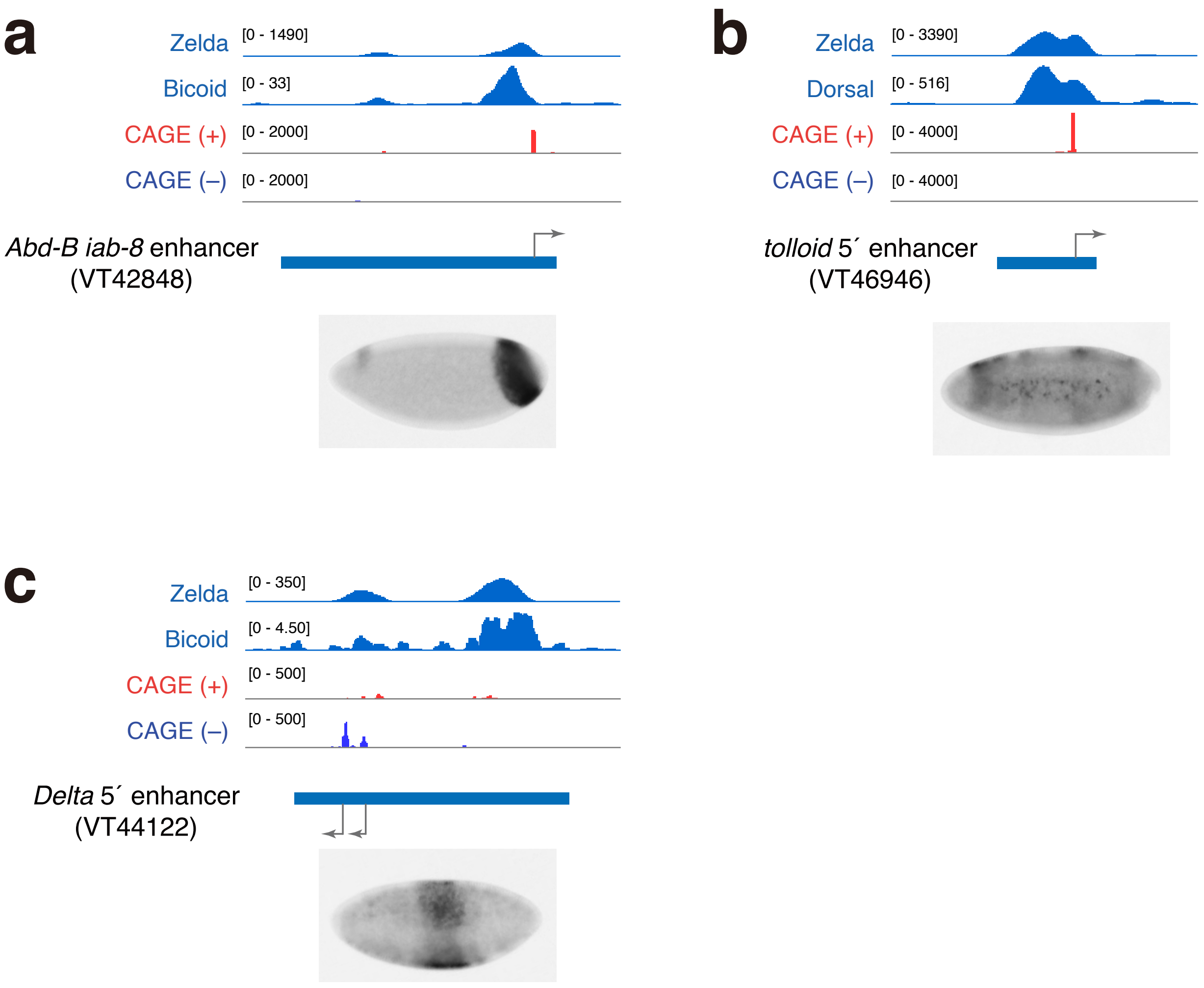
a
Zelda
[0 - 1490]
Bicoid
[0 - 33]
CAGE (+)
[0 - 2000]
CAGE (–)
[0 - 2000]
Abd-B iab-8 enhancer
(VT42848)
b
Zelda
[0 - 3390]
Dorsal
[0 - 516]
CAGE (+)
[0 - 4000]
CAGE (–)
[0 - 4000]
tolloid 5´ enhancer
(VT46946)
c
Zelda
[0 - 350]
Bicoid
[0 - 4.50]
CAGE (+)
[0 - 500]
CAGE (–)
[0 - 500]
Delta 5´ enhancer
(VT44122)

Supplementary Fig. 15. Organization of transcribing enhancers identified in this study.

(a) Organization of the endogenous *Abd-B iab-8* enhancer. Zelda ChIP-seq data from nc13 WT embryos (GSM763061) [1], Bicoid-GFP ChIP-seq from nc14 WT embryos (GSE86966) [4], processed 2- to 4-h WT CAGE-seq data (E-MTAB-4787) [3] after excluding reads mapped to the coding regions were visualized with Integrative Genomics Viewer. A picture showing the activity of *Abd-B iab-8* enhancer (VT42848) was taken from Fly Enhancers [5].

(b) Organization of the endogenous *tolloid* 5′ enhancer. Zelda ChIP-seq data from nc13 WT embryos (GSM763061) [1], Dorsal ChIP-seq from 2- to 4-h WT embryos (GSM1341814) [2], processed 2- to 4-h WT CAGE-seq data (E-MTAB-4787) [3] after excluding reads mapped to the coding regions were visualized with Integrative Genomics Viewer. A picture showing the activity of *tolloid* 5′ enhancer (VT46946) was taken from Fly Enhancers [5].

(c) Organization of the endogenous *Delta* 5′ enhancer. Zelda ChIP-seq data from nc13 WT embryos (GSM763061) [1], Bicoid-GFP ChIP-seq from nc14 WT embryos (GSE86966) [4], processed 2- to 4-h WT CAGE-seq data (E-MTAB-4787) [3] after excluding reads mapped to the coding regions were visualized with Integrative Genomics Viewer. A picture showing the activity of *Delta* 5′ enhancer (VT44122) was taken from Fly Enhancers [5].

Supplementary Fig. 16

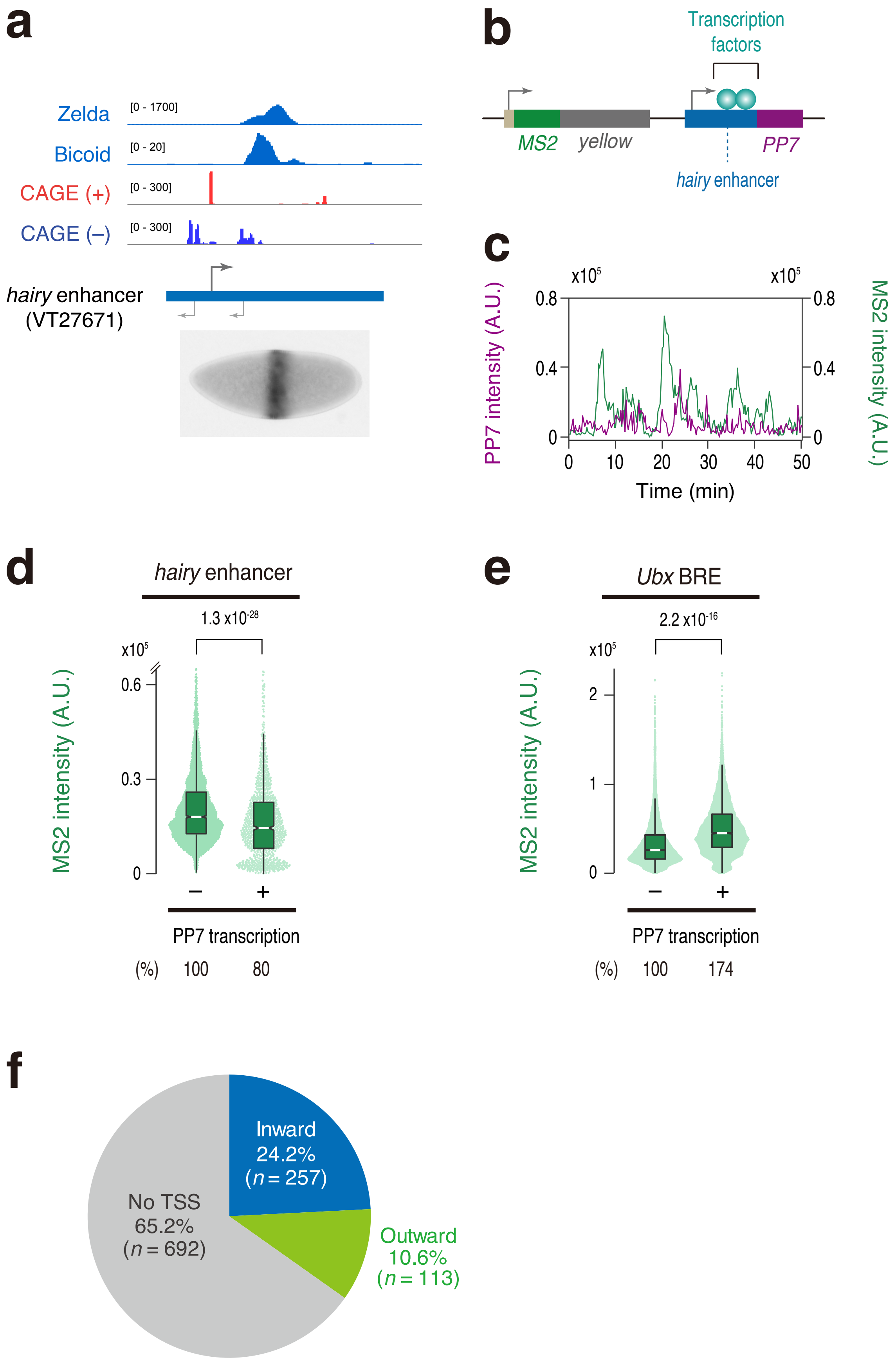

Supplementary Fig. 16. Live-imaging analysis of endogenously transcribing *hairy* enhancer.

(a) Organization of the endogenous *hairy* enhancer. Zelda ChIP-seq data from nc13 WT embryos (GSM763061) [1], Bicoid-GFP ChIP-seq from nc14 WT embryos (GSE86966) [4], processed 2- to 4-h WT CAGE-seq data (E-MTAB-4787) [3] after excluding reads mapped to the coding regions were visualized with Integrative Genomics Viewer. A picture of an early embryo showing the activity of *hairy* enhancer (VT27671; ~2.1 kb in length) was taken from Fly Enhancers [5].

(b) A ~1.3-kb DNA fragment containing *hairy* enhancer was fused with 24x PP7 repeats and placed downstream of the MS2 reporter gene.

(c) Representative trajectories of transcription activities of the reporter locus containing *hairy* enhancer.

(d) Boxplot showing the distribution of instantaneous MS2 activity in the presence or absence of PP7 transcription at the same timeframe. A total of 78 PP7-transcribing nuclei from three independent embryos were used for the analysis. The box indicates the lower (25%) and upper (75%) quantile and the white line indicates the median. Whiskers extend to the most extreme, non-outlier data points.

(e) Boxplot showing the distribution of instantaneous of MS2 activity in the presence or absence of PP7 transcription at the same timeframe. A total of 224 PP7-transcribing nuclei from three independent embryos were used for the analysis The box indicates the lower (25%) and upper (75%) quantile and the white line indicates the median. Whiskers extend to the most extreme, non-outlier data points.

(f) Pie chart showing the distribution of developmental enhancers containing inward TSS, outward TSS, or no TSS facing toward the transcription factor binding sites. A total of 1062 developmental enhancers were analyzed.

A.U.; arbitrary unit. Percentages shown at the bottom of boxplots represent the relative

values of median. P-values were calculated by two-sided Wilcoxon rank sum test. Source data are provided as a Source Data file.

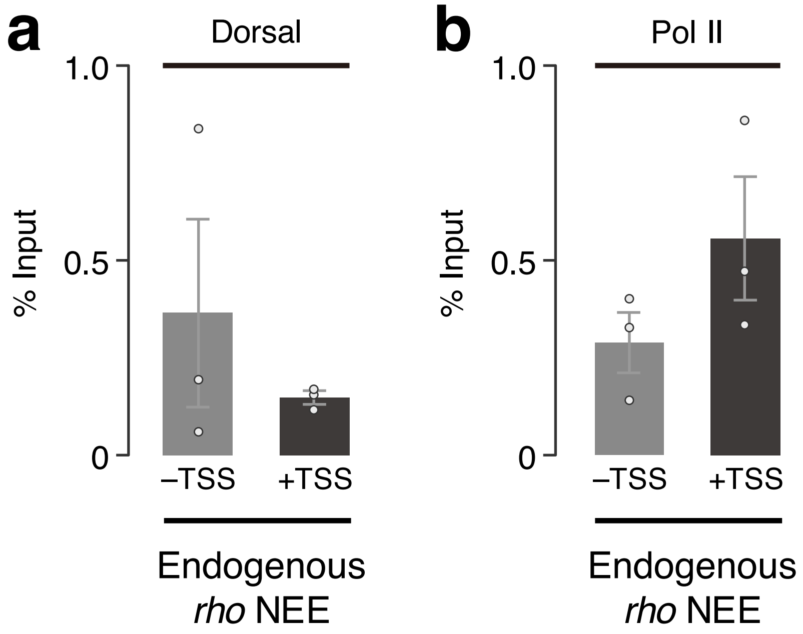
a
Dorsal
1.0
0.5
0
% Input
−TSS
+TSS
Endogenous
rho NEE
b
Pol II
1.0
0.5
0
% Input
−TSS
+TSS
Endogenous
rho NEE

Supplementary Fig. 17. ChIP profiles of Dl and Pol II at the endogenous *rho* NEE.

(a) ChIP-qPCR analysis of Dorsal at the endogenous *rho* NEE. 2-3 h embryos containing (+TSS) or lacking intergenic TSS (–TSS) were used for the analysis. The level of Dorsal binding was expressed as the percentage of input. Three biological replicates were taken for each genome-edited strain.

(b) ChIP-qPCR analysis of Pol II at the endogenous *rho* NEE. 2-3 h embryos containing (+TSS) or lacking intergenic TSS (–TSS) were used for the analysis. The level of Pol II binding was binding was expressed as the percentage of input. Three biological replicates were taken for each fly genome-edited strain.

Error bars represent the mean ± standard error of the mean. Source data are provided as a Source Data file.

Supplementary Table 1: The list of transcribing enhancers identified in this study.

Location of enhancer	FlyEnhancer ID	Target gene	TSS score (reads/kb)	Enhancer size (kb)	Cumulative fraction of TSS score
chr3L:9,797,330-9,797,598	VT28267	*Ilp4*	6007.5	0.268	100
chr3R:20,574,703-20,575,504	VT46946	*tld*	5048.7	0.801	91.55642245
chr3R:20,997,630-20,999,927	VT47167	*danr*	3410.5	2.297	84.46041528
chr3R:12,524,782-12,526,937	VT42746	*Ubx*	3116.0	2.155	79.66685728
chr3R:12,747,382-12,749,489	VT42849	*Abd-B*	2499.3	2.107	75.28726021
chr3R:17,201,451-17,203,140	VT45189	*mod(mdg4)*	2016.0	1.689	71.77447388
chr2L:10,388,040-10,390,080	VT5287	*da*	1903.4	2.04	68.94097607
chr3L:14,749,272-14,751,412	VT30868	*Trl*	1390.2	2.14	66.26567516
chr3R:21,008,302-21,010,513	VT47173	*danr*	1165.1	2.211	64.31174691
chr3R:12,526,519-12,528,610	VT42747	*Ubx*	1122.0	2.091	62.6742046
chr3R:12,075,311-12,077,425	VT42491	*tara*	1034.5	2.114	61.09728558
chr3L:20,396,551-20,398,733	VT33785	*trbl*	999.1	2.182	59.64323601
chr2R:11,016,930-11,019,079	VT17013	*chn*	910.7	2.149	58.23900951
chr2L:2,752,765-2,754,884	VT1404	*Bacc*	870.7	2.119	56.95906889
chr2L:11,794,041-11,794,478	VT6030	*crol*	691.1	0.437	55.735296
chr2R:19,467,816-19,469,873	VT21426	*apt*	664.1	2.057	54.76397915
chr2R:2,583,575-2,585,628	VT12642	*Rab2*	638.1	2.053	53.83061348
chr3L:20,398,155-20,400,412	VT33786	*trbl*	637.1	2.257	52.93376772
chr3R:12,073,519-12,075,931	VT42490	*tara*	610.3	2.412	52.03827359
chr3R:12,745,522-12,747,731	VT42848	*Abd-B*	608.4	2.209	51.18051333
chr3R:18,352,267-18,354,359	VT45801	*CG5346*	600.4	2.092	50.3253699
chr3L:8,645,778-8,647,917	VT27671	*h*	596.5	2.139	49.48152355
chr3L:20,688,420-20,690,975	VT33934	*kni*	576.1	2.555	48.64307715
chr2L:2,162,032-2,164,151	VT1082	*aop*	554.0	2.119	47.83332461
chr2R:20,400,628-20,402,887	VT21906	*Letm1*	504.6	2.259	47.05462034
chr2L:2,163,674-2,165,773	VT1083	*aop*	455.5	2.099	46.34533002
chr3R:7,604,792-7,606,922	VT40165	*Lk6*	452.1	2.13	45.70518132
chr3R:2,684,413-2,686,617	VT37567	*ftz*	444.6	2.204	45.06973027
chr2R:10,060,721-10,062,851	VT16530	*cg*	416.9	2.13	44.44477361
chr2L:8,844,948-8,847,235	VT4450	*SoxN*	405.3	2.287	43.85881252
chr3R:15,166,068-15,168,266	VT44122	*Dl*	397.2	2.198	43.2891089
chr2R:1,597,188-1,599,358	VT12230	*ap*	392.6	2.17	42.73086759
chr2L:21,843,469-21,845,803	VT11145	*tsh*	384.7	2.334	42.17902492
chr3R:15,140,147-15,142,388	VT44107	*Dl*	384.2	2.241	41.63825703
chr2L:21,841,894-21,844,018	VT11144	*tsh*	374.8	2.124	41.09825336
chr3L:9,013,475-9,016,012	VT27864	*Doc1*	368.9	2.537	40.57151619
chrX:5,479,796-5,481,897	VT57351	*Vsx1*	368.4	2.101	40.052966

Supplementary Table 2: The sequences of smiFISH probes used in this study.

	smiFISH probe for *yellow*
yellow_1	CCTCCTAAGTTTCGAGCTGGACTCAGTGAAACTGCGGTCCATGTTTAT
yellow_2	CCTCCTAAGTTTCGAGCTGGACTCAGTGGCCAATCTGGATACGGAATT
yellow_3	CCTCCTAAGTTTCGAGCTGGACTCAGTGCAATCTCCAGCTGTATTTGA
yellow_4	CCTCCTAAGTTTCGAGCTGGACTCAGTGGTAGGCAGTGGTAATACTGT
yellow_5	CCTCCTAAGTTTCGAGCTGGACTCAGTGCCACACTCATCCACTTTAAT
yellow_6	CCTCCTAAGTTTCGAGCTGGACTCAGTGACGGTTCCAGTGTCCAAAAC
yellow_7	CCTCCTAAGTTTCGAGCTGGACTCAGTGCACGGATTAGTGGTGGTATT
yellow_8	CCTCCTAAGTTTCGAGCTGGACTCAGTGGTATCCGTGGTCAAGTCAAA
yellow_9	CCTCCTAAGTTTCGAGCTGGACTCAGTGTAGCTCGTATCTCCGAATTC
yellow_10	CCTCCTAAGTTTCGAGCTGGACTCAGTGGTATTTGGATTTGTGTCCAC
yellow_11	CCTCCTAAGTTTCGAGCTGGACTCAGTGCACGGCAATGTTAGCTATGA
yellow_12	CCTCCTAAGTTTCGAGCTGGACTCAGTGATCATCGCAATTTTTGCCTA
yellow_13	CCTCCTAAGTTTCGAGCTGGACTCAGTGTATCCCAATTCATCGGCAAA
yellow_14	CCTCCTAAGTTTCGAGCTGGACTCAGTGCCCAGGAGTAAGCAATCAAG
yellow_15	CCTCCTAAGTTTCGAGCTGGACTCAGTGAGAATCTCCAGGACTTGTTC
yellow_16	CCTCCTAAGTTTCGAGCTGGACTCAGTGCCTCAATGGATCGGGGAAAA
yellow_17	CCTCCTAAGTTTCGAGCTGGACTCAGTGCCCATTGGAAGTTAATACCA
yellow_18	CCTCCTAAGTTTCGAGCTGGACTCAGTGATACCAAATATACCCTCCTC
yellow_19	CCTCCTAAGTTTCGAGCTGGACTCAGTGCGATCGAATGGGCGAAAGGG
yellow_20	CCTCCTAAGTTTCGAGCTGGACTCAGTGAGTACAGGGTACGATAACCA
yellow_21	CCTCCTAAGTTTCGAGCTGGACTCAGTGCGATGACTTGCTAACGGACT
yellow_22	CCTCCTAAGTTTCGAGCTGGACTCAGTGAAAATCCTCGTGGATACGGC
yellow_23	CCTCCTAAGTTTCGAGCTGGACTCAGTGCATGATAGCTATCTTCCGTC
yellow_24	CCTCCTAAGTTTCGAGCTGGACTCAGTGCCGTTCATCTAAGGCAACAA
yellow_25	CCTCCTAAGTTTCGAGCTGGACTCAGTGCACGTGAAGTGGTATGGGAG
yellow_26	CCTCCTAAGTTTCGAGCTGGACTCAGTGACAGCTCAATTCCATCATCG
yellow_27	CCTCCTAAGTTTCGAGCTGGACTCAGTGCCACTGCATTTTGATCTATT
yellow_28	CCTCCTAAGTTTCGAGCTGGACTCAGTGGAGTACGGCATTGATGAGTG
yellow_29	CCTCCTAAGTTTCGAGCTGGACTCAGTGCCACAATGCCATGAAATTGC
yellow_30	CCTCCTAAGTTTCGAGCTGGACTCAGTGAACTAAGCCAACGTCATCGC
yellow_31	CCTCCTAAGTTTCGAGCTGGACTCAGTGCATCAATTTTCACATCGGCC
yellow_32	CCTCCTAAGTTTCGAGCTGGACTCAGTGCCTATCGGATAGAACCCAAA
yellow_33	CCTCCTAAGTTTCGAGCTGGACTCAGTGATCCAAGTCAGACAGCAAGA
yellow_34	CCTCCTAAGTTTCGAGCTGGACTCAGTGGGAGCCGTGTAAATTCGGAA
yellow_35	CCTCCTAAGTTTCGAGCTGGACTCAGTGATTCTCAATTAAAGTGGCCA
yellow_36	CCTCCTAAGTTTCGAGCTGGACTCAGTGAGGCGTTATTCCTCAAATCA
yellow_37	CCTCCTAAGTTTCGAGCTGGACTCAGTGTTGAAACGGTATTTGGCGGC
yellow_38	CCTCCTAAGTTTCGAGCTGGACTCAGTGGGCAAAACGGCTTGTTTTGG
yellow_39	CCTCCTAAGTTTCGAGCTGGACTCAGTGTTCGTATATAACGGTGGACC
yellow_40	CCTCCTAAGTTTCGAGCTGGACTCAGTGTTTCTGTGGCAAGACAGGAC
yellow_41	CCTCCTAAGTTTCGAGCTGGACTCAGTGCGGGCAAATAAGTGCGACTT
yellow_42	CCTCCTAAGTTTCGAGCTGGACTCAGTGTGGAGACTACATTGCCTGAA
yellow_43	CCTCCTAAGTTTCGAGCTGGACTCAGTGGGACCCACAGAATTTGTAGA
yellow_44	CCTCCTAAGTTTCGAGCTGGACTCAGTGCCGTTGTGCTGGTTGAAAAT
yellow_45	CCTCCTAAGTTTCGAGCTGGACTCAGTGGACCACTTGTCTCGTAATTT
yellow_46	CCTCCTAAGTTTCGAGCTGGACTCAGTGGGGTTGATGGGTGGGAAATA
yellow_47	CCTCCTAAGTTTCGAGCTGGACTCAGTGCGGGCATTCACATAAGTTTT
yellow_48	CCTCCTAAGTTTCGAGCTGGACTCAGTGAACCTTGATGCTGATGATGC

Plasmid construction

pbphi-nanos>NLS-mCherry-MCP

A DNA fragment containing MCP was amplified from pbphi-nanos>MCP-GFP [6] using primers (5′-AAA AGC TAG CAT GGC TTC TAA CTT TAC TCA-3′) and (5′-TTT TGG ATC CTT AGT AGA TGC CGG AGT TTG-3′), and digested with NheI and BamHI. The resulting fragment was inserted between the NheI and BamHI sites of pbphi-nanos>NLS-mCherry-PCP-NES [7] to replace PCP-NES cassette to MCP.

pbphi-nanos>NLS-mCherry-MCP, nanos>NLS-mTagBFP2-PCP

A DNA fragment containing SV40NLS and mTagBFP2 was inserted between the XhoI and HindIII sites of pbphi-nanos>SV40NLS-mCherry-PCP [8]. Then, a DNA fragment containing *nanos* promoter region with XbaI site was inserted between NotI and XhoI sites of nanos>NLS-mTagBFP2-PCP to add extra XbaI site. Subsequently, the plasmid was digested with XbaI, and the resulting fragment containing nanos>NLS-mTagBFP2-PCP was inserted into the unique XbaI site in the pbphi-nanos>NLS-mCherry-MCP.

pbphi-PP7-sna shadow enhancer

A DNA fragment containing *sna* shadow enhancer was amplified using primers (5′-TTT AAG GAT CCA AGC TTG CAT TGA GGT GTT TTG-3′) and (5′-TTA AAA GAT CTG CTA GCT AAA TTC CGA TTT TTC-3′), and digested with BamHI and BglII. The resulting fragment was inserted into the unique BamHI site in pbphi-αTubulin 3′UTR [8]. Subsequently, pBlueScript-24xPP7 [9] was digested with BamHI and BglII. The resulting fragment containing 24xPP7 was inserted into the unique BamHI site in the plasmid.

pbphi-No TSS-PP7-sna shadow enhancer

A DNA fragment containing a partial sequence from *lacZ* gene was amplified using primers (5′-TTT AAG CGG CCG CTC TAG AGA CGG CAG TTA TCT GGA AGA-3′) and (5′-TTA AAG GAT CCA CTT CAG CCT CCA GTA CAG C-3′), and digested with NotI and BamHI. The resulting fragment was inserted between the NotI and BamHI sites in pbphi-PP7-sna shadow enhancer.

pbphi-TSS-PP7-sna shadow enhancer

A DNA fragment containing DSCP was amplified from pbphi-DSCP-MS2-yellow [10] using primers (5′-TTT AAG CGG CCG CTC TAG AGA GCT CGC CCG GGG ATC GAG CGC-3′) and (5′-TTA AAG GAT CCG TTT GGT ATG CGT CTT GTG A-3′), and digested with NotI and BamHI. The resulting fragment was inserted between the NotI and BamHI sites in pbphi-PP7-sna shadow enhancer.

pbphi-TSS^{weak}-PP7-sna shadow enhancer

A DNA fragment containing DSCP with Inr mutation was amplified from pbphi-DSCP$_{mInr}$-MS2-yellow-sna shadow enhancer [11] using primers (5′-TTT AAG CGG CCG CTC TAG AGA GCT CGC CCG GGG ATC GAG CGC-3′) and (5′-TTA AAG GAT CCG TTT GGT ATG CGT CTT GTG A-3′), and digested with NotI and BamHI. The resulting fragment was inserted between the NotI and BamHI sites in pbphi-PP7-sna shadow enhancer.

pbphi-TSS^{inverted}-PP7-sna shadow enhancer

A DNA fragment containing DSCP was amplified from pbphi-DSCP-MS2-yellow [10] using primers (5′-TTT AAG CGG CCG CTC TAG AGT TTG GTA TGC GTC TTG TGA-3′) and (5′-TTA AAG GAT CCG AGC TCG CCC GGG GAT CGA G-3′), and digested with NotI and BamHI. The resulting fragment was inserted between the NotI and

BamHI sites in pbphi-PP7-sna shadow enhancer.

pbphi-DSCP-MS2-yellow-sna shadow enhancer-PP7-No TSS

pbphi-No TSS-PP7-sna shadow enhancer was digested with XbaI. The resulting fragment containing No TSS-PP7-*sna* shadow enhancer was inserted into the unique XbaI site in pbphi-DSCP-MS2-yellow [10]. Orientation of the inserted DNA fragment was confirmed by sequencing.

pbphi-DSCP-MS2-yellow-sna shadow enhancer-PP7-TSS

pbphi-TSS-PP7-sna shadow enhancer was digested with XbaI. The resulting fragment containing TSS-PP7-*sna* shadow enhancer was inserted into the unique XbaI site in pbphi-DSCP-MS2-yellow [10] . Orientation of the inserted DNA fragment was confirmed by sequencing.

pbphi-DSCP-MS2-yellow-sna shadow enhancer-PP7-TSS^{weak}

pbphi-TSS^{weak}-PP7-sna shadow enhancer was digested with XbaI. The resulting fragment containing TSS^{weak}-PP7-*sna* shadow enhancer was inserted into the unique XbaI site in pbphi-DSCP-MS2-yellow [10]. Orientation of the inserted DNA fragment was confirmed by sequencing.

pbphi-DSCP-MS2-yellow-sna shadow enhancer-PP7-TSS^{inverted}

pbphi-TSS^{inverted}-PP7-sna shadow enhancer was digested with XbaI. The resulting fragment containing TSS^{inverted}-PP7-*sna* shadow enhancer was inserted into the unique XbaI site in pbphi-DSCP-MS2-yellow [10]. Orientation of the inserted DNA fragment was confirmed by sequencing.

pbphi-DSCP-MS2-yellow-TSS-PP7-sna shadow enhancer

pbphi-TSS-PP7-sna shadow enhancer was digested with XbaI. The resulting fragment containing TSS-PP7-*sna* shadow enhancer was inserted into the unique XbaI site in pbphi-DSCP-MS2-yellow [10]. Orientation of the inserted DNA fragment was confirmed by sequencing.

pbphi-TSS-PP7-spacer

A DNA fragment containing a partial sequence from *lacZ* gene was amplified using primers (5′-GGG TTA AGC TTC TGC AGG AAT CCG ACG GGT TGT TAC T-3′) and (5′-GGG AAG CTA GCC GGA TAA ACG GAA CTG GAA A-3′), and digested with HindIII and NheI. The resulting fragment was inserted between the HindIII and NheI sites in pbphi-TSS-PP7-sna shadow enhancer.

pbphi-DSCP-MS2-yellow-sna shadow enhancer-TSS-PP7

A DNA fragment containing *sna* shadow enhancer was amplified using primers (5′-AAG GAG CTA GCG CAT TGA GGT GTT TTG TTG G-3′) and (5′-GGG GGT CTA GAT AAA TTC CGA TTT TTC TTG TCT GGG-3′), and digested with NheI and XbaI. The resulting fragment was inserted into the unique XbaI site in pbphi-DSCP-MS2-yellow [10]. Subsequently, pbphi-TSS-PP7-spacer was digested with XbaI. The resulting fragment containing TSS-PP7-spacer was inserted into the unique XbaI site in the plasmid. Orientation of the inserted DNA fragment was confirmed by sequencing.

pbphi-No TSS-PP7-rho NEE

A DNA fragment containing *rho* NEE was inserted between the HindIII and NheI sites in pbphi-No TSS-PP7-sna shadow enhancer. Sequence of *rho* NEE is the same as one used in the previous study [9].

pbphi-TSS-PP7-rho NEE

A DNA fragment containing *rho* NEE was inserted between the HindIII and NheI sites in pbphi-TSS-PP7-sna shadow enhancer. Sequence of *rho* NEE is the same as one used in the previous study [9].

pbphi-DSCP-MS2-yellow-rho NEE-PP7-No TSS

pbphi-No TSS-PP7-rho NEE was digested with XbaI. The resulting fragment containing No TSS-PP7-*rho* NEE was inserted into the unique XbaI site in pbphi-DSCP-MS2-yellow [10]. Orientation of the inserted DNA fragment was confirmed by sequencing.

pbphi-DSCP-MS2-yellow-rho NEE-PP7-TSS

pbphi-TSS-PP7-rho NEE was digested with XbaI. The resulting fragment containing TSS-PP7-*rho* NEE was inserted into the unique XbaI site in pbphi-DSCP-MS2-yellow [10]. Orientation of the inserted DNA fragment was confirmed by sequencing.

pbphi-DSCP-MS2-yellow-rhoNEE-TSS-PP7

A DNA fragment containing *rho* NEE was amplified using primers (5′-GGG GAG CTA GCT TCC TCT GCT CAA AAT CAA A-3′) and (5′-GGA AAT CTA GAC CTC AGG TCG AGT TCC TCC A-3′), and digested with NheI and XbaI. The resulting fragment was inserted into the unique XbaI site in pbphi-DSCP-MS2-yellow [10]. Subsequently, pbphi-TSS-PP7-spacer was digested with XbaI. The resulting fragment containing TSS-PP7-spacer was inserted into the unique XbaI site in the plasmid. Orientation of the inserted DNA fragment was confirmed by sequencing.

pbphi-sna shadow enhancer-PP7

A DNA fragment containing *sna* shadow enhancer was purified from pbphi-sna shadow enhancer [6] by digesting with NotI and BamHI, and inserted between the NotI and BamHI sites of pbphi-lacZ-PP7-αTubulin 3′UTR [8]. Subsequently, primers (5′-GGC CGC TCT AGA CTC GAG AGT TTA-3′) and (5′-AGC TTA AAC TCT CGA GTC TAG AGC-3′) were annealed and inserted between the NotI and HindIII sites in the plasmid.

pbphi-DSCP-MS2-yellow-Ubx BRE-PP7

A DNA fragment containing *Ubx* BRE was amplified from genomic DNA using primers (5′-TTT TTG CTA GCA CTT CCA CTC GAA TTG CGC C-3′) and (5′-TTG GGA AGC TTT AAA TTC TCA GGC GGC ACG A-3′), and digested with NheI and HindIII. The resulting fragment was inserted between the NheI and HindIII sites in pBlueScript. Subsequently, pBlueScript-Ubx BRE was digested with NheI and HindIII, and the resulting DNA fragment was inserted between the NheI and HindIII site in pbphi-sna shadow enhancer-PP7 plasmid. Then, pbphi-Ubx BRE-PP7 was digested with XbaI, and the resulting DNA fragment was inserted into the unique XbaI site in pbphi-DSCP-MS2-yellow [10]. Orientation of the inserted DNA fragment was confirmed by sequencing.

pbphi-DSCP-MS2-yellow-inverted Ubx BRE-PP7

A DNA fragment containing *Ubx* BRE was inserted between the NheI and HindIII sites in pbphi-No TSS-PP7-sna shadow enhancer. Resulting pbphi-inverted Ubx BRE-PP7 was digested with XbaI, and the DNA fragment containing inverted *Ubx* BRE-PP7 was inserted into the unique XbaI site in pbphi-DSCP-MS2-yellow [10]. Orientation of the inserted DNA fragment was confirmed by sequencing.

pbphi-DSCP-MS2-yellow-inverted Ubx BRE-PP7-TSS

A DNA fragment containing *Ubx* BRE was inserted between the NheI and HindIII sites

in pbphi-TSS-PP7-sna shadow enhancer. Resulting pbphi-inverted Ubx BRE-PP7-TSS was digested with XbaI, and the DNA fragment containing inverted *Ubx* BRE-PP7-TSS was inserted into the unique XbaI site in pbphi-DSCP-MS2-yellow [10]. Orientation of the inserted DNA fragment was confirmed by sequencing.

pbphi-DSCP-MS2-yellow-2.5-kb spacer

A DNA fragment containing a partial sequence from *lacZ* gene was amplified using primers (5′-TTT TTG CTA GCC GGT TAC GAT GCG CCC ATC T-3′) and (5′-TTT GGT CTA GAC AAT GGC AGA TCC CAG CGG T-3′), and digested with NheI and XbaI. The resulting fragment was inserted into the unique XbaI site in pbphi-DSCP-MS2-yellow [10]. Orientation of the inserted DNA fragment was confirmed by sequencing.

pbphi-DSCP-MS2-yellow-2.5-kb spacer-sna shadow enhancer-PP7-No TSS

pbphi-No TSS-PP7-sna shadow enhancer was digested with XbaI, and the resulting DNA fragment containing No TSS-PP7-*sna* shadow enhancer was inserted into the unique XbaI site in pbphi-DSCP-MS2-yellow-2.5-kb spacer. Orientation of the inserted DNA fragment was confirmed by sequencing.

pbphi-DSCP-MS2-yellow-2.5-kb spacer-sna shadow enhancer-PP7-TSS

pbphi-TSS-PP7-sna shadow enhancer was digested with XbaI, and the resulting DNA fragment containing TSS-PP7-*sna* shadow enhancer was inserted into the unique XbaI site in pbphi-DSCP-MS2-yellow-2.5-kb spacer. Orientation of the inserted DNA fragment was confirmed by sequencing.

pbphi-DSCP-MS2-yellow-sna shadow enhancer-$TSS^{inverted}$-TSS-PP7

A DNA fragment containing unrelated 100-bp spacer sequence was amplified using

primers (5′-GGG GGT CTA GAA TAA AAA TAG GCG TAT CAC G-3′) and (5′-GGT TTG CTA GCG TTT GGT ATG CGT CTT GTG A-3′), and digested with NheI and XbaI. The resulting fragment was inserted into the unique XbaI site in pbphi-DSCP-MS2-yellow-sna shadow enhancer. Subsequently, a DNA fragment containing $TSS^{inverted}$ was amplified using primers (5′-GGG GGT CTA GAA TAA AAA TAG GCG TAT CAC G-3′) and (5′-GGT TTG CTA GCG TTT GGT ATG CGT CTT GTG A-3′), and digested with NheI and XbaI. The resulting fragment was inserted into the unique XbaI site in pbphi-DSCP-MS2-yellow-sna shadow enhancer-100-bp spacer. Finally, pbphi-TSS-PP7-spacer was digested with XbaI, and the resulting fragment was inserted into the unique XbaI site in the plasmid.

pbphi-DSCP-MS2-yellow-hairy enhancer-PP7

A DNA fragment containing *hairy* enhancer was amplified from genomic DNA using primers (5′-GGT TTA AGC TTC TAC CTG GGT CTC CAC AGA ACT CC-3′) and (5′-GGG TTG CTA GCG ATG ATG TGA TCC TAT TTT CCT CG-3′), and digested with NheI and HindIII. The resulting fragment was inserted between the NheI and HindIII sites in pbphi-Ubx BRE-PP7 plasmid. Then, pbphi-hairy enhancer-PP7 was digested with XbaI, and the resulting DNA fragment containing *hairy* enhancer-PP7 was inserted into the unique XbaI site in pbphi-DSCP-MS2-yellow [10]. Orientation of the inserted DNA fragment was confirmed by sequencing.

pCFD3-dU6-dl gRNA

Two DNA oligos (5′-GTC GGC AAT CAA GCG GAT AAT AA-3′) and (5′-AAA CTT ATT ATC CGC TTG ATT GC-3′) were annealed and inserted into the pCFD3-dU6:3gRNA vector (addgene# 49410) using BbsI sites.

pBS-dl 5′arm-GFP-3xFLAG-loxP-3xP3-dsRed-loxP-dl 3′arm

A DNA fragment containing 5′ homology arm of *dl* was amplified from genomic DNA using primers (5′-GGG GAG GTA CCG AAC CAA GAG GTG AGT TTT ATA CAC-3′) and (5′-AAA AGT CGA CCG TGG ATA TGG ACA GGT TCG ATA TCT GCA GAT CTT CCG AAT TGA GGC GCA GTA TCT GCT GAT CCT CTG AGT TTA TGT GCA CCA ACT GCC CGC TAT CGA AGC TAA GCA GAT TGC TGA GCG TTG GCG CAT TAT TAT CCG CTT GAT TGC CAG C-3′), and digested with KpnI and SalI. The resulting fragment was inserted between the KpnI and SalI sites in pBS-GFP-3xFLAG-loxP-3xP3-dsRed-loxP. Subsequently, a DNA fragment containing 3′ homology arm of *dl* was amplified from genomic DNA using primers (5′-GGG GGA CTA GTT AAT GGG CCA ACG CTC AGC AAT CTG-3′) and (5′-AAA AAG CGG CCG CGG GTG GGC AGC TTA TCC ACA-3′), and digested with SpeI and NotI. The resulting fragment was inserted between the SpeI and NotI sites in the plasmid.

pCFD3-dU6-zld gRNA

Two DNA oligos (5′-GTC GCA AGA GCG AGT ACG TGC AGG-3′) and (5′-AAA CCC TGC ACG TAC TCG CTC TTG-3′) were annealed and inserted into the pCFD3-dU6:3gRNA vector (addgene# 49410) using BbsI sites.

pBS-zld 5′arm-GFP-3xFLAG-loxP-3xP3-dsRed-loxP-zld 3′arm

A DNA fragment containing 5′ homology arm of *zld* was amplified from genomic DNA using primers (5′-TTA AAG GTA CCC GCC CTA CTC GCC CAC AGT GAG C-3′) and (5′-GGG AAG TCG ACG TAG AGC TCT ATG CTC TTC TCG ATC ATC TGA AAC TGC TCC TGC ACG TAC-3′), and digested with KpnI and SalI. The resulting fragment was inserted between the KpnI and SalI sites in pBS-GFP-3xFLAG-loxP-3xP3-dsRed-loxP. Subsequently, a DNA fragment containing 3′ homology arm of *zld* was amplified

from genomic DNA using primers (5′-TTA AAT CTA GAA GGA GGA GTT TCA GAT GAT CGA G-3′) and (5′-GGG AAG CGG CCG CGC AAT GCG TTG GTC TAG TAA C-3′), and digested with XbaI and NotI. The resulting fragment was inserted between the XbaI and NotI sites in the plasmid.

pCFD3-dU6-rho 3′UTR

Two DNA oligos (5′-GTC GGA GTC GTC AGC ATG CTC GC-3′) and (5′-AAA CGC GAG CAT GCT GAC GAC TC-3′) were annealed and inserted into the pCFD3-dU6:3gRNA vector (addgene # 49410) using BbsI sites.

pBS-rho 5′Arm-24xMS2-dsRed-SV40-rho 3′Arm

A DNA fragment containing 5′ homology arm of *rho* was amplified from genomic DNA using primers (5′-TTT AAG GTA CCG GCA GGA CTC GCC CCT AAG CA-3′) and (5′-TTA AAC TCG AGA GCA TGC TGA CGA CTC CGA ACC TC-3′) and digested with KpnI and XhoI. The resulting fragment was inserted between the KpnI and XhoI sites of pBS-loxP-MS2-dsRed-loxP [6]. Subsequently, a DNA fragment containing 3′ homology arm of *rho* was amplified from genomic DNA using primers (5′-TTT AAT CTA GAC GCA GGG ATT CGG AAT CTG CTT GAG-3′) and (5′-TTA AAG CGG CCG CCC AAG CGA GCC GAC AGC CAA AAA C-3′) and digested with XbaI and NotI. The resulting fragment was inserted between the XbaI and NotI sites of the plasmid.

pCFD3-dU6-sna 3′UTR

Two DNA oligos (5′-GTC GCG ACA TAT GAA TCC CTT AGC-3′) and (5′-AAA CGC TAA GGG ATT CAT ATG TCG-3′) were annealed and inserted into the pCFD3-dU6:3gRNA vector (addgene # 49410) using BbsI sites.

pBS-sna 5′Arm-24xMS2-dsRed-SV40-sna 3′Arm

A DNA fragment containing 5′ homology arm of *sna* was amplified from genomic DNA using primers (5′-TTT AAC TCG AGC AGC AGC CGG AAC CGA AAC GTG AC-3′) and (5′-TTA AAG AAT TCA AGG GAT TCA TAT GTC GAG AAT CC-3′) and digested with XhoI and EcoRI. The resulting fragment was inserted between the XhoI and EcoRI sites of pBS-loxP-MS2-dsRed-loxP [6]. Subsequently, a DNA fragment containing 3′ homology arm of *sna* was amplified from genomic DNA using primers (5′-TTT AAA CTA GTA GCA GGA CAC AAT TAC CTA AGC-3′) and (5′-TTA AAG CGG CCG CCC GTC TGC CAG TCA ATC AGG AAG G-3′) and digested with SpeI and NotI. The resulting fragment was inserted between the SpeI and NotI sites of the plasmid.

Supplementary References

1. Harrison, M.M., Li, X.Y., Kaplan, T., Botchan, M.R. & Eisen, M.B. Zelda binding in the early *Drosophila melanogaster* embryo marks regions subsequently activated at the maternal-to-zygotic transition. *PLoS Genet* **7**, e1002266 (2011).
2. He, Q., Johnston, J. & Zeitlinger, J. ChIP-nexus enables improved detection of in vivo transcription factor binding footprints. *Nat Biotechnol* **33**, 395-401 (2015).
3. Schor, I.E. *et al*. Promoter shape varies across populations and affects promoter evolution and expression noise. *Nat Genet* **49**, 550-558 (2017).
4. Hannon, C.E., Blythe, S.A. & Wieschaus, E.F. Concentration dependent chromatin states induced by the bicoid morphogen gradient. *Elife* **6**, e28275 (2017).
5. Kvon, E.Z. *et al*. Genome-scale functional characterization of *Drosophila* developmental enhancers *in vivo*. *Nature* **512**, 91-95 (2014).
6. Lim, B., Heist, T., Levine, M. & Fukaya, T. Visualization of transvection in living *Drosophila* embryos. *Mol Cell* **70**, 287-296 e6 (2018).
7. Fukaya, T. Dynamic regulation of anterior-posterior patterning genes in living *Drosophila* embryos. *Curr Biol* **31**, 2227-2236 e6 (2021).
8. Fukaya, T., Lim, B. & Levine, M. Rapid rates of Pol II elongation in the *Drosophila* embryo. *Curr Biol* **27**, 1387-1391 (2017).
9. Fukaya, T., Lim, B. & Levine, M. Enhancer control of transcriptional bursting. *Cell* **166**, 358-368 (2016).
10. Yokoshi, M., Segawa, K. & Fukaya, T. Visualizing the role of boundary elements in enhancer-promoter communication. *Mol Cell* **78**, 224-235 e5 (2020).
11. Yokoshi, M., Kawasaki, K., Cambon, M. & Fukaya, T. Dynamic modulation of enhancer responsiveness by core promoter elements in living *Drosophila* embryos. *Nucleic Acids Res* **50**, 92-107 (2022).
