# Supplementary material for: Dynamic interplay between non-coding enhancer transcription and gene activity in development
Source: Nat Commun. 2023 Feb 20;14:826. doi: 10.1038/s41467-023-36485-1 (PMC9941499; doi:10.1038/s41467-023-36485-1)
Supplement: Supplementary file 2 — Description of Additional Supplementary Files [file 41467_2023_36485_MOESM2_ESM.pdf]

Descriptions of additional supplementary files

Supplementary Movie 1. Live-imaging of embryos driving noncoding enhancer transcription in cis. MS2/PP7 two-color live-imaging of embryos with the reporter locus containing – TSS (top), + TSS (middle), or TSSweak (bottom) at the enhancer region. The maximum projected images of PP7, MS2 and His2Av signal are shown in magenta, green and grey, respectively. The image is oriented with anterior to the left and ventral view facing up. Scale bar indicates 30 µm.

Supplementary Movie 2. Live-imaging of embryos driving noncoding enhancer transcription in trans. MS2/PP7 two-color live-imaging of embryos lacking (top) or containing (bottom) the PP7 allele. The maximum projected images of PP7, MS2 and His2Av signal are shown in magenta, green and grey, respectively. The image is oriented with anterior to the left and ventral view facing up. Scale bar indicates 30 µm.

Supplementary Movie 3. Live-imaging of embryos driving non-coding enhancer transcription in a tandem orientation. MS2/PP7 two-color live-imaging of embryos with the reporter locus driving non-coding enhancer transcription in a convergent (top) or a tandem orientation (bottom). The maximum projected images of PP7, MS2 and His2Av signal are shown in magenta, green and grey, respectively. The image is oriented with anterior to the left and ventral view facing up. Scale bar indicates 30 µm. Movie of convergent is the same as the movie of + TSS shown in Supplementary Movie 1.

Supplementary Movie 4. Live-imaging of embryos driving PP7 transcription in an outward orientation. MS2/PP7 two-color live-imaging of embryos driving PP7 transcription in an inward (top) or an outward orientation (bottom). The maximum projected images of PP7, MS2 and His2Av signal are shown in magenta, green and grey, respectively. The image is oriented with anterior to the left and ventral view facing up. Scale bar indicates 30 µm.

Supplementary Movie 5. Calculation of average Dorsal distribution around non-inhibitory PP7 transcription site. Top panel shows images of the PP7 signal, corresponding Dorsal-GFP signal in the same window, and Dorsal-GFP signal at a random site in the same nucleus. Middle panel shows a running average of the images in the top panel. Bottom panel shows the corresponding running average radial profile. A total of 839 nuclei from 50 independent embryos were analyzed.

Supplementary Movie 6. Calculation of average Dorsal distribution around inhibitory PP7 transcription site. Top panel shows images of the PP7 signal, corresponding Dorsal-GFP signal in the same window, and Dorsal-GFP signal at a random site in the same nucleus. Middle panel shows a running average of the images in the top panel. Bottom panel shows the corresponding running average radial profile. A total of 852 nuclei from 50 independent embryos were analyzed.

Supplementary Movie 7. Live-imaging of embryos driving noncoding enhancer transcription from Ubx BRE. MS2/PP7 two-color live-imaging of embryos with the reporter locus driving PP7 transcription from BRE. The maximum projected images of PP7, MS2 and His2Av signal are shown in magenta, green and grey, respectively. The image is oriented with anterior to the left and lateral view facing up. Scale bar indicates 30 µm.

Supplementary Movie 8. Low magnification live-imaging movie of embryos driving non-coding enhancer transcription from Ubx BRE. MS2/PP7 two-color live-imaging of embryos with the reporter locus driving PP7 transcription from BRE. The maximum projected images of PP7, MS2 and His2Av signal are shown in magenta, green and blue, respectively. The image is oriented with anterior to the left and lateral view facing up. Scale bar indicates 30 µm.
