# Supplementary material for: Dynamic interplay between non-coding enhancer transcription and gene activity in development
Source: Nat Commun. 2023 Feb 20;14:826. doi: 10.1038/s41467-023-36485-1 (PMC9941499; doi:10.1038/s41467-023-36485-1)
Supplement: Supplementary file 11 — Reporting Summary [file 41467_2023_36485_MOESM11_ESM.pdf]

nature portfolio

Corresponding author(s): Takashi Fukaya

Last updated by author(s): Jan 18, 2023

Reporting Summary

Nature Portfolio wishes to improve the reproducibility of the work that we publish. This form provides structure for consistency and transparency in reporting. For further information on Nature Portfolio policies, see our Editorial Policies and the Editorial Policy Checklist.

Statistics

For all statistical analyses, confirm that the following items are present in the figure legend, table legend, main text, or Methods section.

n/a	Confirmed	
☐	☒	The exact sample size (n) for each experimental group/condition, given as a discrete number and unit of measurement
☐	☒	A statement on whether measurements were taken from distinct samples or whether the same sample was measured repeatedly
☐	☒	The statistical test(s) used AND whether they are one- or two-sided<br>*Only common tests should be described solely by name; describe more complex techniques in the Methods section.*
☒	☐	A description of all covariates tested
☒	☐	A description of any assumptions or corrections, such as tests of normality and adjustment for multiple comparisons
☐	☒	A full description of the statistical parameters including central tendency (e.g. means) or other basic estimates (e.g. regression coefficient) AND variation (e.g. standard deviation) or associated estimates of uncertainty (e.g. confidence intervals)
☐	☒	For null hypothesis testing, the test statistic (e.g. F, t, r) with confidence intervals, effect sizes, degrees of freedom and P value noted<br>*Give P values as exact values whenever suitable.*
☒	☐	For Bayesian analysis, information on the choice of priors and Markov chain Monte Carlo settings
☒	☐	For hierarchical and complex designs, identification of the appropriate level for tests and full reporting of outcomes
☒	☐	Estimates of effect sizes (e.g. Cohen's d, Pearson's r), indicating how they were calculated

Our web collection on statistics for biologists contains articles on many of the points above.

Software and code

Policy information about availability of computer code

Data collection	Zeiss ZEN software (version 3.1) for Zeiss LSM900 confocal microscope<br>LightCycler 480 Software (release 1.5.1.62) for RT-qPCR and ChIP-qPCR<br>Stellaris RNA FISH probe designer tool for designing smiFISH probes
Data analysis	Fiji (v1.53q) for image analysis<br>MATLAB (R2021b) for image analysis<br>R (v4.1.2) for statistical analysis<br>IGV (v2.12.3) for visualization of genome profile<br>HISAT2 (version 2.2.0) for mapping of public CAGE-seq data<br>Samtools (version 1.10) for conversion of analyzed data format<br>bedtools (version 2.29.2) for editing and analysis of bed files.

For manuscripts utilizing custom algorithms or software that are central to the research but not yet described in published literature, software must be made available to editors and reviewers. We strongly encourage code deposition in a community repository (e.g. GitHub). See the Nature Portfolio guidelines for submitting code & software for further information.

Data

Policy information about availability of data

All manuscripts must include a data availability statement. This statement should provide the following information, where applicable:

- Accession codes, unique identifiers, or web links for publicly available datasets
- A description of any restrictions on data availability
- For clinical datasets or third party data, please ensure that the statement adheres to our policy

Previously published sequence data are available under accession number GSE30757, GSE86966, GSE55306, GSE36212 and E-MTAB-4787.
The original live-imaging data used for the analysis shown in main and supplementary figures have been deposited to Zenodo database (https://doi.org/10.5281/zenodo.7545929).
The MATLAB codes for nuclei tracking and MS2/PP7 signal recording are available at the Zenodo database (https://doi.org/10.5281/zenodo.7545929).

Human research participants

Policy information about studies involving human research participants and Sex and Gender in Research.

Reporting on sex and gender	Not applicable.
Population characteristics	Not applicable.
Recruitment	Not applicable.
Ethics oversight	Not applicable.

Note that full information on the approval of the study protocol must also be provided in the manuscript.

Field-specific reporting

Please select the one below that is the best fit for your research. If you are not sure, read the appropriate sections before making your selection.

[x] Life sciences [ ] Behavioural & social sciences [ ] Ecological, evolutionary & environmental sciences

For a reference copy of the document with all sections, see nature.com/documents/nr-reporting-summary-flat.pdf

Life sciences study design

All studies must disclose on these points even when the disclosure is negative.

Sample size	The sample size was not statistically predetermined.<br>Samples used for RT-qPCR and ChIP-qPCR were harvested from independent three biological replicates, based on standard in the field.<br>We typically observed >150 nuclei at single live-imaging experiment, which is sufficient for the following statistical analysis. Sample sizes are indicated in the manuscript.
Data exclusions	No data was excluded from analysis.
Replication	Most of experiments were repeated multiple times.<br>In Supplementary Figure 7, 9, and 16, sufficient number of nuclei were obtained from a single embryo for further computational analysis.
Randomization	Not applicable. Used embryos were categorized by genotype before experiments.
Blinding	Not applicable. Quantitative analysis was not manually performed.

Reporting for specific materials, systems and methods

We require information from authors about some types of materials, experimental systems and methods used in many studies. Here, indicate whether each material, system or method listed is relevant to your study. If you are not sure if a list item applies to your research, read the appropriate section before selecting a response.

Materials & experimental systems

n/a	Involved in the study
	[x] Antibodies
[x]	Eukaryotic cell lines
[x]	Palaeontology and archaeology
	[x] Animals and other organisms
[x]	Clinical data
[x]	Dual use research of concern

Methods

n/a	Involved in the study
[x]	ChIP-seq
[x]	Flow cytometry
[x]	MRI-based neuroimaging

Antibodies

Antibodies used	Sheep polyclonal anti-digoxigenin antibody (Roche, 11093274910)<br>Mouse monoclonal anti-biotin (invitrogen, 03-3700, clone Z021)<br>Mouse monoclonal anti-dorsal (DSHB, AB_528204, clone 7A4)<br>Mouse monoclonal anti-RNA polymerase II (Millipore, 05-623, clone CTD4H8)<br>Alexa Fluor 555 donkey anti-sheep antibody (invitrogen, A-21436)<br>Alexa Fluor 488 donkey anti-mouse antibody (invitrogen, A-21202)
Validation	For both of anti-digoxigenin and -biotin antibody, application for FISH experiments was confirmed by previous publications (e.g., Yokoshi, M. et al., 2022). For anti-dorsal antibody, application for immunoprecipitation assay was confirmed by previous publications (e.g., Whalen, A. M. et al., 1993). For anti-RNA polymerase II antibody, application for immunoprecipitation assay was confirmed by previous publications (e.g., Lagha, M. et al., 2013).

Animals and other research organisms

Policy information about studies involving animals; ARRIVE guidelines recommended for reporting animal research, and Sex and Gender in Research

Laboratory animals	Species: Drosophila melanogaster<br>Strain: For production of transgenic strains, vk00033 line (BDSC #9750) was used.<br>For production of genome-edited strains, nanos-Cas9 strain was used.<br>Age: embryos
Wild animals	We did not use wild animals.
Reporting on sex	Sex of embryos was not discriminated.
Field-collected samples	We did not use field-collected samples.
Ethics oversight	No ethical approval was required for the research using D.melanogaster.

Note that full information on the approval of the study protocol must also be provided in the manuscript.
